# Water-Soluble, Titanocene-Based Prodrugs with Thiosemicarbazones as Cytotoxic Agents

**DOI:** 10.1021/acsomega.5c05481

**Published:** 2025-09-03

**Authors:** Kevin Schwitalla, Marie-Carlotta Müller, David Fabra, Marc Schmidtmann, Ulrike Meyer, Ana I. Matesanz, Adoracion G. Quiroga, Bernhard H. Rauch, Rüdiger Beckhaus

**Affiliations:** † Institute of Chemistry, Carl von Ossietzky University, 26129 Oldenburg, Germany; ‡ Pharmacology and Toxicology, University Medicine Oldenburg, Carl von Ossietzky University, 26129 Oldenburg, Germany; § Department of Inorganic Chemistry, 16722 Universidad Autonoma de Madrid, Cantoblanco, 28049 Madrid, Spain

## Abstract

Prodrugs that improve drug delivery in the body are highly desirable because they increase drug solubility and activity, thereby reducing drug concentration and side effects compared to the actual active compound. In this work, we demonstrate the impact of titanocene scaffolds on active thiosemicarbazones (TSCN). Two routes toward cationic Ti­(IV) TSCN complexes were established either by the reaction of titanocene bis­(trimethylsilyl)­acetylene with TSCN and subsequent oxidation of the resulting Ti­(III) complex with ferrocenium triflate or by ligand exchange of the triflato ligands in titanocene­(IV) triflate with TSCN. The solubility and stability of the complexes in aqueous media were evaluated by NMR and ultraviolet/visible (UV/vis) spectroscopy. A selection of cationic Ti­(IV) TSCN complexes exhibit improved water solubility, stability and increased cytotoxicity at lower concentrations compared to pure TSCN, cisplatin and 5-FU in human colon cancer cells *in vitro*.

## Graphical abstract

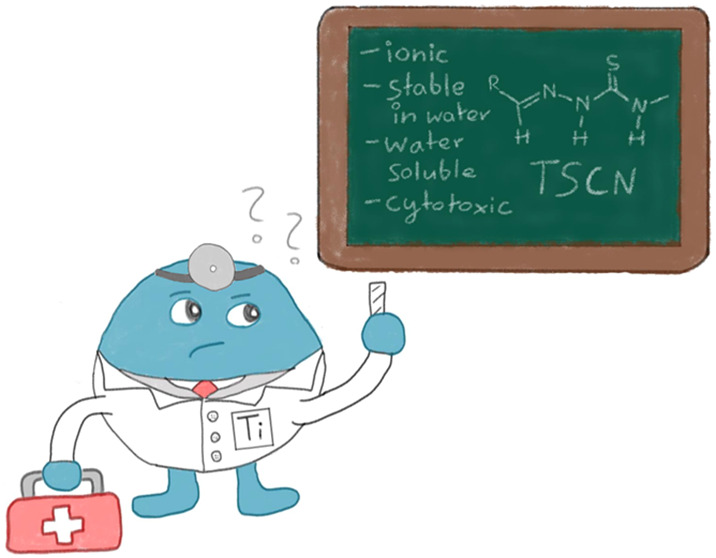

## Introduction

The design of water-soluble and water-stable complexes represents a major challenge in the development of titanium-based metallodrugs.^1^ Due to their high oxygen affinity, compounds often hydrolyze quickly in aqueous media,^2^ while the biological effects are highly dependent on the decomposition rate of the complex.^3^ Titanium-based metallodrugs are desirable because complexes of heavier transition metals, such as cisplatin, have high toxicity and side effects that are not present in titanium-based complexes.^4,5^ In 1979, Köpf and Köpf-Meyer reported the first titanium-based complex with anticancer properties—titanocene dichloride.^6^ Shortly thereafter, various other metallocene complexes, such as Titanocene Y,^7^ were studied,^8^ and amine-phenolato-,^3^ salen-type^9,10^ titanium complexes as well as the diketonato complex budotitan^11^ were introduced, which exhibit cytotoxic properties. However, these complexes have not progressed beyond the late stages of clinical trials, most likely because of their poor solubility,^12^ rapid hydrolysis in biological media^5,12,13^ or a lack of clinical response.^11,12,14^ Recently, water stable titanium complexes with transferrin mimetic ligands demonstrated the great promise of iron chelators as cytotoxic agents.^15^

The thiosemicarbazone (TSCN) Triapine is an alternate iron chelator that has entered many medical trials as a potential chemotherapeutic drug.^16–20^ TSCN in general are relevant types of ligands because of their role as ligands in coordination chemistry and their pharmacological properties.^21–27^ A wide range of antimicrobial,^28–30^ antiviral,^31–33^ and antitumor^34–39^ activities have been reported for TSCN.

Because of these biological activities, TSCN are exciting ligands for the development of (metallo)­prodrugs. The biological activities of TSCN and other compounds can be increased by metalation,^40^ therefore, TSCN metal complexes have attracted considerable attention. In particular, α-*N*-heterocyclic TSCN, such as Triapine, and their complexes show promising activities^41–43^ and have a well-studied mode of action, chelating the iron-center of mammalian ribonucleotide reductase.^44^ Recently, we reported the synthesis of thiosemicarbazone-based titanium complexes, but due to the insolubility and instability of these compounds in aqueous media, they were not suitable for biological studies.^45^ In this work, we report two routes leading to cationic TSCN-based Ti­(IV) complexes by combining TSCN ligands with the titanocene scaffold with the aim of developing water-soluble, water-stable and cytotoxic complexes (Figure 1).

**Figure 1:**
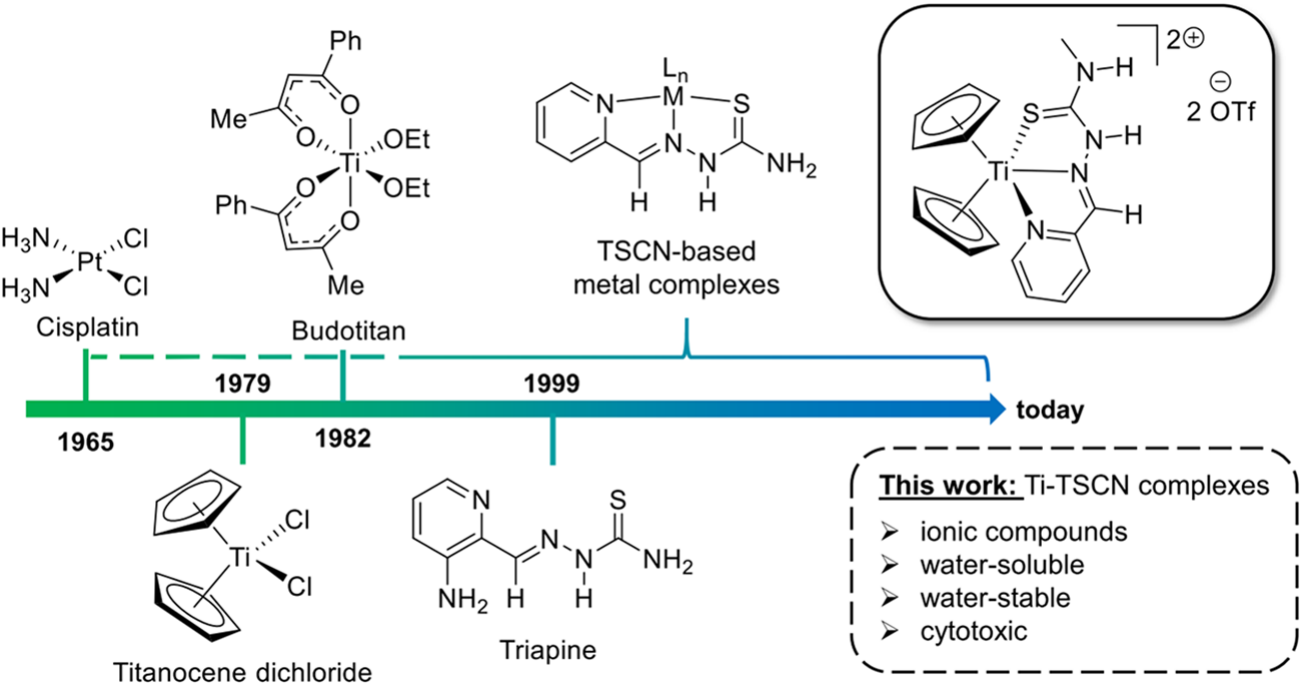
Timeline of relevant cytotoxic agents, cancerostatics and an example from this work.

## Results and Discussion

### Synthesis and Coordination Chemistry

The reactions of titanocene bis­(trimethylsilyl)­acetylene titanium complex **Ti1** with thiosemicarbazones **a**–**c** lead to the formation of Ti­(III) thiosemicarbazonato complexes **Ti1a**-**c** (Scheme 1, left). This occurs via redox reaction of the masked titanocene­(II) species with the acidic N^β^-proton of the TSCN via release of bis­(trimethylsilyl)­acetylene (BTMSA) and reduction of the proton to hydrogen. The κ^2^ *N* ^β^,*S* coordination mode of complexes **Ti1a**-**c** was determined by single-crystal X-ray crystallography (Figure 2), while the Ti­(III) nature of these compounds was measured by EPR spectroscopy (ESI, Figures S1–S3). The Ti­(III) complexes **Ti1a**-**c** were then oxidized with ferrocenium triflate to obtain the ionic Ti­(IV) thiosemicarbazonato triflate complexes **Ti2a**-**c** (Scheme 1, right).

**Figure 2:**
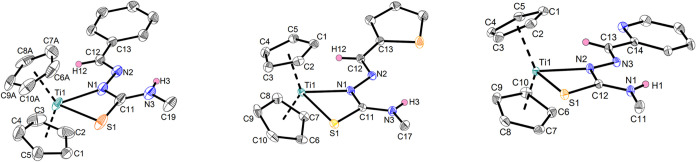
Molecular structures of complexes **Ti1a** (left), **Ti1b** (center) and **Ti1c** (right). Displacement ellipsoids are drawn at the 50% probability level. Redundant H atoms and solvent molecules have been omitted for clarity.

**Scheme 1:**
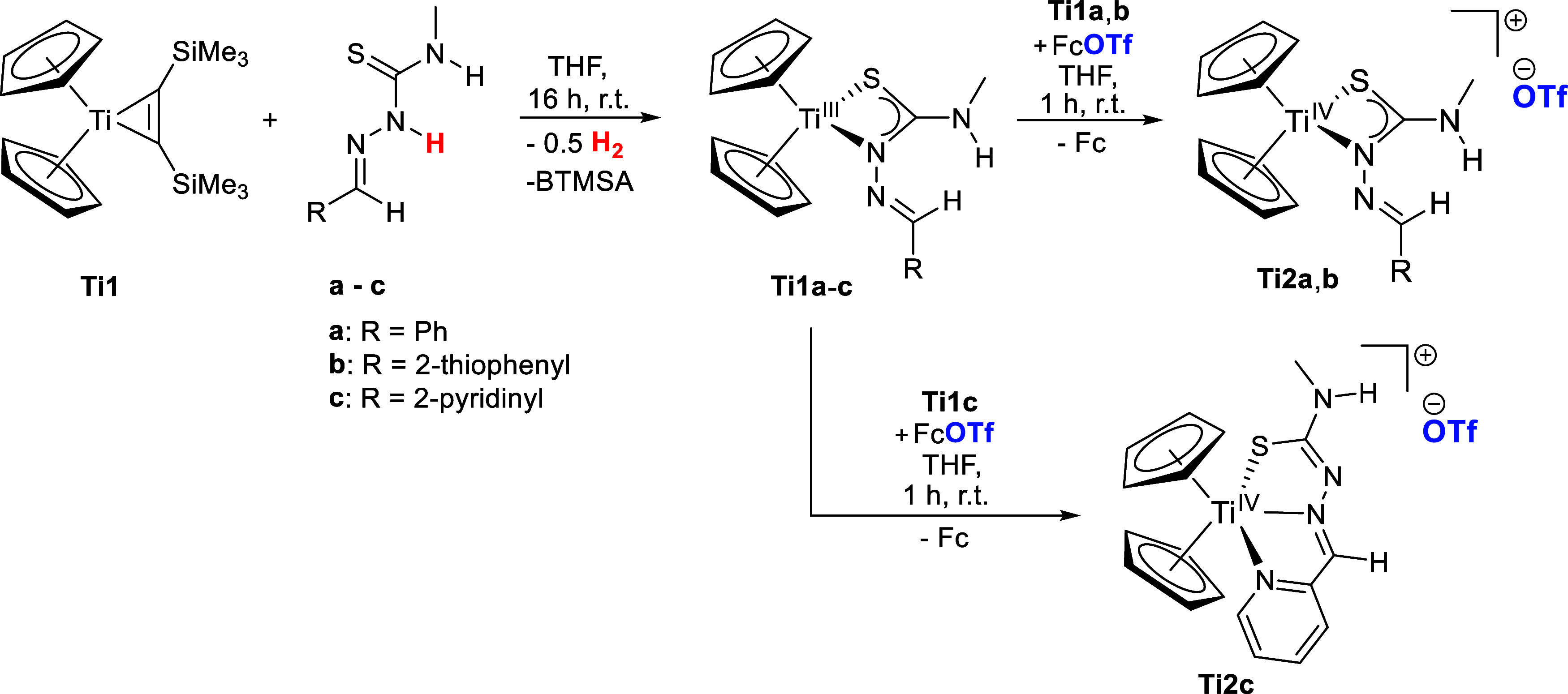
Reaction of Titanocene Bis­(trimethylsilyl)­acetylene **Ti1** with TSCN **a**-**c** to Obtain Ti­(III) Thiosemicarbazonato Complexes **Ti1a**-**c** ^a^

a Reaction of Ti1a-c with ferrocenium triflate to obtain cationic Ti­(IV) thiosemicarbazonato complexes Ti2a-c.

The coordination modes of **Ti2a**,**b** were identified by single-crystal X-ray diffraction in solid state (Figure 3). Both complexes maintain the κ^2^ *N* ^β^,*S* coordination mode of **Ti1a**-**c**, while **Ti2c** could not be crystallized. The potential donor site provided by the thiophene group of **Ti2b** shows no interaction with the metal.

**Figure 3:**
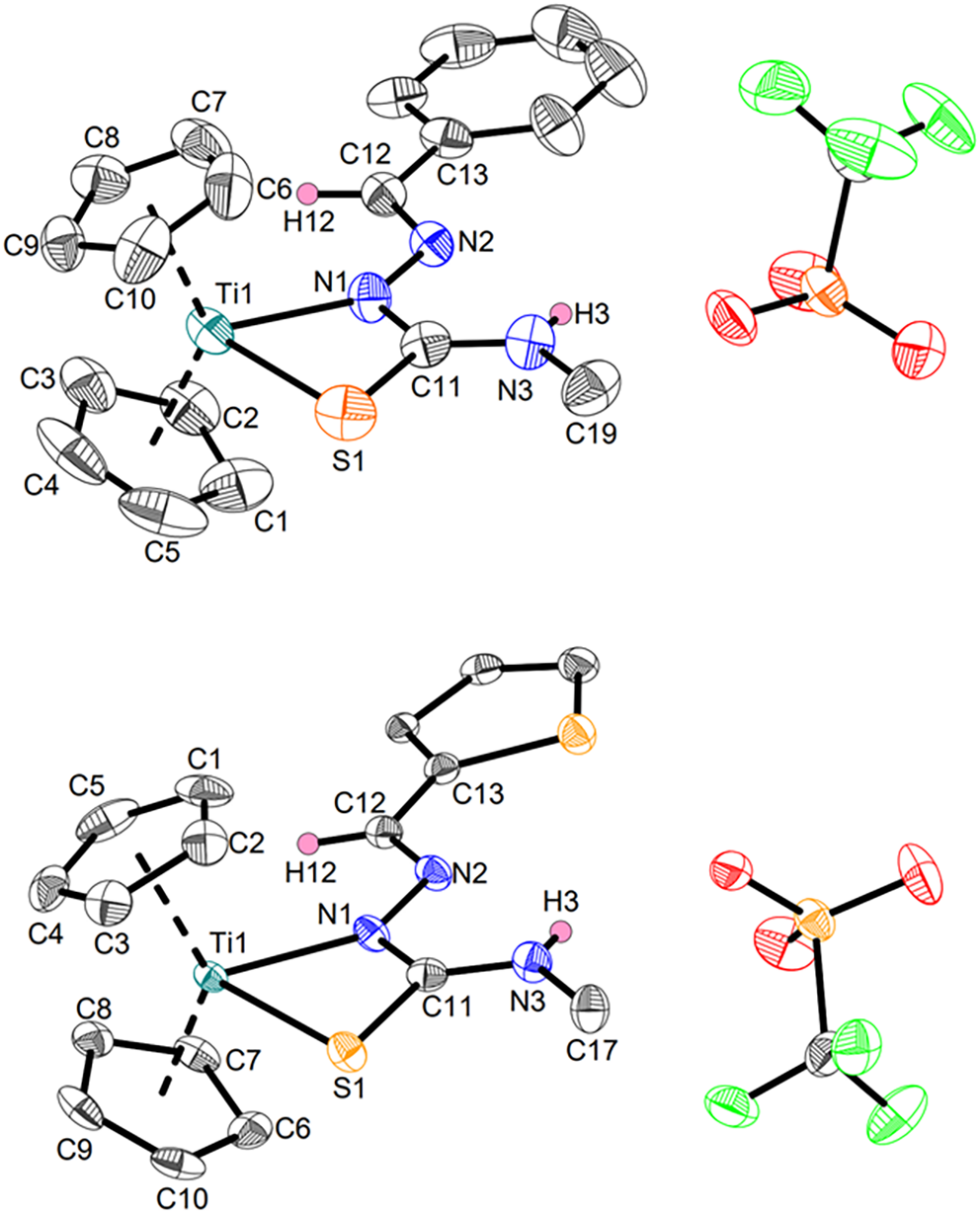
Crystal structures of complexes **Ti2a** (top) and **Ti2b** (bottom). Displacement ellipsoids are drawn at the 50% probability level. Redundant H atoms have been omitted for clarity.

However, the additional coordination of the pyridyl moiety of **Ti2c** was detected in the ultraviolet/visible (UV/vis) spectrum, as the pyridine-titanium charge transfer interaction band is visible at about 420–520 nm (**ESI**, Figure S4). This is responsible for the different colors of the respective solids (**Ti2a**,**b**: green, yellow, **Ti2c**: red) and is further evidence for a change of coordination mode of **Ti2c**. Since **Ti2c** could not be crystallized and to further evaluate the role of the anion, we compared the triflate anion of **Ti2c** with the [BPh_4_]^−^ anion. Complex **Ti1c** was oxidized with ferrocenium tetraphenylborate to obtain the isostructural complex **Ti3c** with [BPh_4_]^−^ anion (Scheme 2).

**Scheme 2:**
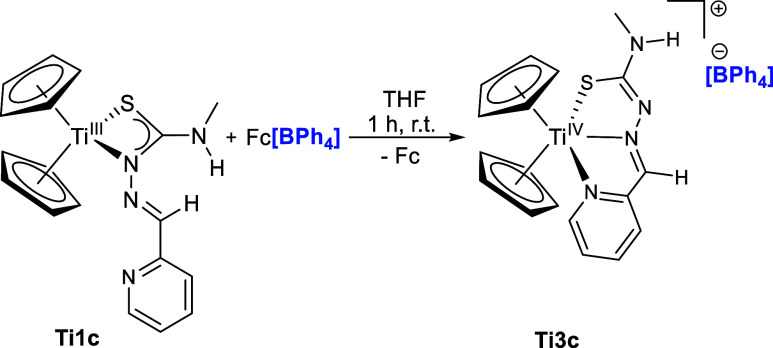
Reaction of Ti­(III) Complexes **Ti1a**-**c** with Ferrocenium Triflate to Obtain Cationic Ti­(IV) Thiosemicarbazonato Complexes **Ti2a**-**c**

The structure of **Ti3c** is confirmed by single-crystal X-ray diffraction (Figure 4), revealing the tridentate κ^3^ *N*,*N* ^α^,*S* coordination mode of the TSCN, which can most likely also be assigned to **Ti2c** due to the noncoordinating role of the triflate anion in **Ti2a**,**b**. The crystal structure of **Ti3c** shows the additional coordination of the pyridine ligand and a change of the coordinating nitrogen from N^β^ to N^α^ that was also detected for **Ti2c**.

**Figure 4:**
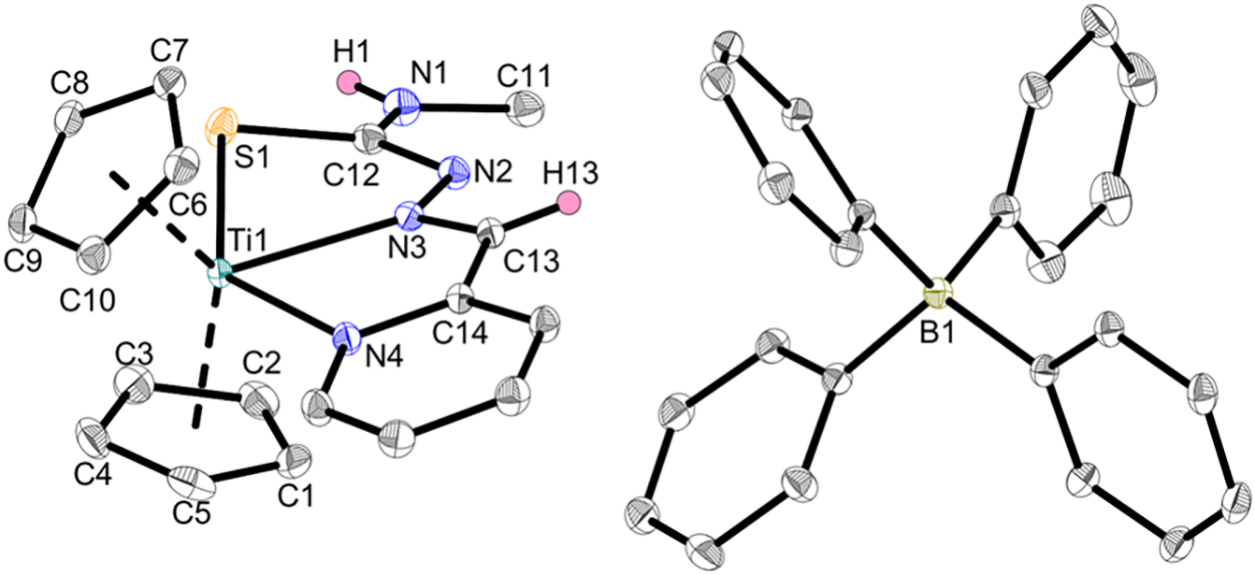
Crystal structure of complex **Ti3c**. Displacement ellipsoids are drawn at the 50% probability level. Redundant H atoms and solvent molecules have been omitted for clarity.

Based on the strong chelating behavior of TSCN **c**, we attempted to further enhance the water solubility by creating a dicationic system. This was realized by a ligand exchange reaction of titanocene­(IV) triflate with TSCN **c**, in which the two coordinating triflato ligands are displaced by the TSCN to yield the dicationic κ^3^ *N*,*N* ^α^,*S* complex **Ti4c** with two triflate anions (Scheme 3). The κ^3^ *N*,*N* ^α^,*S* coordination mode of **Ti4c** was confirmed by single-crystal X-ray diffraction (Figure 5) in solid state and by NMR in solution, as the pyridine ^15^N NMR chemical shift of 257.8 ppm correlates with a coordinating pyridine moiety.^46^

**Figure 5:**
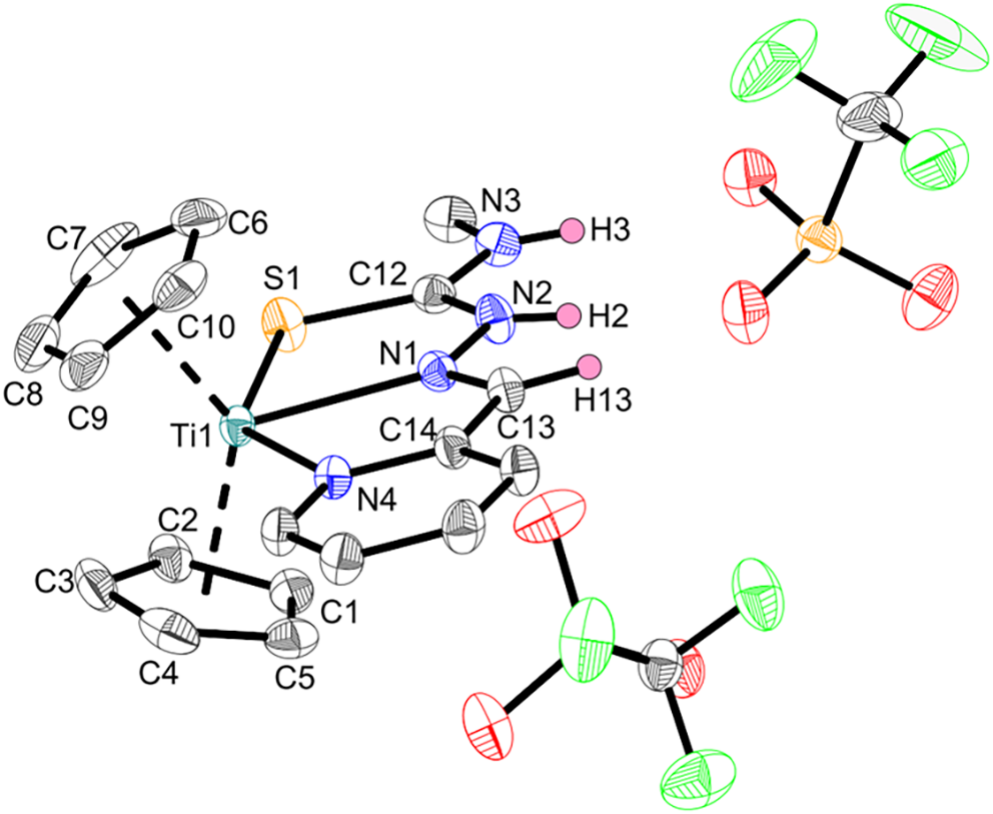
Crystal structure of complex **Ti4c**. Displacement ellipsoids are drawn at the 50% probability level. Redundant H atoms and solvent molecules have been omitted for clarity.

**Scheme 3:**
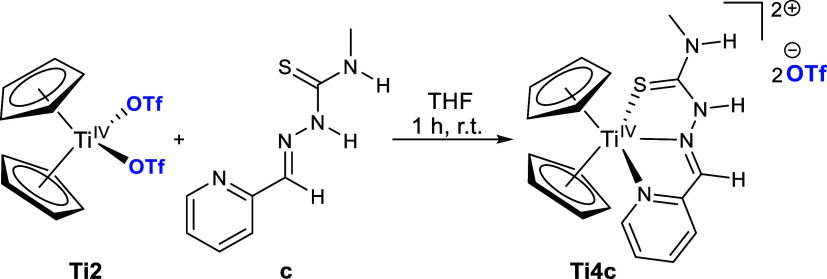
Reaction of Cp_2_Ti­(OTf)_2_ (**Ti2**) with TSCN **c** to Obtain Dicationic Ti­(IV) Thiosemicarbazone Complex **Ti4c**

### Water-Solubility and Stability of the Complexes

To be suitable for biological assays, the complexes must be sufficiently soluble and stable in aqueous media. Neutral complexes, such as **Ti1a**-**c**, are insoluble in water, while the cationic triflate complexes **Ti2a**-**c** and **Ti4c** are soluble to some extent. The effect of triflate anions on water-solubility is highlighted by complex **Ti3c**, which has a [BPh_4_]^−^ counterion instead of a triflate. Despite being a cationic complex, it is insoluble in water, possibly caused by the lipophilic nature of the [BPh_4_]^−^ counterion. The formal 18 electron complexes **Ti2c** and **Ti4c** are significantly less reactive with respect to substitution reactions than the formal 16 electron complexes **Ti2a**,**b**. The stability studies performed by NMR spectroscopy showed hydrolyzed species in all the complexes, but **Ti2c** and **Ti4c** were the only compounds which were detected for at least 4 h (ESI, Figures S10 and S11). Compounds **Ti2a** and **Ti2b** show immediate hydrolysis (ESI, Figures S12 and S13). The hydrolysis reactions of **Ti2c** and **Ti4c** were additionally followed by UV/vis experiments in deionized water and in aqueous buffers (ESI, Figures S4–S9), coinciding with the NMR experiments. Due to their stability in aqueous media, complexes **Ti2c** and **Ti4c** are the best choice for cytotoxicity studies.

The hydrolysis products were identified by ^1^H NMR spectroscopy by comparing the chemical shifts of the Cp signals in D_2_O with the hydrolysis products of Cp_2_Ti­(OTf)-μO-Cp_2_Ti­(OTf)^47^ (ESI, Figure S14) and the Cp_2_Ti­(OTf)_2_ precursor (ESI, Figure S15). The monocationic complexes **Ti2a**-**c** hydrolyze to [Cp_2_Ti­(D_2_O)-μO-Cp_2_Ti­(D_2_O)]­(OTf)_2_ with a ^1^H NMR chemical shift of 6.47 ppm, while complex **Ti4c** hydrolyzes to [Cp_2_Ti­(D_2_O)_2_]­(OTf)_2_ with 6.62 ppm. The formation of the μO-complex is most likely due to the anionic thiosemicarbazonato ligands in **Ti2a**-**c**, which deprotonate water to form TSCN and a hydroxo-intermediate that subsequently reacts to the μO-complex (Scheme 4, top). In contrast, the neutral TSCN ligand in **Ti4c** is displaced by aqua ligands, thus, releasing TSCN **c** and forming [Cp_2_Ti­(D_2_O)_2_]­(OTf)_2_ (Scheme 4, bottom).

**Scheme 4:**
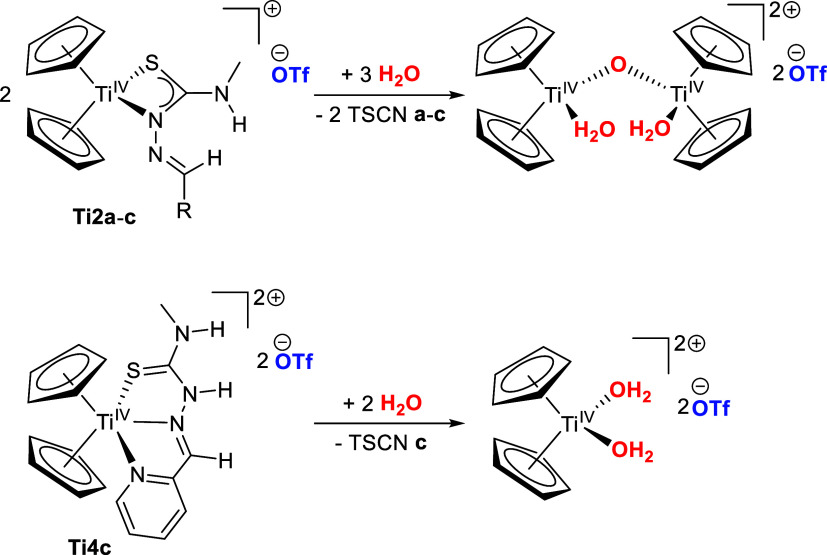
Proposed Hydrolysis Reactions of **Ti2a**-**c** and **Ti4c**

Regarding the water solubility of both candidates, **Ti4c** is significantly more soluble (≈ 30 mg/mL) compared to **Ti2c** (≈ 0.1 mg/mL). This is due to the charge of the complexes as **Ti2c** is monocationic with one anion, while **Ti4c** is dicationic with two triflate anions. However, in contrast to the pure TSCN **c**, which is practically insoluble in water, the complexes are a significant improvement in the solubility of TSCN.

This study shows that the solubility of the complexes in aqueous media depends on the charge and the counterions of the complex, while the stability of the complexes depends on the coordination modes.

### Cytotoxicity Studies

The screening of the newly synthesized complexes **Ti2c** and **Ti4c** was performed in the human colon cancer cell line HCT116 at various concentrations (0.1 μM up to 300 μM) by MTT assay analysis of mitochondrial activity and proliferation ability.^48^ The analysis revealed that both complexes and the control substances exhibited significant cytotoxic activity in comparison to the untreated control. Of particular interest is the significant increase of cytotoxicity of the synthesized compounds **Ti2c** and **Ti4c** compared to the TSCN ligand at a concentration of 10 μM (Figure 6). This difference is most likely due to the insolubility of TSCN **c** in aqueous media, which results in a lower availablility in comparison with the water-soluble complexes **Ti2c** and **Ti4c**. TSCN c showed no significant cytotoxicity at low concentrations (1 to 10 μM) in HCT116 cells, as cell cytotoxicity fluctuates below zero, suggesting low antimetabolic activity under the given experimental conditions. The two newly synthesized compounds exhibit a higher level of cytotoxic activity at a concentration of 10 μM in comparison to the commonly employed cytotoxic compound, cisplatin. At a concentration of 100 μM, the tested compounds exhibit comparable cytotoxicity, particularly when compared to 5-FU and the TSCN ligand alone. TSCN derivatives have shown to act on other colorectal cancer cell lines around the same concentration of administration.^49^

**Figure 6:**
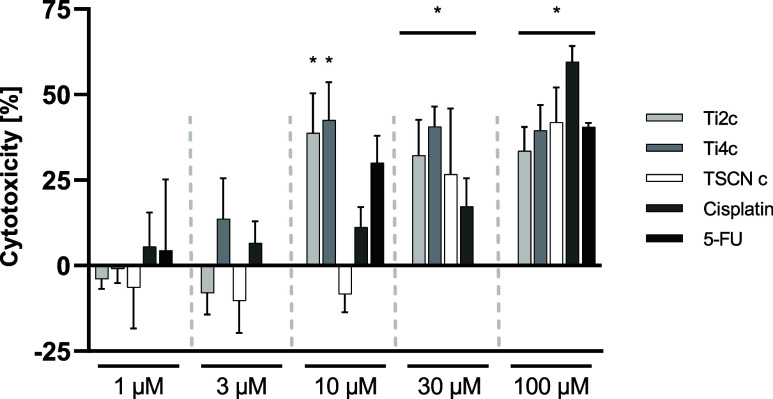
Cytotoxicity of different compounds on colon cancer cell line HCT116 after 24 h established by MTT assay. Cisplatin was used as a comparative positive control. Values are expressed as mean ± SD, Quantification from *n* = 4 to *n* = 8, * *p*-value <0.05 compared to PBS control. Effect of 5-FU was measured only at concentrations of 1, 10, and 100 μM.

At concentrations of 10 and 100 μM, titanocene­(IV) triflate Cp_2_Ti­(OTf)_2_ exhibits minimal, nonsignificant cellular toxicity (not shown), indicating that the release of TSCN is indeed responsible for the observed cytotoxic effects. As demonstrated in Table 1, the aforementioned effects can also be observed in the IC_50_ values. **Ti2c** shows an IC_50_ value of 8.13 μM, in comparison to 23.22 μM for the TSCN **c** precursor. The IC_50_ value of **Ti4c** is found to be the lowest at 3.20 μM. In comparison, the IC_50_ value of cisplatin is reported as 33.76 μM in HCT116 cells. The titanium based compound titanocene dichloride was also tested. In this study, an IC_50_ value of 564.5 μM was determined. This finding is consistent with the observation of similarly elevated IC_50_ values for this compound in other cell lines.^50,51^ Both **Ti2c** and **Ti4c** (already at 10 μM) significantly affect the metabolic activity of HCT116 colon cancer cells, as determined by MTT assay, following an incubation period of 24 h (Figure 6). This finding indicates the potential of these compounds to exhibit anticancer activity.

**Table 1:** IC_50_ Best Fit Values for Test Compounds and Control Compounds Based on Logarithmic Nonlinear Regression (Four Parameters) of Cytotoxicity Data

compound	IC_50_ value [μM] for HTC116
Ti2c	8.13
Ti4c	3.20
TSCN c	23.22
cisplatin	33.76
titanocene dichloride	564.5

### Induction of Apoptosis

Due to the significant reduction of cell viability indicated by mitochondrial activity, we evaluated the effects on apoptosis induction by Annexin V/PI staining of HCT116 cells treated with the compounds **Ti2c** and **Ti4c**. No nominable effect was visible after 24 h on apoptosis and necrosis (Data not shown). After an incubation period of 48 h the cells showed a distinct increase in apoptotic cell numbers, but only a negligible number of cells was shown to enter necrotic cell death (Figure 7). This underlines the mechanism of action associated with TSCN, as the inhibition of the ribonucleotide reductase enzyme only becomes apparent after prolonged incubation on proliferating cells.^52^ The obtained results indicate that the HCT116 cells undergo controlled apoptosis, as evidenced by the presence of a significant late apoptotic population (Figure 7). Here, the **Ti2c** compound induced a rate of 32.26 ± 4,03% late apoptotic cells compared to 2.21% necrotic cells, similar to the effects of 5-FU with 20.00 ± 5.40% late apoptotic cells and 1.32 ± 0.09% necrotic cells. **Ti4c** showed lower induction of late apoptosis at 11.96 ± 2.73% compared to 1.15 ± 0.16%. Our observation may possibly indicate a protracted apoptotic effect of the TSCN complexes rather than a direct necrosis-inducing effect. This may be associated with a gentle antineoplastic effect and could be favorable for a perspective clinical use.^53^

**Figure 7:**
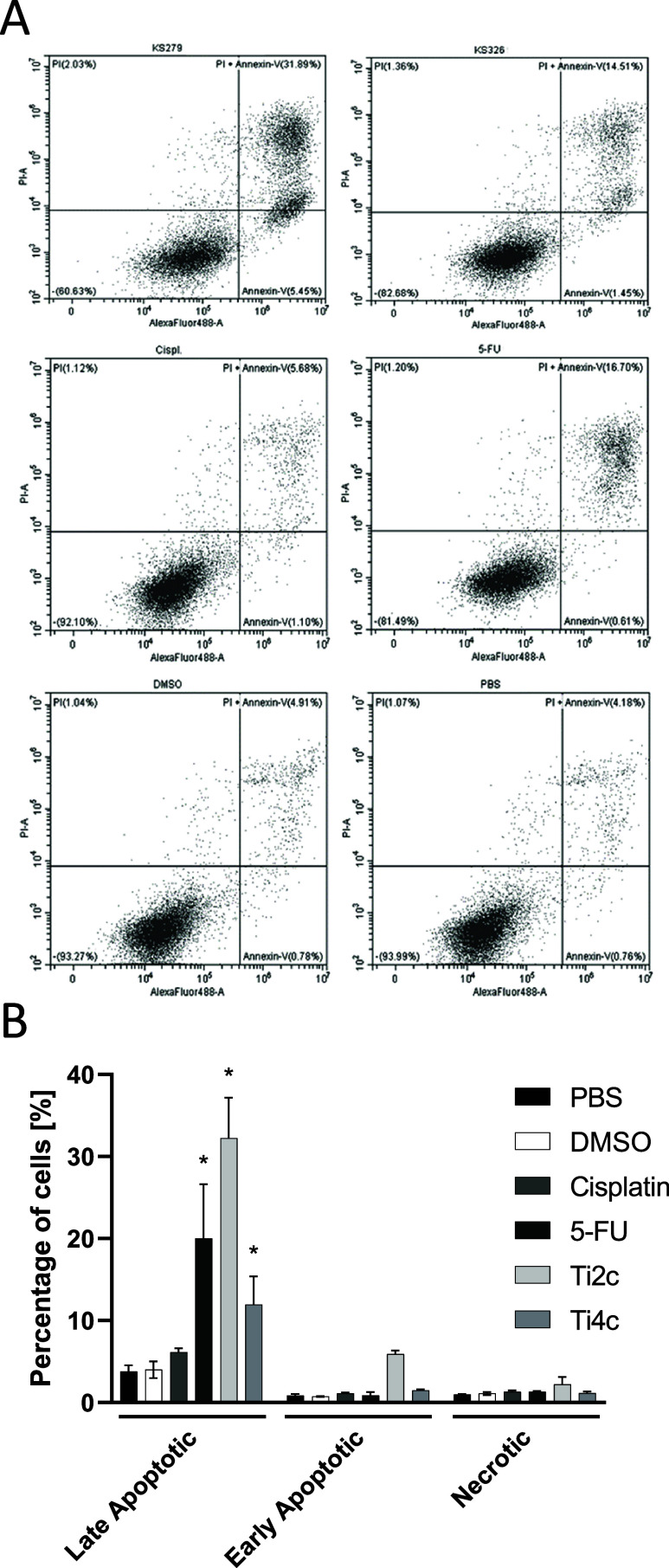
Apoptotic effect of the synthesized compounds quantified by Annexin V–PI staining and flow cytometry analysis. HCT116 cells were incubated with **Ti2c**, **Ti4c** or Cisplatin, 5-FU at 10 μM or the corresponding controls over 48 h. (A) Examples of original flow cytometry measurements as labeled above in the respective scatter-plot. PI = Necrotic cells, PI + Annexin = Late Apoptotic cells, – = Live cells, Annexin-V = Early Apoptotic cells. Enlarged plots in ESI, Figure S60. (B) Quantification from *n* = 3. Values are expressed as mean ± SD, * = *p*-value <0.0001 compared to DPBS control. KS279 = **Ti2c**, KS329 = **Ti4c**.

### Cellular Uptake

To further evaluate the transport mechanism, we performed cellular uptake analysis by ESI-MS^54^ of incubated, isolated cells that were treated with either **Ti2c** or **Ti4c** for different time periods (0 h = control, 0.5, 1, 4 h). The mass spectra of pure TSCN **c**, triflic acid, **Ti2c**, **Ti4c** and HCT116 cells only were used as references (ESI, Figures S16–S21). The triflate anion is identified either in the negative ESI mode or as HOTf+Na^+^ in the positive mode, **c** was identified either neutrally or as **c** + Na^+^ and the cationic titanium species of **Ti2c** and **Ti4c** were both identified as the monocationic [Ti-TSCN]^+^ species. Other titanocene fragments were not detected by this method. After 1 h of treatment with either **Ti2c** or **Ti4c**, TSCN **c** and triflate anions were detected in the isolated cells (ESI, Figures S23, S24, S29, and S30), indicating a transport of the complexes into the cells. This was not observed in the control experiments (0 h) or after 0.5 h. A longer treatment (e.g., after 4 h) shows similar results, while also demonstrating the persistance of TSCN **c** and triflate anions inside the cells. However, the [Ti-TSCN]^+^ species was not detected in any case, which is most likely due to rapid hydrolysis within the cells or further interactions of the titanocene species with cell contents. Nevertheless, in combination with the increased cytotoxicity at 10 μM compared to TSCN **c**, the titanocene scaffold has an impact on the transport of the active TSCN into the cells and thus on the biological availablility of the active drug.

### Gel Electrophoresis Analysis

DNA interaction is the basis of the mechanism of action of many metallodrugs. For instance, classical Pt­(II) metal complexes, like cisplatin, form covalent bonds with DNA through the N7 of the guanine, while titanocene dichloride interacts through the phosphoesters.^55^ Agarose gel electrophoresis of plasmid vectors such as pBR322 is a powerful technique that allows to distinguish between covalent and noncovalent modes of interaction. pBR322 is predominantly isolated in a supercoiled (SC) state, and manipulation by damaging agents gives rise to the open-circular (OC) and linear (L) isoforms.^56^

The results of the gel electrophoresis of pBR322 in SC form after incubation with the complexes can be seen in Figure 8. Cisplatin was included as a positive control (lanes 3 to 7 in both cases) and shows the expected behavior for a covalent binder: the relative electrophoretic mobility of the SC form is reduced, giving rise to the OC form whose mobility is increased due to platination. Both forms comigrate at *r*_i_ ∼ 0.20 (lane 6 in both cases). The precursors **c** and **Ti2** (lanes 8 to 12 and 13 to 17 in Figure 8A, respectively) produce a slight unwinding of the SC form but overall do not change the electrophoretic mobility of any of the isoforms. In contrast, complexes **Ti2c** and **Ti4c** (lanes 8 to 12 and 13 to 17 in Figure 8B, respectively) unwind the DNA to a greater extent in a dose-dependent manner, being maximum already at *r*_i_ = 0.05 (lanes 9 and 14 for **Ti2c** and **Ti4c**, respectively). They also maintain the relative mobilities of both SC and OC isoforms, which suggests a noncovalent mode of interaction with DNA in all cases.

**Figure 8:**
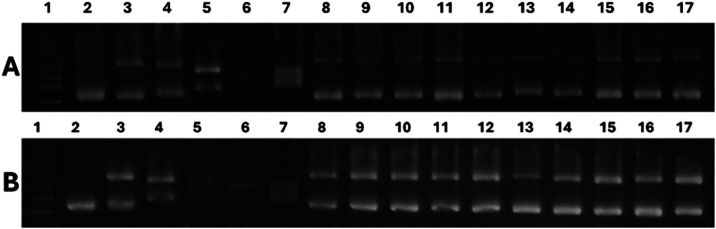
Gel electrophoresis assay with plasmid pBR322 (*C*_DNA_ = 0.0625 μg μL^–1^) after 24 h incubation with increasing concentrations of cisplatin (lanes 3 to 7, *r*_i_: 0.01 to 0.25), (**A**) TSCN **c** (lanes 8 to 12, *r*_i_: 0.01 to 0.25), **Ti2 (**Cp_2_Ti­(OTf)_2_) (lanes 13 to 17, *r*_i_: 0.01 to 0.25); or (**B**) **Ti2c** (lanes 8 to 12, *r*_i_: 0.01 to 0.25), **Ti4c** (lanes 13 to 17, *r*_i_: 0.01 to 0.25). Lane 1 and 2 in both cases contain a 1 kb DNA ladder and untreated pBR322 control, respectively.

## Summary and Conclusions

This work demonstrates the synthesis and effects of titanocene-based prodrugs with thiosemicarbazone ligands. Monocationic Ti­(IV) complexes were obtained by oxidation of Ti­(III) thiosemicarbazonato complexes with ferrocenium salts, while a dicationic Ti­(IV) thiosemicarbazone complex was prepared by ligand displacement of both triflato ligands of titanocene­(IV) triflate with an α-*N*-heterocyclic TSCN ligand. The 18-electron κ^3^ *N*,*N* ^α^,*S*-complexes showed good water solubility, sufficient stability in aqueous media and were suitable for cytotoxicity studies. At low concentrations, the titanium-based prodrugs showed significantly superior performance compared to the active TSCN ligand, cisplatin and 5-FU. However, at higher concentrations, the performance of the prodrugs is surpassed by these established agents. Mechanism of action analysis by gel electrophoresis showed an expected covalent binding of cisplatin to DNA. A comparable effect was not observed for the complexes **Ti2c** and **Ti4c**, indicating that covalent binding did not occur. It is rather likely that the characteristic iron chelating mechanism of action of the active TSCN ligand applies for these complexes.^57^ In addition, we have conducted in-depth analyses of the hydrolysis products and the mechanism of action of our novel metalloprodrugs. It has been demonstrated that the new titanium-based compounds exhibit a cytotoxic effect in cultured colon cancer cells. This effect is associated with the triggering of apoptosis in the cells. Compared to the clinically established substances cisplatin and 5-FU, a negative effect on metabolic activity and apoptotic effect was evident at lower concentrations. However, direct necrotic effects were not observed. Further studies are needed to determine the potential of titanium-based prodrugs as potential antineoplastic drugs and their detailed mechanisms of action.

## Experimental Section

All reactions were carried out under a dry nitrogen or argon atmosphere using standard Schlenk and glovebox techniques. Solvents were dried according to standard procedures over Na/K alloy with benzophenone as indicator and subsequently distilled and stored under a nitrogen atmosphere. Titanocenbis­(trimethylsilyl)­acetylene,^58^ Cp_2_Ti­(OTf)-μO-Cp_2_Ti­(OTf),^47^ Cp_2_Ti­(OTf)_2_,^59^ Fc­(OTf),^60^ Fc­(BPh_4_),^61^ and TSCN^62^ were prepared according to general methods and published procedures. NMR spectra were recorded on a Bruker AVANCE III 500 spectrometer (^1^H 500 MHz). IR spectra were recorded on a Bruker Tensor 27 spectrometer using an attenuated total reflection (ATR) method. Elemental analyses were carried out on a Euro EA 3000 Elemental Analyzer. Melting points were determined using a “Mel-Temp” from Laboratory Devices, Cambridge, or a Mettler Toledo MP30. UV/vis spectra were recorded on an Agilent Cary 60 spectrophotometer. High-resolution mass spectra were measured on a Finnigan-MAT95 spectrometer in methanol using ESI. Further exact details of NMR spectra (S33–S44), crystallographic data (S45–S51), IR (S52–S59), EPR, UV/vis and mass spectra are given in the Electronic Supporting Information (ESI).

### Syntheses and Characterization

#### Synthesis of **Ti1a**

Titanocenbis­(trimethylsilyl)­acetylene titanium complex **Ti1** (300 mg, 0.861 mmol) and benzaldehyde *N*-methylthiosemicarbazone **a** (166 mg, 0.861 mmol) were dissolved in 10 mL of dry THF. The reaction mixture was stirred for 16 h at room temperature to give a dark purple solution. The solvent was removed under reduced pressure and the residue was washed with 10 mL of *n*-hexane. All volatile components were removed under reduced pressure and the residue was dried under vacuum to yield the product as a gray solid. Purple crystals suitable for single crystal X-ray diffraction analysis precipitated from a saturated solution of **Ti1b** in toluene at −20 °C after several days. **Yield**: 0.230 g, 0.621 mmol, 72%. **IR** (ATR): $\tilde{V}$ = 3367, 2932, 1596, 1569, 1533, 1442, 1375, 1328, 1286, 1218, 1155, 1086, 1066, 1020, 1009, 933, 836, 790, 755, 694, 655, 562, 514, 502, 432 cm^–1^. **Mp**. 183 °C (dec.). **EPR**: *g* = 1.980. **EA**: calcd for C_19_H_20_N_3_STi: C 61.62, H 5.44, N 11.35. Found: C 61.30, H 5.19, N 10.88.

#### Synthesis of **Ti1b**

Titanocenbis­(trimethylsilyl)­acetylene titanium complex **Ti1** (300 mg, 0.861 mmol) and 2-thiophenecarboxaldehyde *N*-methylthiosemicarbazone **b** (172 mg, 0.861 mmol) were dissolved in 10 mL of dry THF. The reaction mixture was stirred for 16 h at room temperature to give a dark purple solution. The solvent was removed under reduced pressure and the residue was washed with 10 mL of *n*-hexane. All volatile components were removed under reduced pressure and the residue was dried under vacuum to yield the product as a gray solid. Purple crystals suitable for single crystal X-ray diffraction analysis precipitated from a slowly evaporating solution of **Ti1b** in benzene/toluene after several days. **Yield**: 0.278 g, 0.737 mmol, 86%. **IR** (ATR): $\tilde{V}$ = 3375, 3102, 2931, 2360, 2324, 1705, 1580, 1531, 1431, 1373, 1352, 1329, 1291, 1262, 1231, 1162, 1124, 1079, 1045, 1013, 934, 858, 804, 783, 753, 691, 653, 568, 506 cm^–1^. **Mp**. 148 °C (dec.). **EPR**: *g* = 1.983. **EA**: calcd for C_17_H_18_N_3_S_2_Ti: C 54.25, H 4.82, N 11.17. Found: C 53.85, H 5.10, N 10.70.

#### Synthesis of **Ti1c**

Titanocenbis­(trimethylsilyl)­acetylene titanium complex **Ti1** (500 mg, 1.43 mmol) and 2-pyridinecarboxaldehyde *N*-methylthiosemicarbazone **c** (279 mg, 1.43 mmol) were dissolved in 10 mL of dry THF. The reaction mixture was stirred for 16 h at room temperature to give a dark purple solution. The solvent was removed under reduced pressure and the residue was washed with 10 mL of *n*-hexane. All volatile components were removed under reduced pressure and the residue was dried under vacuum to yield the product as a purple solid. Purple crystals suitable for single-crystal X-ray diffraction analysis precipitated from a saturated THF/*n*-hexane solution of **Ti1c** at −20 °C after several days. **Yield**: 0.278 g, 0.737 mmol, 86%. **IR** (ATR): $\tilde{V}$ = 3242, 1580, 1530, 1468, 1433, 1367, 1322, 1311, 1277, 1147, 1100, 1077, 1021, 1012, 991, 928, 794, 770, 741, 715, 671, 621, 579, 520 cm^–1^. **Mp**. 197 °C (dec.). **EPR**: *g* = 1.983. **EA**: calcd for C_18_H_19_N_4_STi: C 58.23, H 5.16, N 15.09. Found: C 57.71, H 5.34, N 14.79.

#### Synthesis of **Ti2a**

Complex **Ti1a** (100 mg, 0.270 mmol) and ferrocenium triflate (90.5 mg, 0.270 mmol) were dissolved in 10 mL of dry THF. The reaction mixture was stirred for 2 h at room temperature to give a yellow green solution. The solvent was reduced to ca. 3 mL and deluted with 10 mL of *n*-hexane. A green solid precepitated and the supernatant was decanted. The residue was washed with *n*-hexane (2 × 10 mL). All volatile components were removed under reduced pressure and the residue was dried under vacuum to yield the product as a green solid. NMR data is omitted due to poor solubility in all standard solvents. Green crystals suitable for single crystal X-ray diffraction analysis precipitated from a slowly evaporating solution of **Ti2a** in C_6_D_6_ after several days. **Yield**: 0.113 g, 0.218 mmol, 81%. **IR** (ATR): $\tilde{V}$ = 3315, 3102, 2359, 1557, 1489, 1439, 1373, 1323, 1275, 1253, 1223, 1151, 1092, 1071, 1027, 960, 830, 801, 756, 729, 691, 658, 636, 571, 515 cm^–1^. **Mp**. 181 °C (dec.). **EA**: calcd for C_20_H_20_F_3_N_3_S_2_O_3_Ti: C 46.25, H 3.88, N 8.09. Found: C 45.72, H 3.76, N 7.75.

#### Synthesis of **Ti2b**

Complex **Ti1b** (100 mg, 0.270 mmol) and ferrocenium triflate (88.8 mg, 0.270 mmol) were dissolved in 10 mL of dry THF. The reaction mixture was stirred for 2 h at room temperature to give a yellow green solution. The solvent was reduced to ca. 3 mL and deluted with 10 mL of *n*-hexane. A green solid precepitated and the supernatant was decanted. The residue was washed with *n*-hexane (2 × 10 mL). All volatile components were removed under reduced pressure and the residue was dried under vacuum to yield the product as a green solid. Green crystals suitable for single-crystal X-ray diffraction analysis precipitated from a saturated THF/toluene solution of **Ti2b** at −20 °C after several days. **Yield**: 0.097 g, 0.185 mmol, 70%. ^**1**^ **H NMR** (500 MHz, C_6_D_6_, 305 K): δ = 2.69 (d, *J* = 4.9 Hz, 3 H, N-Me), 5.94 (s, 10 H, Cp-H), 6.46–6.48 (m, 1 H, N–H), 6.58–6.61 (m, 1 H, Ar–H), 6.66–6.68 (m, 1 H, Ar–H), 6.74–6.76 (m, 1 H, Ar–H), 8.51 (s, 1 H, aldimine-H) ppm. ^**19**^ **F­{** ^**1**^ **H} NMR** (470 MHz, 305 K, C_6_D_6_): δ = −77.4 ppm. **IR** (ATR): $\tilde{V}$ = 3339, 3107, 1578, 1557, 1526, 1506, 1436, 1406, 1370, 1323, 1276, 1250, 1222, 1158, 1084, 1059, 1047, 1028, 946, 860, 829, 757, 727, 713, 701, 634, 572, 516 cm^–1^. **Mp**. 199 °C (dec.). **EA**: calcd for C_18_H_18_F_3_N_3_S_3_O_3_Ti: C 41.15, H 3.45, N 8.00. Found: C 40.51, H 3.37, N 7.78.

#### Synthesis of **Ti2c**

Complex **Ti1c** (100 mg, 0.270 mmol) and ferrocenium triflate (90.2 mg, 0.270 mmol) were dissolved in 10 mL of dry THF. The reaction mixture was stirred for 2 h at room temperature to give a red solution. The solvent was reduced to ca. 3 mL and deluted with 10 mL of *n*-hexane. A red solid precepitated overnight and the supernatant was decanted. The residue was again diluted with 3 mL of dry THF and then diluted with 10 mL of *n*-hexane. A red solid precepitated overnight and the supernatant was decanted. The residue was washed with *n*-hexane (2 × 10 mL). All volatile components were removed under reduced pressure and the residue was dried under vacuum to yield the product as a red solid. **Yield**: 0.101 g, 0.194 mmol, 72%. ^**1**^ **H NMR** (500 MHz, THF-*d*_8_, 298 K): δ = 2.92 (m, 3 H, N-Me), 6.31 (s, 10 H, Cp-H), 7.65–7.70 (m, 1 H, Ar–H), 7.73–7.77 (m, 1 H, Ar–H), 8.05–8.10 (m, 1 H, Ar–H), 8.32–8.44 (m, 1 H, Ar–H), 9.15 (s, 1 H, aldimine-H) ppm. The N–H signal was not found. ^**13**^ **C­{** ^**1**^ **H} NMR** (125 MHz, 298 K, THF-*d*_8_): δ = 31.8 (N-Me), 117.6 (Cp-CH), 120.0 (Ar–CH), 126.6 (Ar–CH), 126.8 (Ar–CH), 140.8 (Ar–CH), 156.5 (aldimine-CH) ppm. ^**19**^ **F­{** ^**1**^ **H} NMR** (470 MHz, 298 K, THF-*d*_8_): δ = −80.4 ppm. **IR** (ATR): $\tilde{V}$ = 3305, 3115, 2942, 2360, 2324, 2287, 2163, 1583, 1561, 1540, 1509, 1466, 1446, 1397, 1352, 1248, 1223, 1154, 1116, 1081, 1027, 908, 827, 775, 750, 635, 572, 516 cm^–1^. **Mp**. 101 °C (dec.). **EA**: calcd for C_19_H_19_F_3_N_4_O_3_S_2_Ti: C 43.85, H 3.68, N 10.77. Found: C 44.39, H 3.56, N 10.56.

#### Synthesis of **Ti3c**

Complex **Ti1c** (100 mg, 0.269 mmol) and ferrocenium tetraphenyl borate (136 mg, 0.269 mmol) were dissolved in 10 mL of dry THF. The reaction mixture was stirred for 2 h at room temperature to give a red solution. The solvent was reduced to ca. 3 mL and deluted with 10 mL of *n*-hexane. A red solid precepitated overnight and the supernatant was decanted. The residue was again diluted with 3 mL of dry THF and then diluted with 10 mL of *n*-hexane. A red solid precepitated overnight and the supernatant was decanted. The residue was washed with *n*-hexane (2 × 10 mL). All volatile components were removed under reduced pressure and the residue was dried under vacuum to yield the product as a red solid. Red crystals suitable for single-crystal X-ray diffraction analysis precipitated from a saturated THF/*n*-hexane solution of **Ti3c** after several days. **Yield**: 0.132 g, 0.191 mmol, 71%. ^**1**^ **H NMR** (500 MHz, THF-*d*_8_, 298 K): δ = 2.91 (m, 3 H, N-Me), 6.07 (s, 10 H, Cp-H), 6.71–6.75 (m, 4 H, BPh-H), 6.85–6.89 (m, 8 H, BPh-H), 7.16–7.19 (m, 1 H, Ar–H), 7.29–7.34 (m, 9 H, BPh-H, Ar–H), 7.37–7.44 (m, 1 H, N–H), 7.69–7.77 (m, 1 H, Ar–H), 8.21–8.26 (m, 1 H, Ar–H) ppm. The aldimin-H signal was not found. ^**11**^ **B­{** ^**1**^ **H} NMR** (160 MHz, 298 K, THF-*d*_8_): δ = 6.5 (BPh_4_) ppm. ^**13**^ **C­{** ^**1**^ **H} NMR** (125 MHz, 298 K, THF-*d*_8_): δ = 31.8 (N-Me), 117.3 (Cp-CH), 122.2 (BPh–CH), 126.0 (BPh–CH), 137.4 (BPh–CH), 141.0 (Ar–CH), 154.4 (Ar–CH), 166.0 (Ar–CH) ppm. **IR** (ATR): $\tilde{V}$ = 3298, 3116, 3053, 3000, 2981, 2867, 1581, 1558, 1529, 1490, 1479, 1462, 1428, 1391, 1295, 1268, 1240, 1169, 1157, 1116, 1084, 1055, 1028, 1011, 918, 907, 832, 823, 773, 743, 729, 704, 625, 614, 604, 522 cm^–1^. **Mp**. 177 °C (dec.). **EA**: calcd for C_42_H_39_BN_4_STi: C 73.05, H 5.69, N 8.11. Found: C 73.62, H 5.53, N 7.78.

#### Synthesis of **Ti4c**

Titanocene­(IV)­triflate **Ti2** (300 mg, 0.630 mmol) and 2-pyridinecarboxaldehyde *N*-methylthiosemicarbazone **c** (122 mg, 0.630 mmol) were dissolved in 10 mL of dry THF. The reaction mixture was stirred for 16 h at room temperature to give a yellow suspension. The supernatant was decanted the residue was washed with *n*-hexane (3 × 10 mL). The residue was dried under vacuum to yield the product as a yellow solid. Yellow crystals suitable for single-crystal X-ray diffraction analysis precipitated from a CD_2_Cl_2_ solution of **Ti4c** after several days. **Yield**: 349 mg, 0.521 mmol, 80%. ^**1**^ **H NMR** (500 MHz, D_2_O, 298 K): δ = 3.15 (m, 3 H, N-Me), 6.44 (s, 10 H, Cp-H), 7.78–7.83 (m, 1 H, Ar–H), 7.99–8.02 (m, 1 H, Ar–H), 8.21–8.26 (m, 1 H, Ar–H), 8.61 (s, 1 H, aldimine-H), 8.85–8.89 (m, 1 H, Ar–H) ppm. The N–H signal was not found. ^**13**^ **C­{** ^**1**^ **H} NMR** (125 MHz, 298 K, D_2_O): δ = 31.5 (N-Me), 117.9 (Cp-CH), 127.9 (Ar–CH), 129.3 (Ar–CH), 140.8 (Ar–CH), 147.4 (aldimine-CH), 154.8 (Ar–CH) ppm. ^**15**^ **N NMR** (51 MHz, D_2_O, 298 K): δ = 257.8 (pyridine–N). ^**19**^ **F­{** ^**1**^ **H} NMR** (470 MHz, 298 K, D_2_O): δ = −78.8 ppm. **IR** (ATR): $\tilde{V}$ = 3263, 3116, 3052, 2873, 1629, 1613, 1598, 1562, 1496, 1440, 1397, 1356, 1336, 1312, 1278, 1261, 1248, 1223, 1166, 1152, 1051, 1033, 1024, 940, 914, 849, 775, 748, 725, 633, 575 cm^–1^. **Mp**. 175 °C (dec.). **EA**: calcd for C_20_H_20_F_6_N_4_O_6_S_3_Ti: C 35.83, H 3.01, N 8.36. Found: C 35.85, H 3.06, N 8.30.

### Biological Assays

#### Cell Culture and Sample Preparation

The human colon cancer cell line HCT116 (ATCC, Manassas, VA) was cultured in DMEM (P04–03600, PAN Biotech, Aidenbach, Germany) with 10% FCS (Fetal Calf Serum, Gibco) and 2 mM l-Glutamine (PAN Biotech, Aidenbach, Germany) at 37 °C in 5% CO_2_. Synthesized compounds and Cisplatin (Sigma-Aldrich, St. Louis, MO) were dissolved in Dulbecco’s Phosphate Buffered Saline (DPBS, PAN Biotech, Aidenbach, Germany) to receive a 1 mM solution. 5-Fluorouracil (5-FU; Thermo Fisher Scientific, Waltham, MA) dissolved in DMSO (Dimethyl sulfoxide, Merck, Darmstadt, Germany) was diluted in DPBS accordingly, never exceeding a minimal dilution of 1:1000 for DMSO. DPBS and DMSO dilutions were utilized as vehicle controls. Titanocene Dichloride (Sigma-Aldrich, St. Louis, MO) was dissolved in DMSO to receive a 100 mM solution, which was diluted accordingly. For Titanocene Dichloride a 1000 μM end concentration was included, here the DMSO minimal dilution was exceeded, corresponding controls were used.

#### Cytotoxicity in Cancer Cells

For viability testing, cells were seeded at 20,000 cells per well in a 96 well plate and cultured for 24 h under normal conditions. After washing with DPBS (PAN Biotech, Aidenbach, Germany), the Medium was changed to DMEM without Phenol Red (PAN Biotech, Aidenbach, Germany). After additional 3 h of incubation, the cells were treated with the compounds of interest with increasing concentrations (0.1 to 300 μM) in triplicates for 24 h. Cisplatin, 5-FU and titanocene dichloride were used as positive controls, the respective DMSO concentrations and DPBS were used as negative (vehicle) controls. Cell viability was determined by MTT (3-(4,5-dimetylthiazol-2-yl)-2,5-diphenyltetrazolium bromide (Thermo Fisher Scientific, Waltham, MA)) assay. MTT was dissolved in DPBS at 5 mg/mL and sterile filtered. At the given time point, 10 μL of MTT solution were added to 100 μL of medium and incubated for 2 h at 37 °C. For analysis, 85 μL of supernatant were removed and 100 μL DMSO/EtOH (1:1; Ethanol, Carl Roth, Karlsruhe, Germany) was added to dissolve formed crystals. Following a 10 min incubation period at 37 °C, the plate was shaken for 20 min at room temperature and read directly in a microplate reader at 570 nm (Tecan Infinite M200; Tecan Group, Crailsheim, Germany). Optical density was measured to assess the mitochondrial activity of the treated cells compared to untreated cells and allowing to draw conclusions on cell viability. Cytotoxicity was calculated by the following equation: %_cytotoxicity_ = (100­((control – sample)/control)). IC_50_ values were calculated from cytotoxicity data analyzed by logarithmic nonlinear regression (four parameters) in GraphPad (GraphPad Software Inc., San Diego, CA).

#### Dead Cell Apoptosis Analysis by Flow Cytometry

HCT116 cells were seeded into 24 well plates at 100,000 cells/well and cultured under normal conditions for 24 h. Cells were treated with the two newly synthesized compounds Ti2c and Ti4c, 5-FU and Cisplatin (10 μM) and Staurosprone (0.3 μM) for 48 h. Cells were stained utilizing the Dead Cell Apoptosis Kit with Annexin V (2.5 μL) and Propidium Iodide (1 μL) (Invitrogen, Waltham, MA) and analyzed by flow cytometry (CytoFLEX Flow Cytometer, Beckam Coulter Life Sceinces, Brea, CA).

#### Uptake Analysis

HCT116 cells were seeded at 1 × 10^6^ cells per well in a 6 well plate and incubated for 24 h under normal conditions. Cells were washed with DPBS and new medium was added for analysis. Cells were treated with 100 μM of each test compound at different durations (0, 0.5, 1, 4 h). The medium was aspirated, the cells were washed with DPBS and afterward scraped from the wells with 1 mL DPBS. After centrifugation the cell pellets were dissolved in methanol, filtered and analyzed via mass spectrometry (Finnigan-MAT95 spectrometer, ESI). As references, triflic acid, **c**, **Ti2**, **Ti2c** and **Ti4c** were dissolved in methanol and measured the same way.

#### Statistical Analysis

The analysis of the data was conducted utilizing GraphPad Prism v8.0 software (GraphPad Software Inc., San Diego, CA). Initially, the data underwent Grubb’s test for outliers to ensure the integrity of the data set. Subsequent, the data was subjected to a Shapiro-Wilk test to ascertain its normal distribution. Thereafter, the MTT data was analyzed by One-way ANOVA with Tukey′s multiple comparisons test to determine statistical significance. Flow cytometry data was analyzed by Two-way ANOVA after testing for normal distribution by Shapiro-Wilk test.

#### Gel Electrophoresis Analysis

Complexes **Ti2c** and **Ti4c** and their respective precursors (TSCN **c** and **Ti2**) were dissolved in Milli-Q water and were incubated at 37 °C with 0.0625 μg μL^–1^ pBR322 plasmid DNA, at different concentrations expressed as *r*_i_ = complex: DNA (base pair) ratio. The *r*_i_ used is 0.01, 0.05, 0.10, 0.20 and to 0.25 in a total volume of 20 μL. After an incubation period of 24 h, the mobility of the treated pBR322 samples was analyzed by gel electrophoresis at 70 V in Tris/acetate/EDTA buffer. A control of pBR322 was also incubated, and one load of 1 kb ladder was loaded in lane 1 of each gel. The gels were stained with ethidium bromide aqueous solution and DNA bands were visualized with a UV-transilluminator UVITEC Cambridge UVIDOC HD2 instrument and Nikon Elipse camera.

## Supplementary Material



## Notes

NMR, IR, EPR, UV/vis, mass spectra and crystallographic data (PDF)
