# Supplementary material for: Water-Soluble, Titanocene-Based Prodrugs with Thiosemicarbazones as Cytotoxic Agents
Source: ACS Omega. 2025 Sep 3;10(36):41677–87. doi: 10.1021/acsomega.5c05481 (PMC12444608; doi:10.1021/acsomega.5c05481)
Supplement: Supplementary file 1 [file ao5c05481_si_001.pdf]

Supporting Information

Water-soluble, Titanocene-based Prodrugs with Thiosemicarbazones as Cytotoxic Agents

Kevin Schwitalla*†[a], Marie-Carlotta Müller†[b], David Fabra[c], Marc Schmidtmann[a], Ulrike Meyer[b], Ana I. Matesanz[c], Adoracion G. Quiroga*[c], Bernhard Rauch*[b], and Rüdiger Beckhaus[a]

[a] Chemistry Department, Carl von Ossietzky University of Oldenburg, 26111 Oldenburg, Germany

[b] Department für Humanmedizin, Carl von Ossietzky University of Oldenburg, 26111 Oldenburg, Germany

[c] Department of Inorganic Chemistry, Universidad Autonoma de Madrid, Cantoblanco, 28049 Madrid, Spain

†: authors contributed equally to this work.

TABLE OF CONTENTS

EPR spectra........ 2

UV/VIS spectra........ 4

NMR Spectra of water-solubility and stability studies 7

Mass spectra........ 10

NMR spectra of complexes 19

Crystallographic data........ 25

IR spectra........ 32

Apoptosis studies........ 36

References........ 37

EPR Spectra

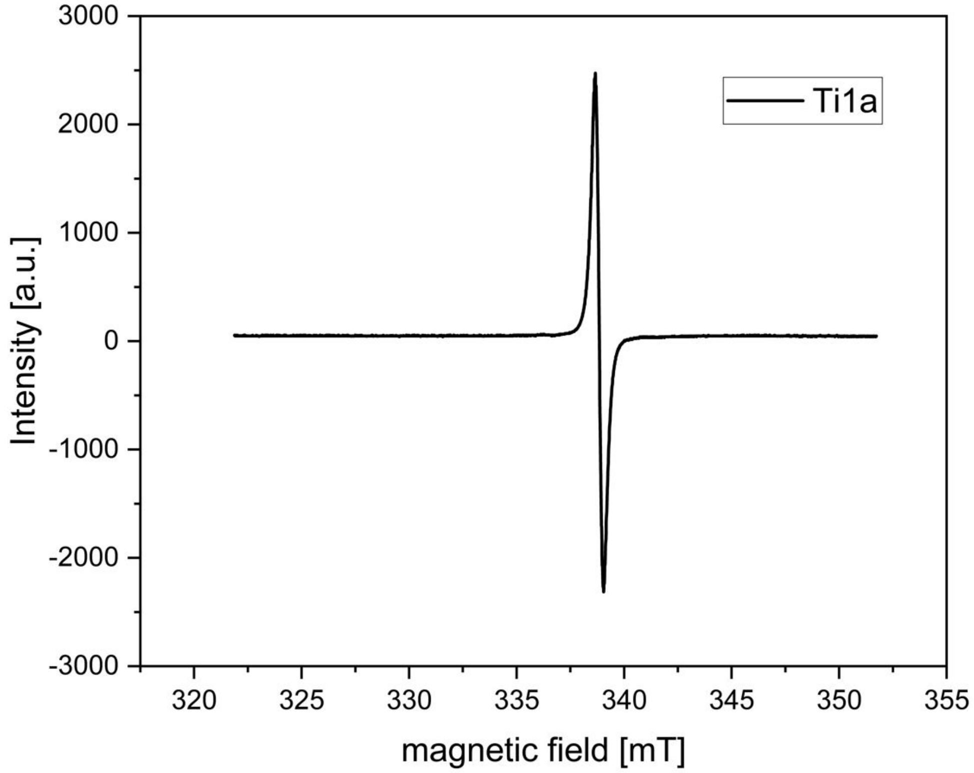

Figure S1: EPR spectrum of complex **Ti1a** in toluene at room temperature (g = 1.980).

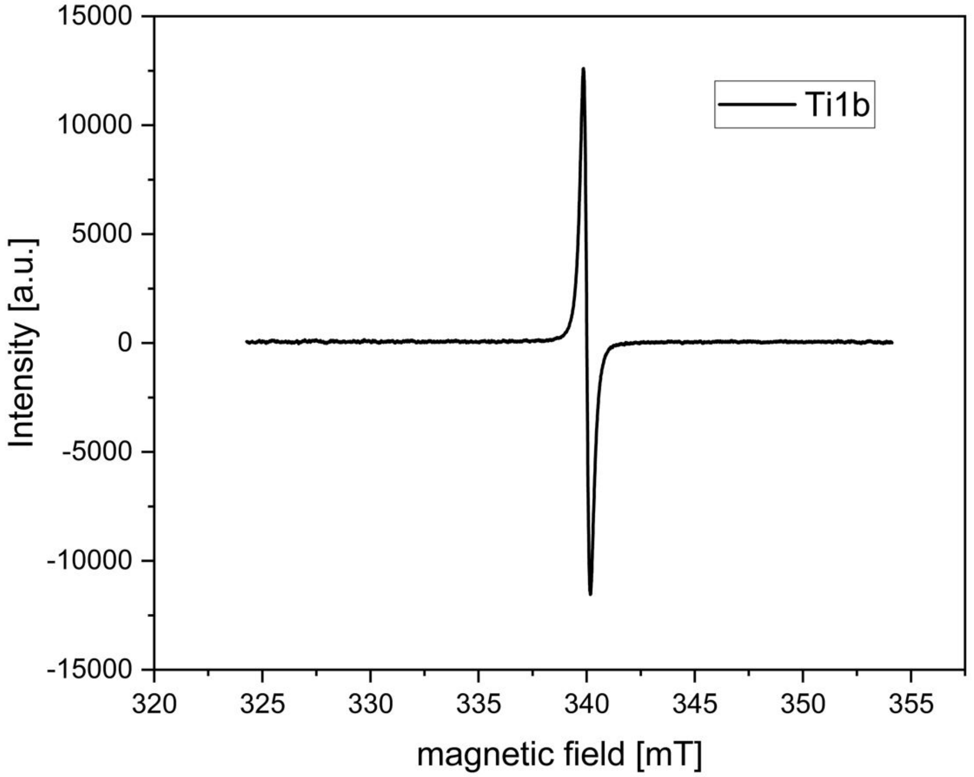

Figure S2: EPR spectrum of complex **Ti1b** in benzene at room temperature (g = 1.983).

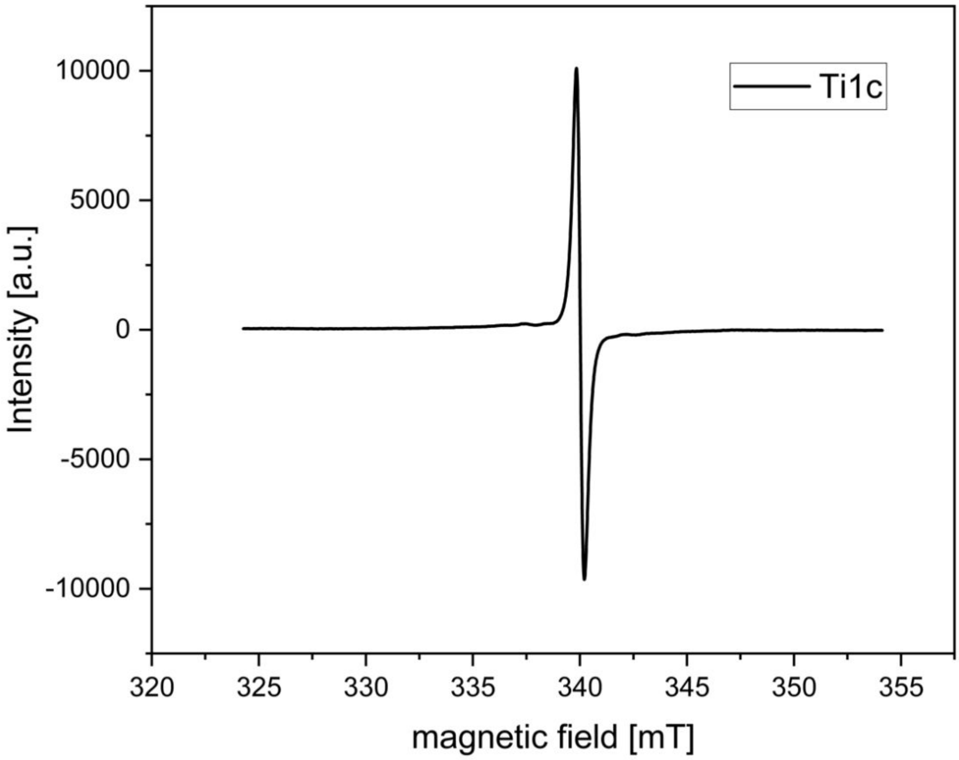

Figure S3: EPR spectrum of complex **Ti1c** in benzene at room temperature (g = 1.983).

UV/Vis spectra

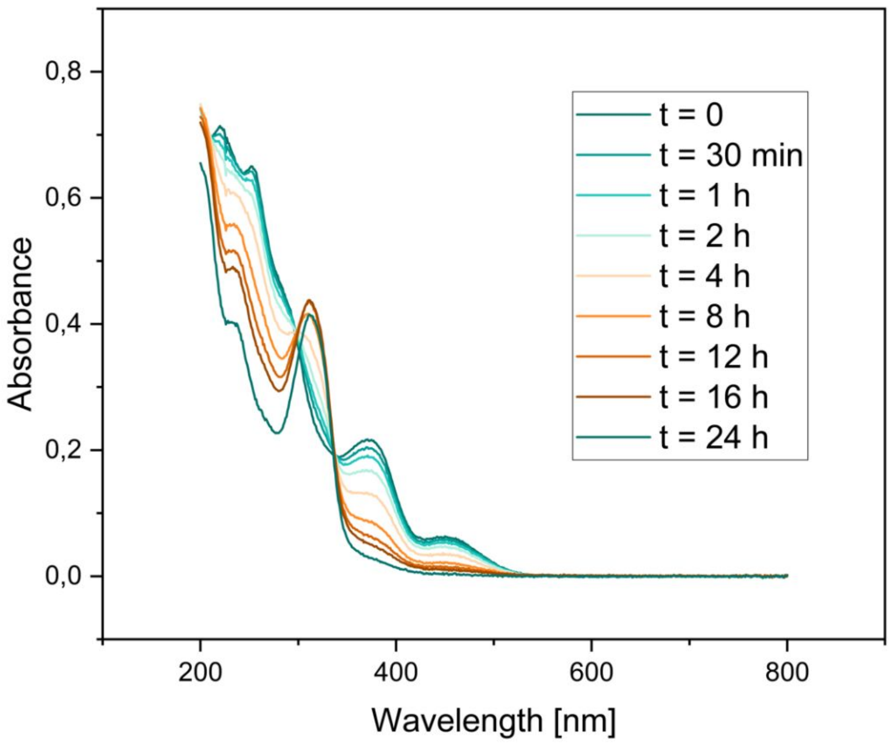

Figure S4: Stacked UV/Vis spectra of **Ti2c** in deionized water (15 µM) at room temperature depending on time.

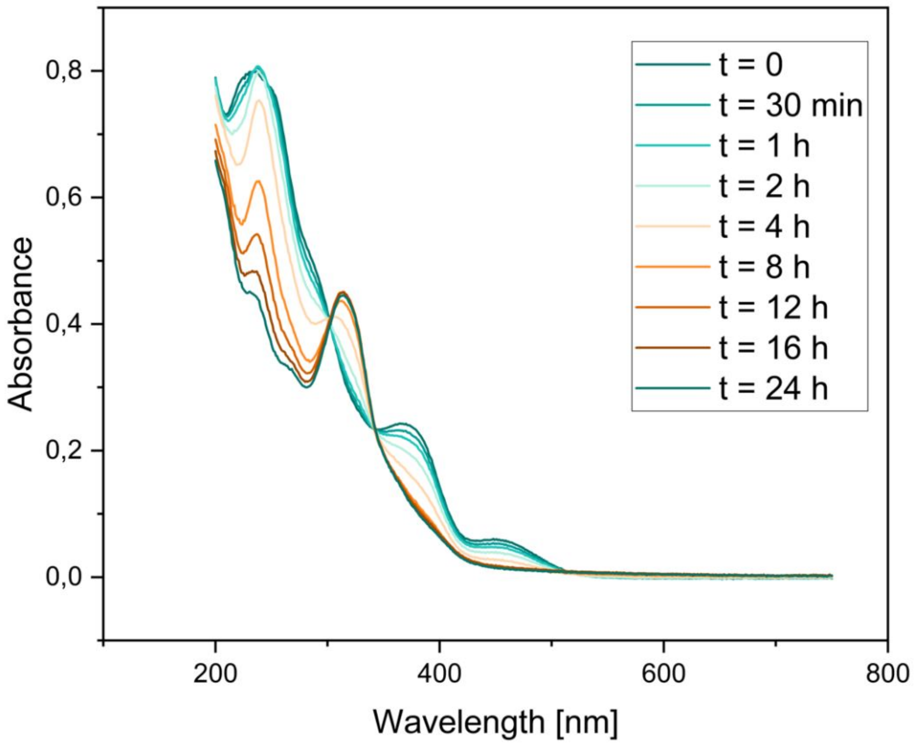

Figure S5: Stacked UV/Vis spectra of **Ti4c** in deionized water (15 µM) at room temperature depending on time.

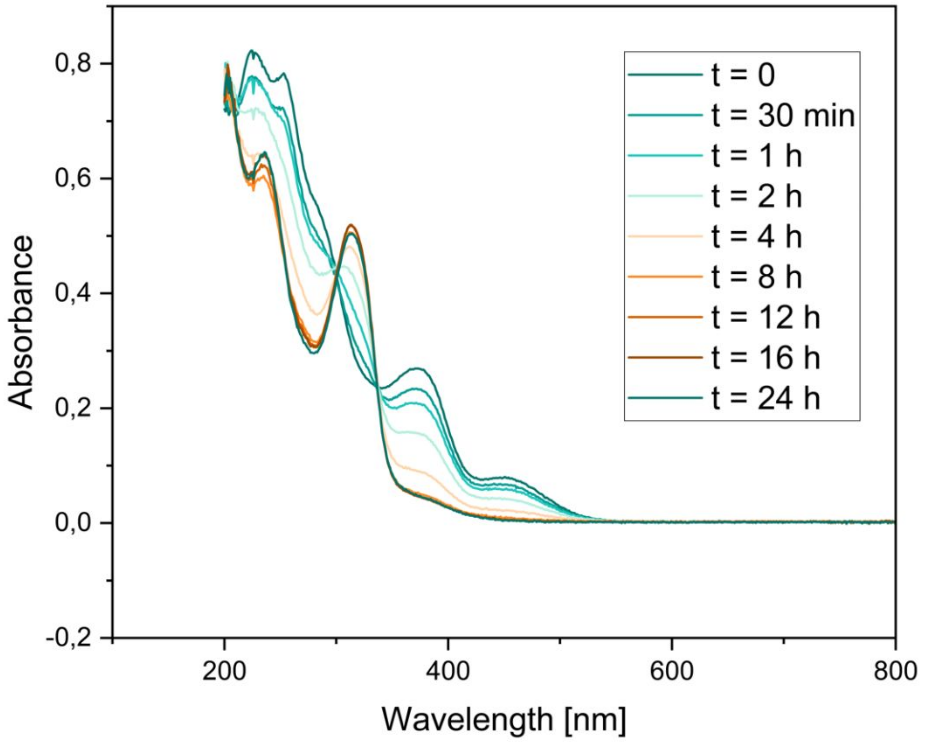

Figure S6: Stacked UV/Vis spectra of **Ti2c** in Tris-HCl buffer (15 µM, pH = 7.4) at room temperature depending on time.

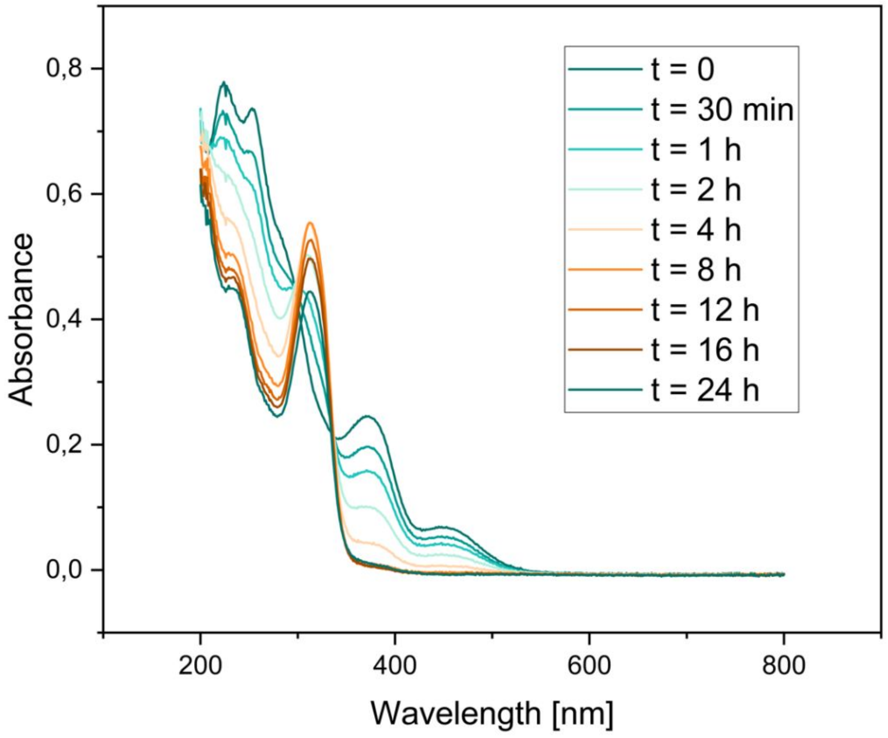

Figure S7: Stacked UV/Vis spectra of **Ti4c** in Tris-HCl buffer (15 µM, pH = 7.4) at room temperature depending on time.

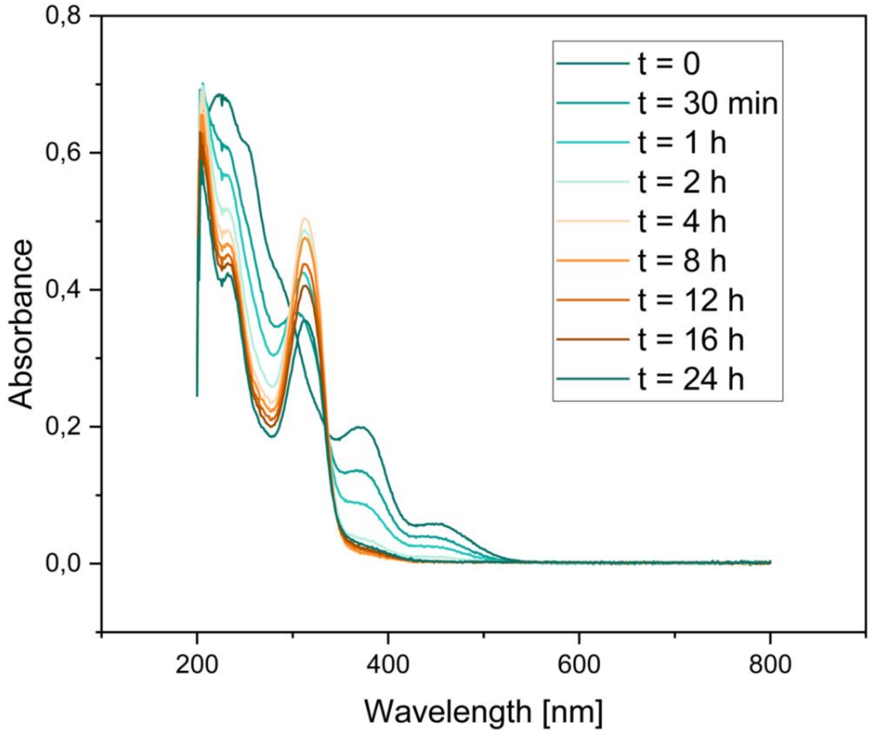

Figure S8: Stacked UV/Vis spectra of **Ti2c** in DPBS buffer (15 µM, pH = 7.2) at room temperature depending on time.

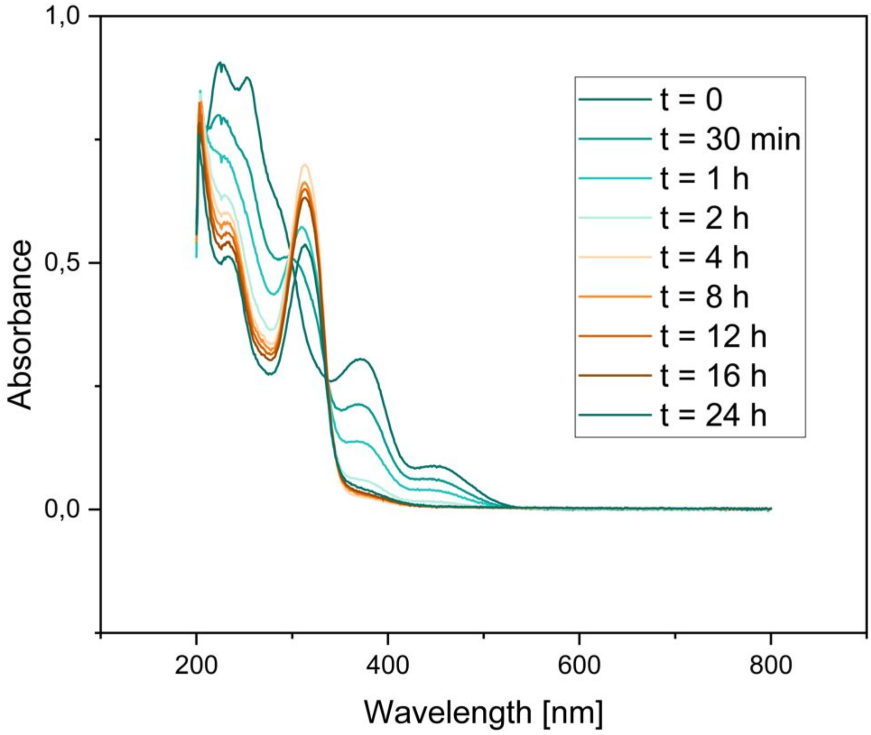

Figure S9: Stacked UV/Vis spectra of **Ti4c** in DPBS buffer (15 µM, pH = 7.2) at room temperature depending on time.

NMR Spectra of water-solubility and stability studies

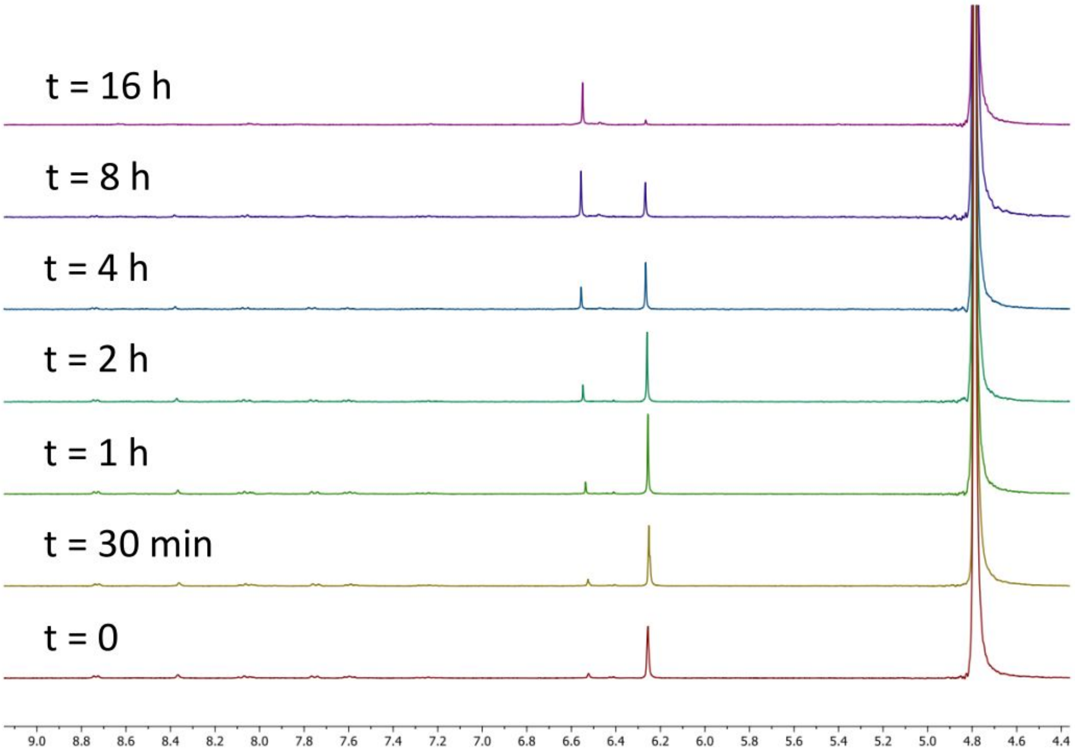

Figure S10: Stacked ^{1}H NMR spectra (300 MHz, D_2O, 298 K) of **Ti2c** over time. 6.2 ppm: Cp signal of **Ti2c**. 6.5 ppm: Cp signal of hydrolized product.

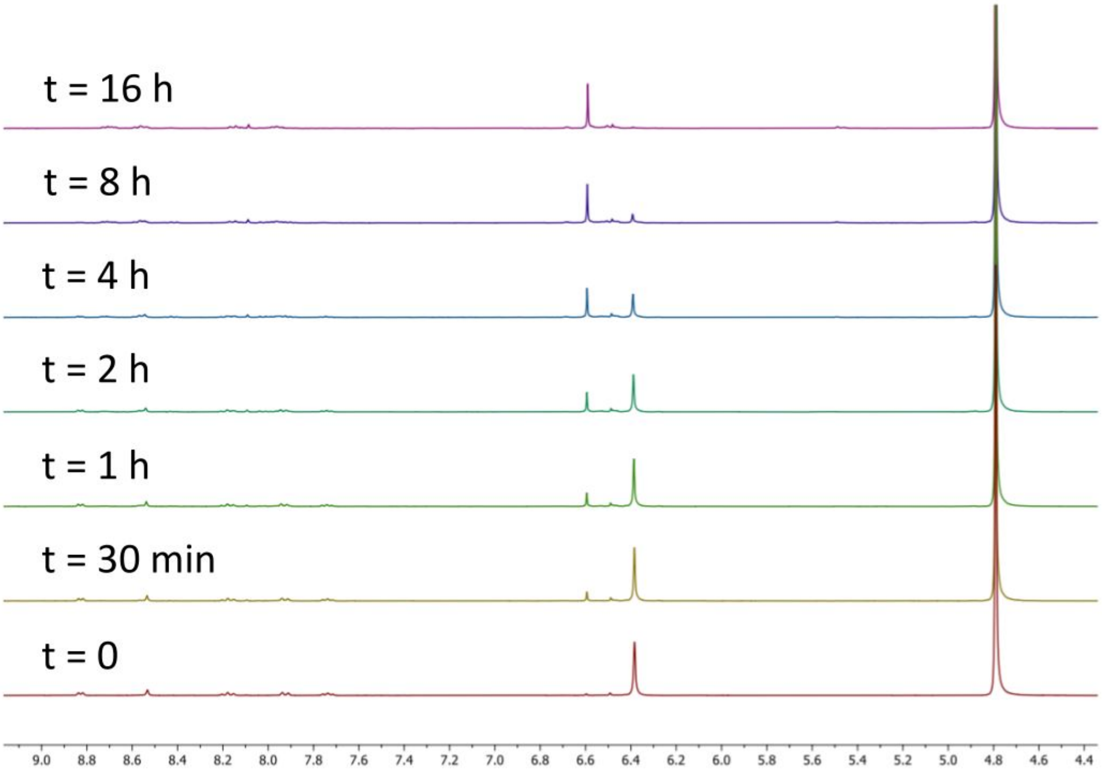

Figure S11: Stacked ^{1}H NMR spectra (300 MHz, D_2O, 298 K) of **Ti4c** over time. 6.4 ppm: Cp signal of **Ti4c**. 6.6 ppm: Cp signal of hydrolized product $[Cp_2Ti(D_2O)_2](OTf)_2$.

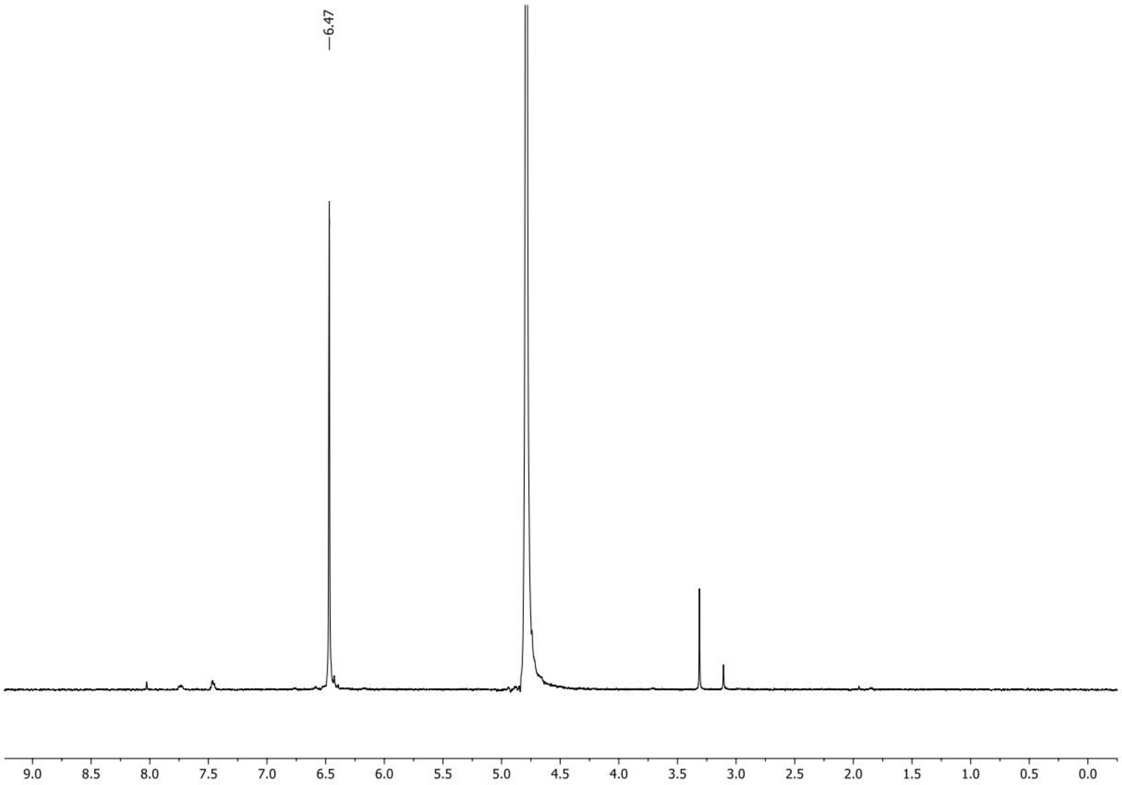

Figure S12: ^{1}H NMR spectrum (300 MHz, D_2O, 298 K) of **Ti2a**. 6.47 ppm: Cp signal of hydrolized product [$Cp_2Ti(D_2O)$-μO-$Cp_2Ti(D_2O)$]$(OTf)_2$.

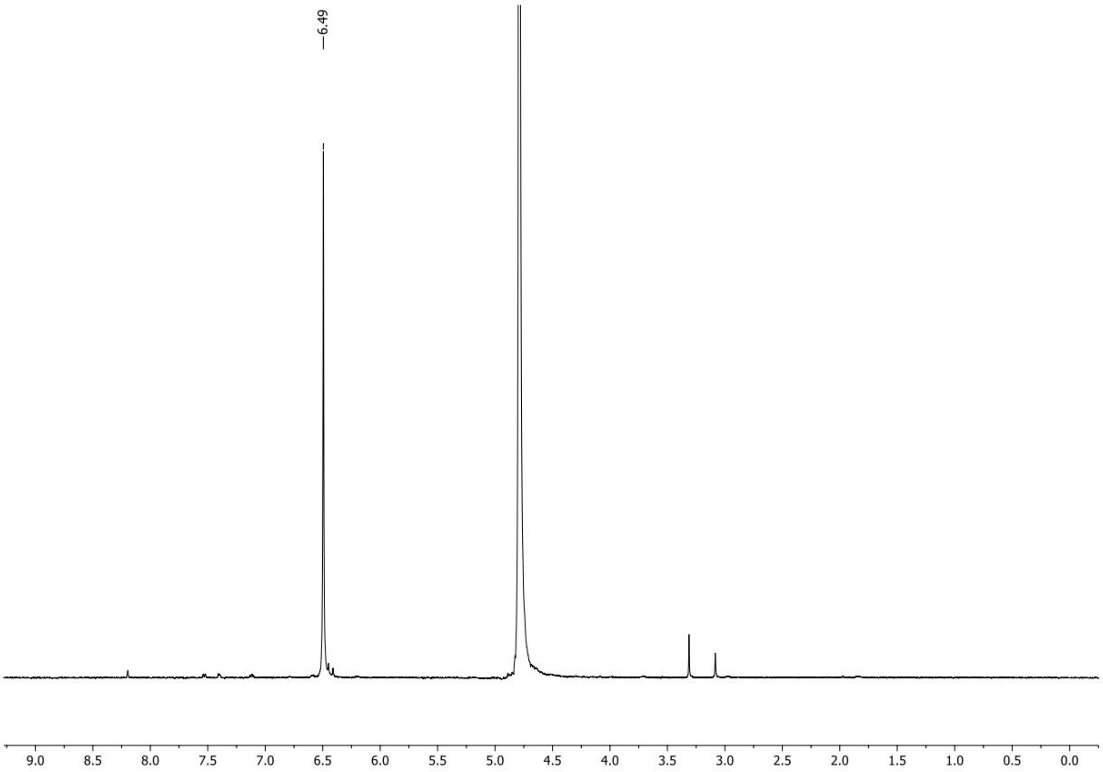

Figure S13 ^{1}H NMR spectrum (300 MHz, D_2O, 298 K) of **Ti2b**. 6.47 ppm: Cp signal of hydrolized product [$Cp_2Ti(D_2O)$-μO-$Cp_2Ti(D_2O)$]$(OTf)_2$.

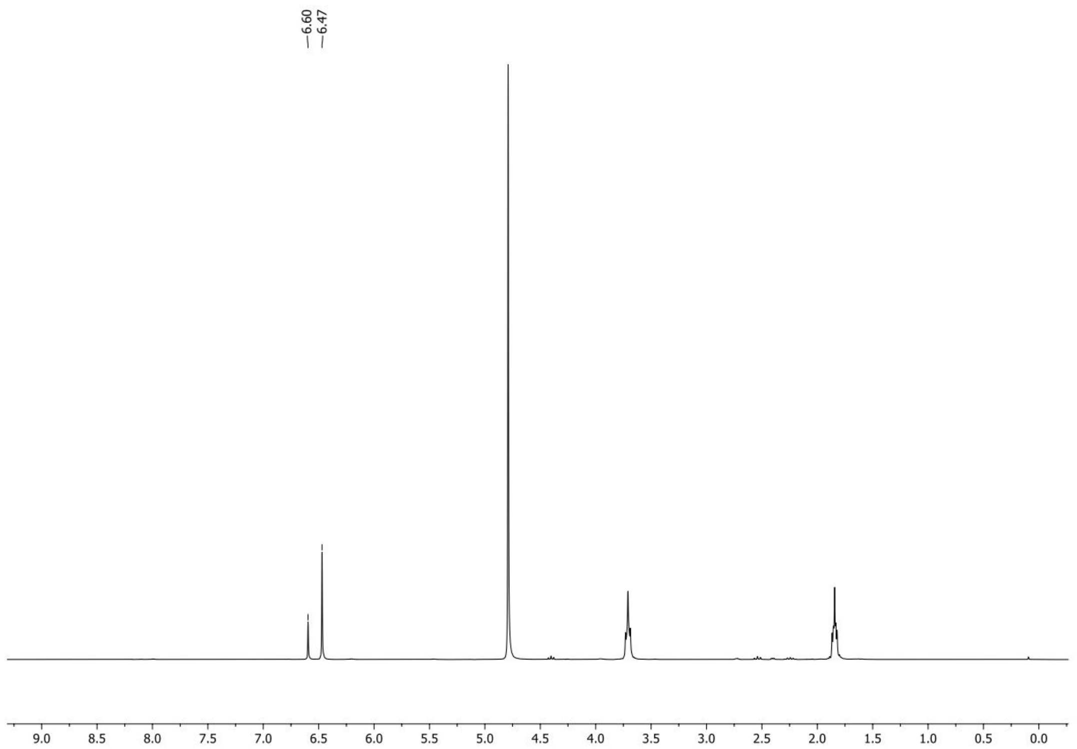

Figure S14: ^{1}H NMR spectrum (300 MHz, D_2O, 298 K) of [$Cp_2Ti(OTf)$-µO-$Cp_2Ti(OTf)$]. 6.47 ppm: Cp signal of hydrolized product [$Cp_2Ti(D_2O)$-μO-$Cp_2Ti(D_2O)$]$(OTf)_2$, 6.60 ppm: Cp signal of by-product [$Cp_2Ti(D_2O)_2$]$(OTf)_2$.

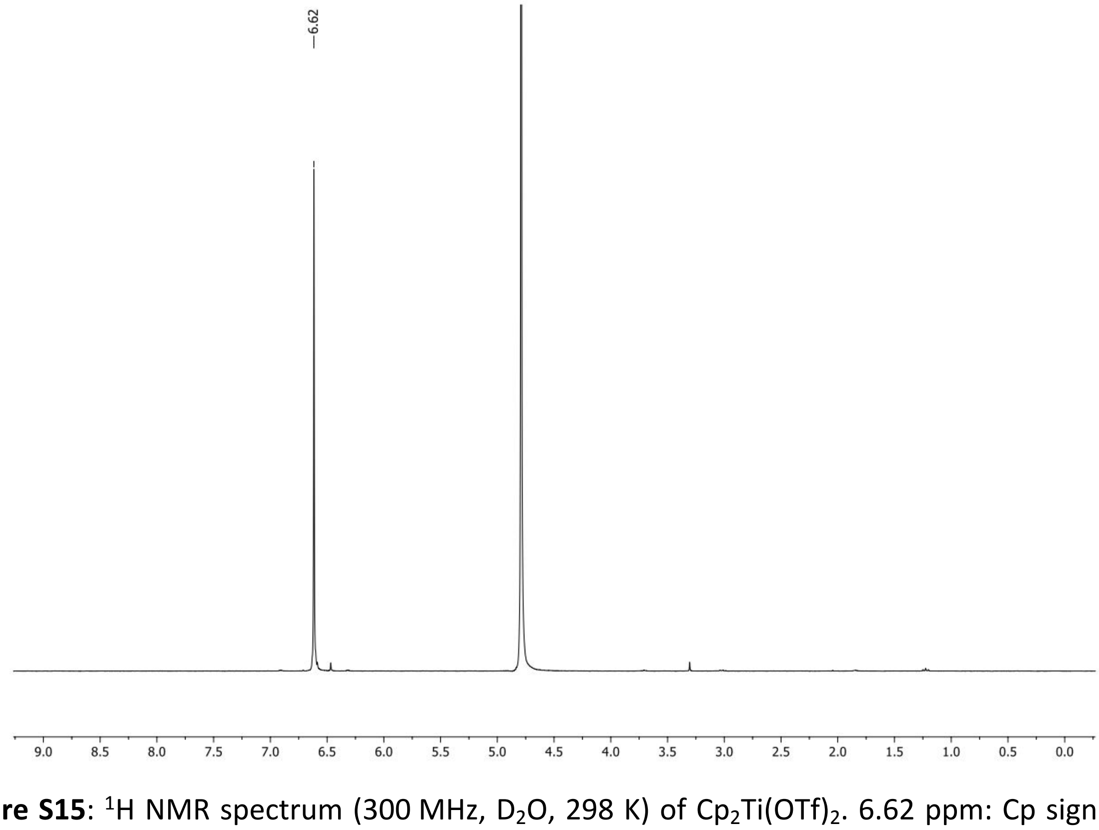

Figure S15: ^{1}H NMR spectrum (300 MHz, D_2O, 298 K) of $Cp_2Ti(OTf)_2$. 6.62 ppm: Cp signal of hydrolized product [$Cp_2Ti(D_2O)_2$]$(OTf)_2$.

Mass spectra

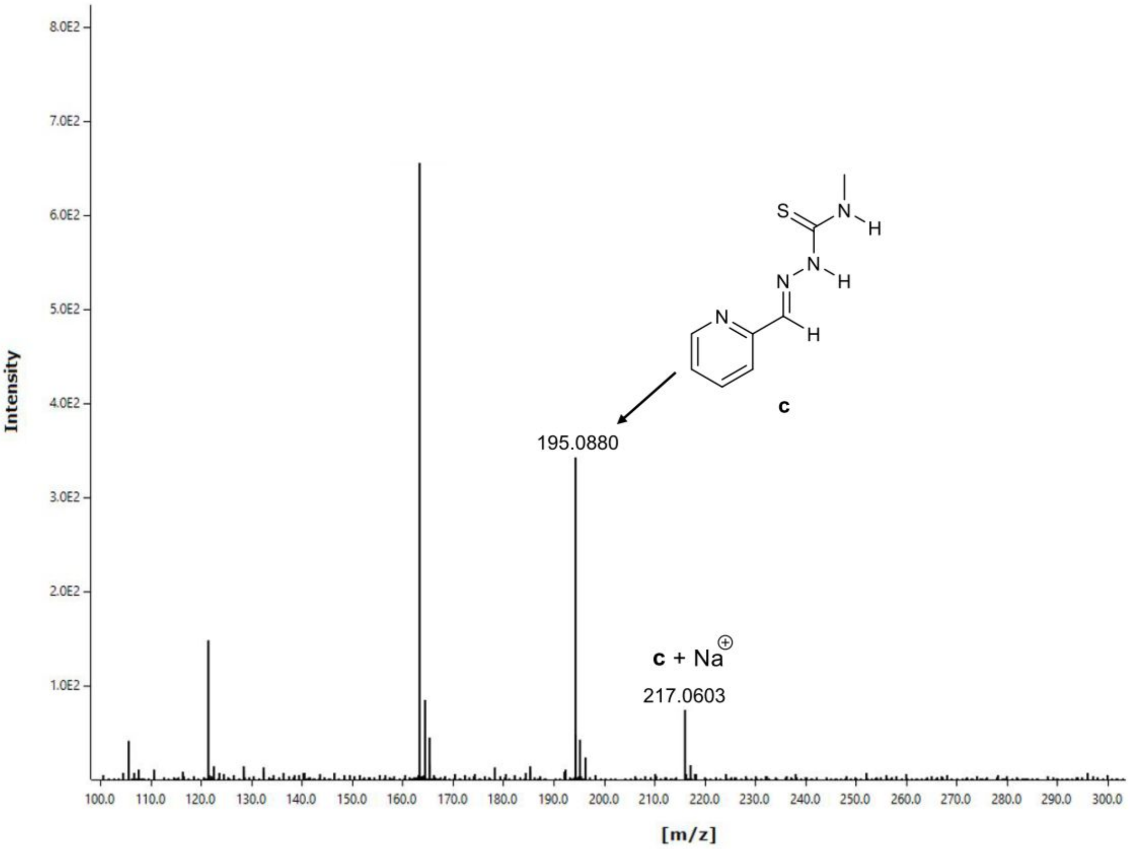

Figure S16: Mass spectrum (ESI, positive mode, methanol) of **c**.

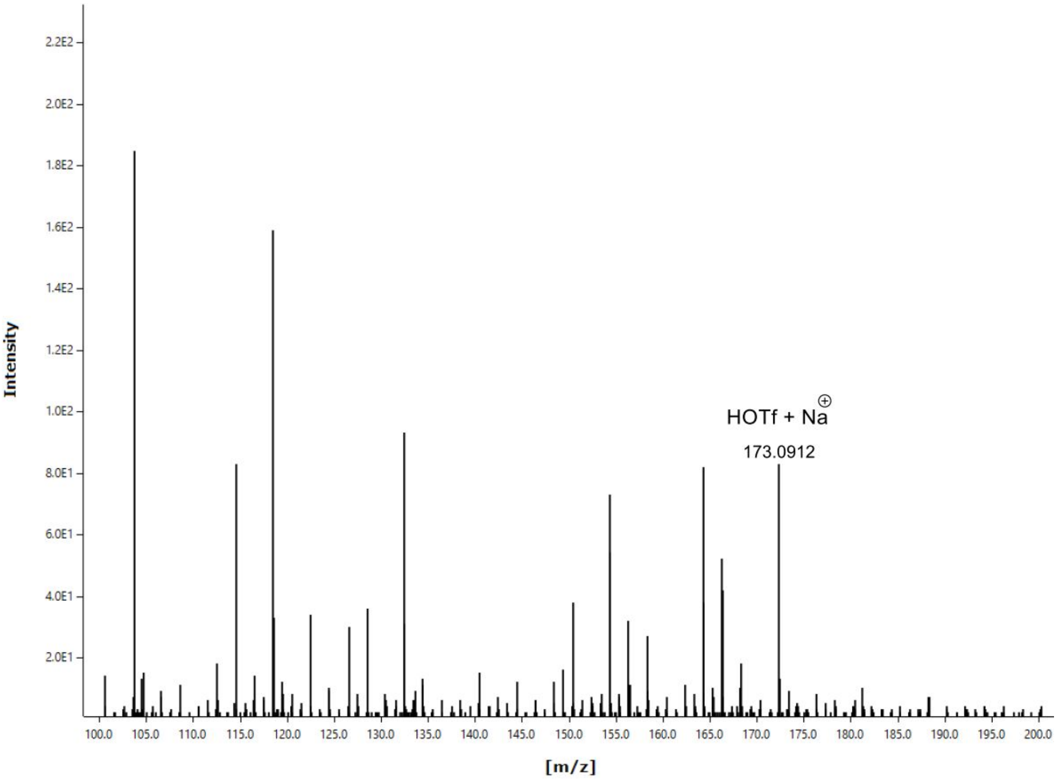

Figure S17: Mass spectrum (ESI, positive mode, methanol) of triflic acid.

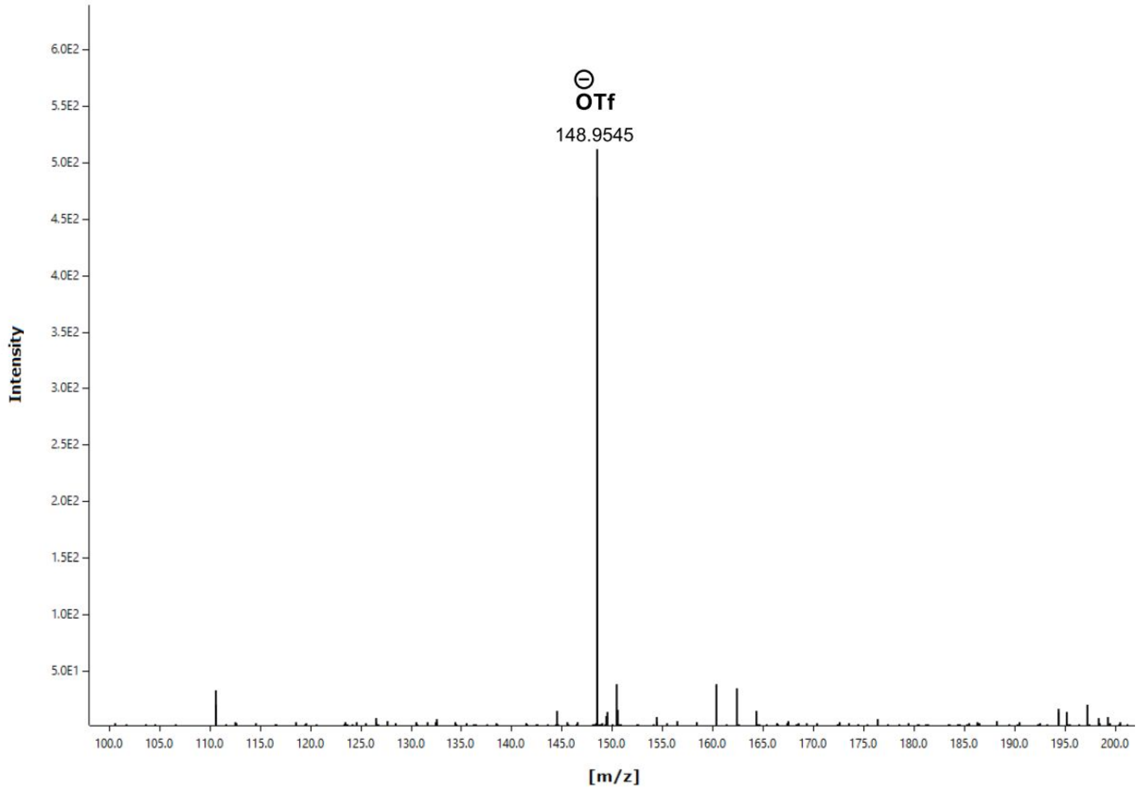

Figure S18: Mass spectrum (ESI, negative mode, methanol) of triflic acid.

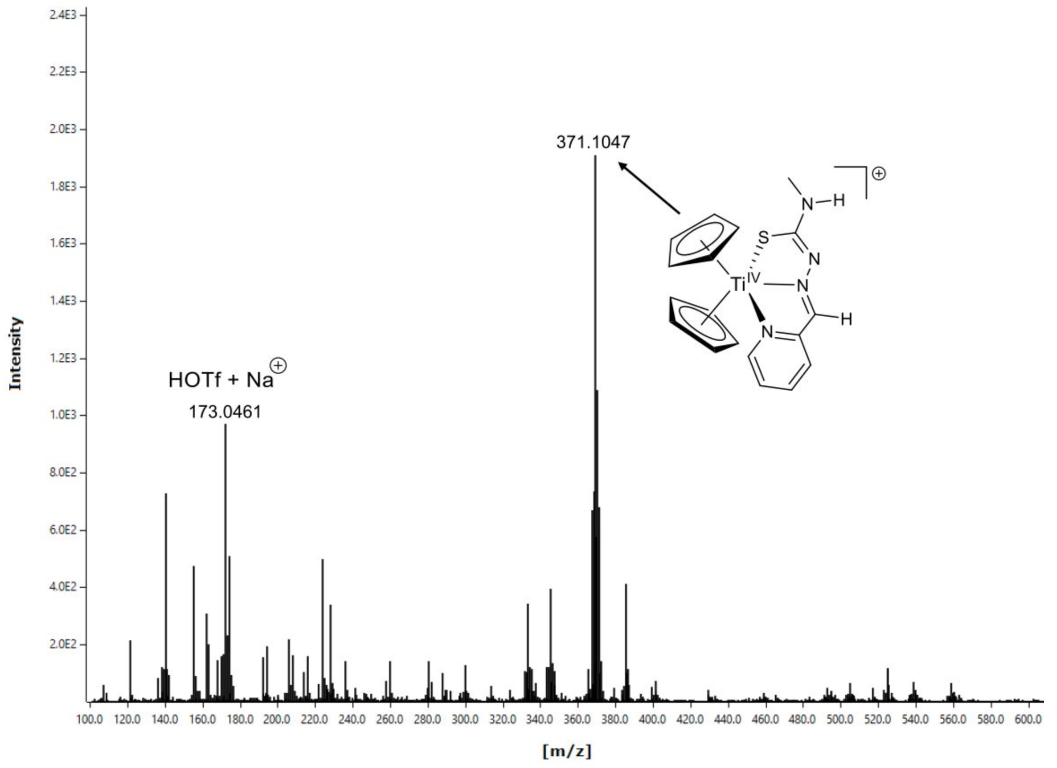

Figure S19: Mass spectrum (ESI, positive mode, methanol) of **Ti2c**.

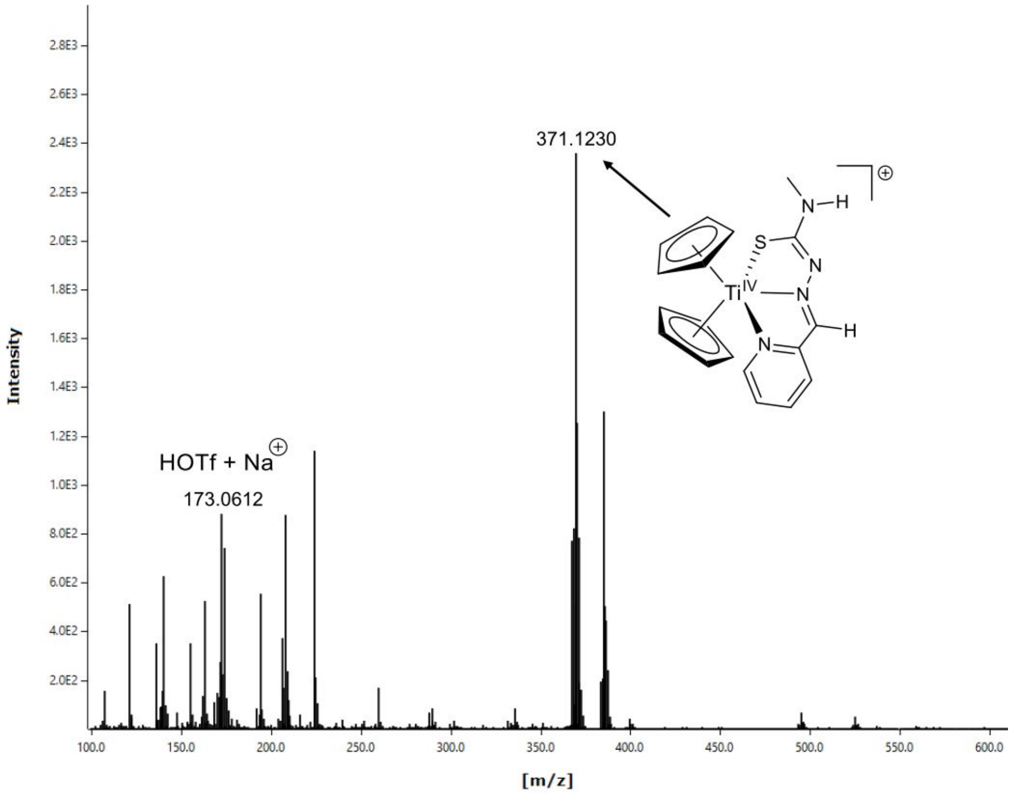

Figure S20: Mass spectrum (ESI, positive mode, methanol) of **Ti4c**.

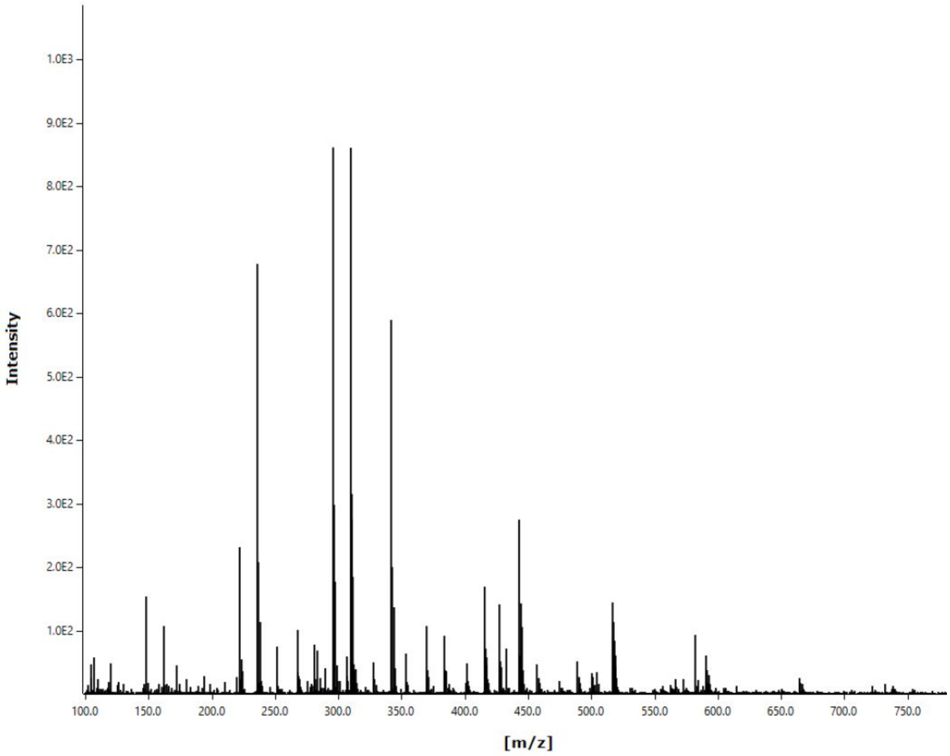

Figure S21: Control mass spectrum (ESI, positive mode, methanol) of isolated cells, that were treated with **Ti2c** and immediately isolated (0 h).

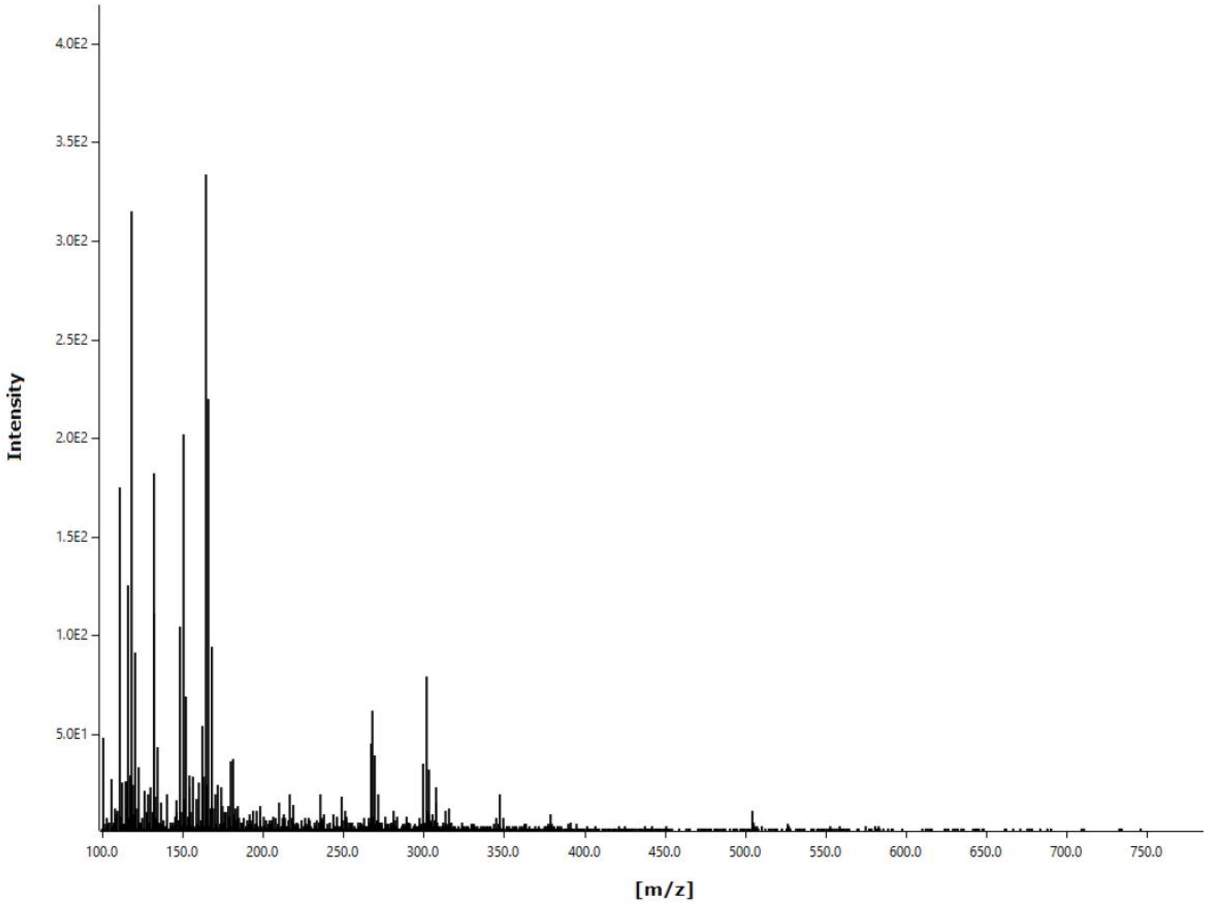

Figure S22: Mass spectrum (ESI, positive mode, methanol) of isolated cells, that were treated with **Ti2c** for 0.5 h.

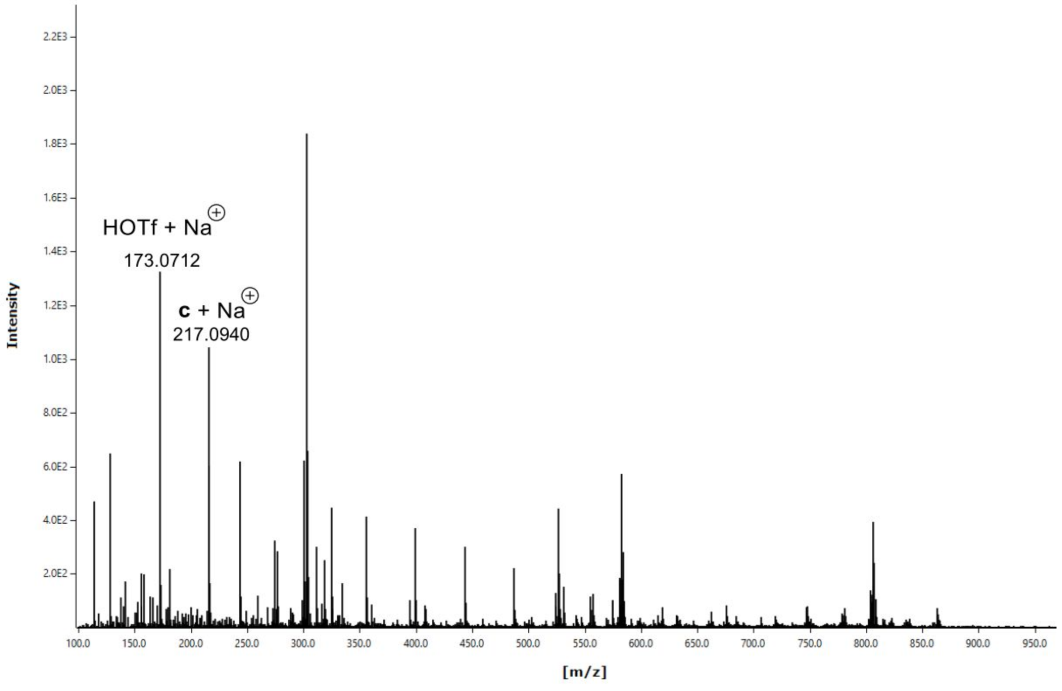

Figure S23: Mass spectrum (ESI, positive mode, methanol) of isolated cells, that were treated with **Ti2c** for 1 h.

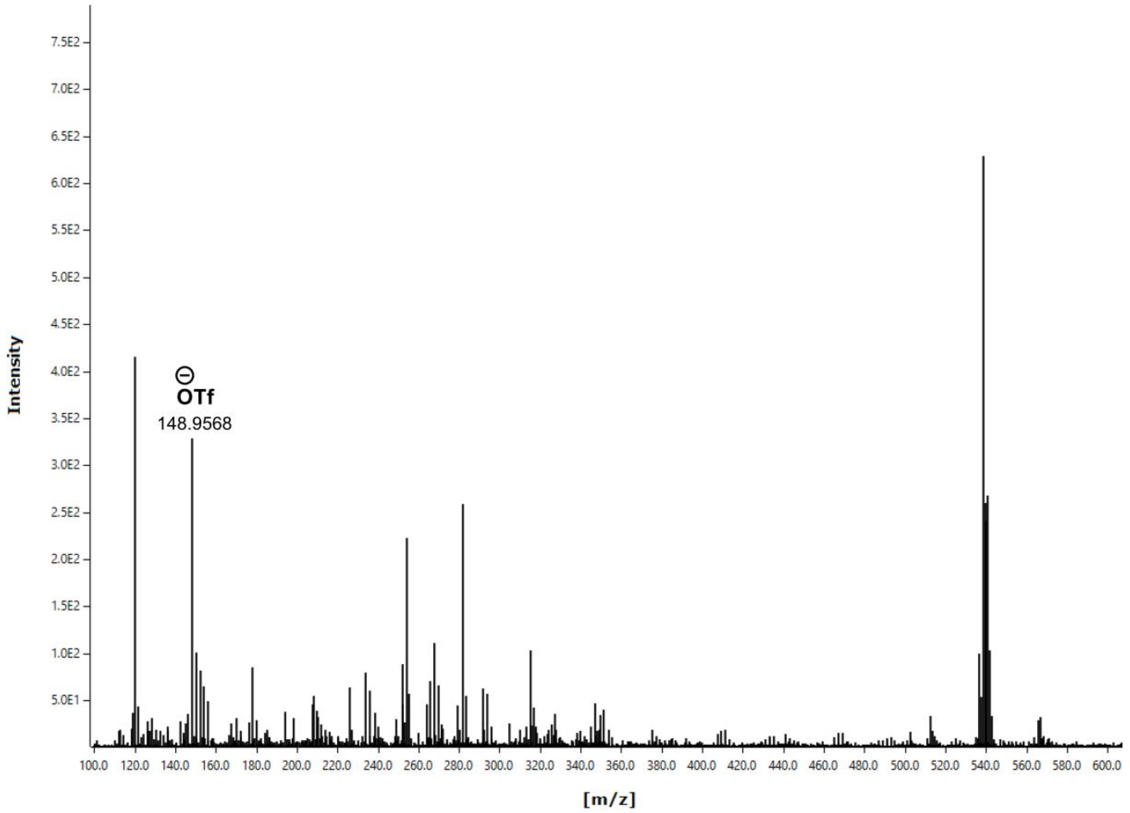

Figure S24: Mass spectrum (ESI, negative mode, methanol) of isolated cells, that were treated with **Ti2c** for 1 h.

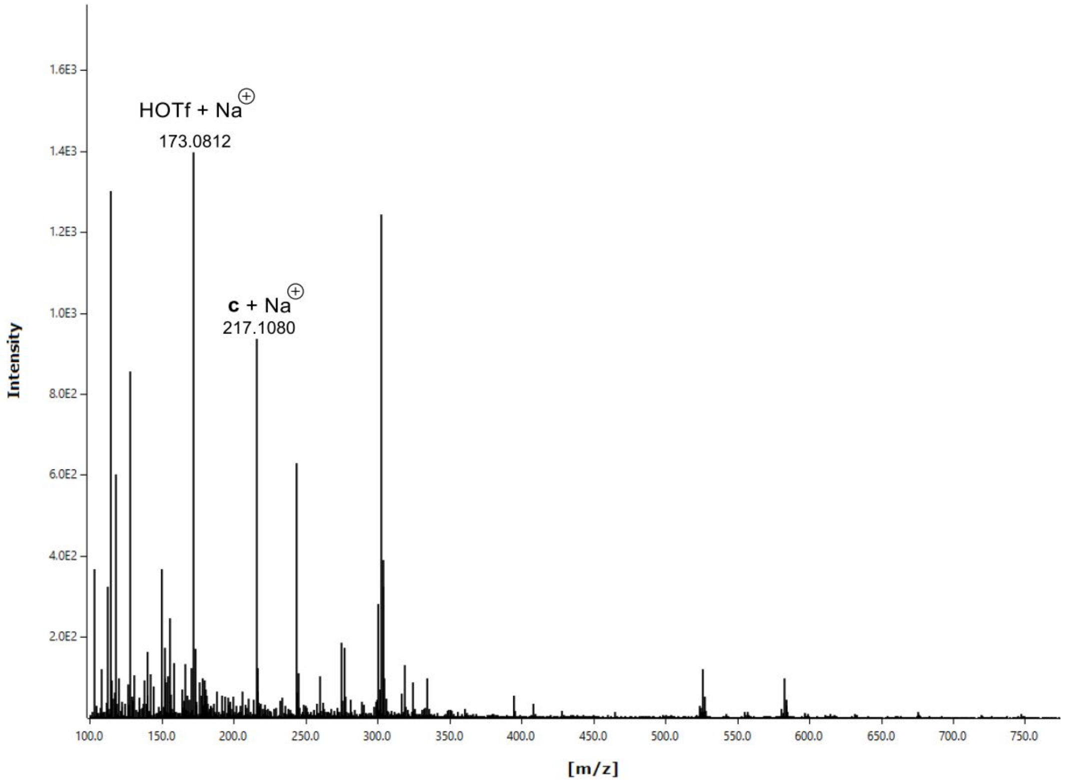

Figure S25: Mass spectrum (ESI, positive mode, methanol) of isolated cells, that were treated with **Ti2c** for 4 h.

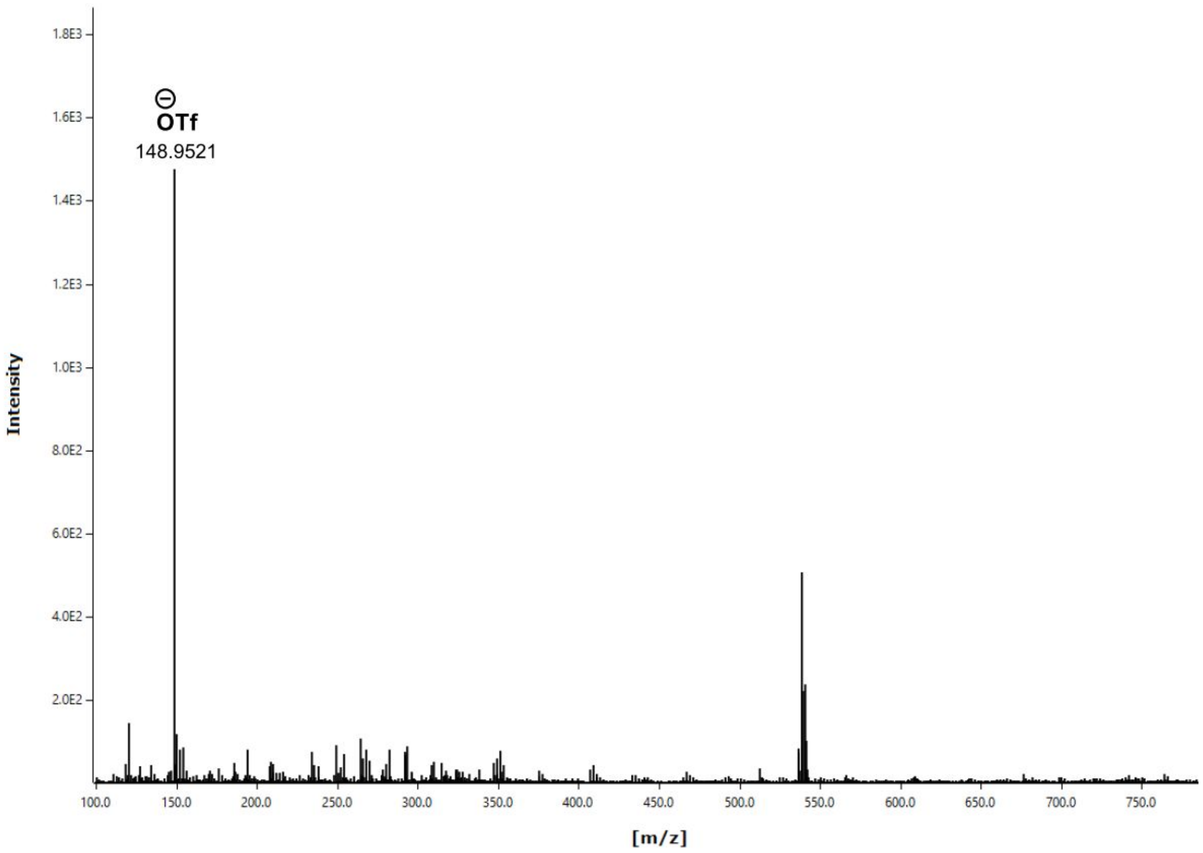

Figure S26: Mass spectrum (ESI, negative mode, methanol) of isolated cells, that were treated with **Ti2c** for 4 h.

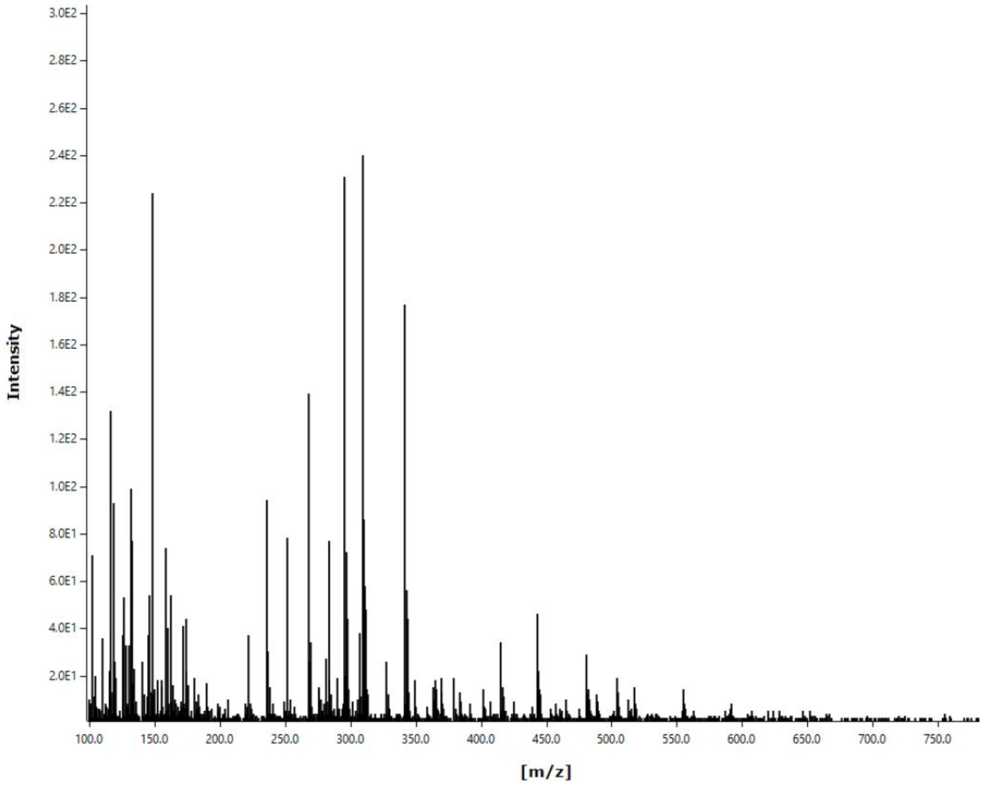

Figure S27: Control mass spectrum (ESI, positive mode, methanol) of isolated cells, that were treated with **Ti4c** and immediately isolated (0 h).

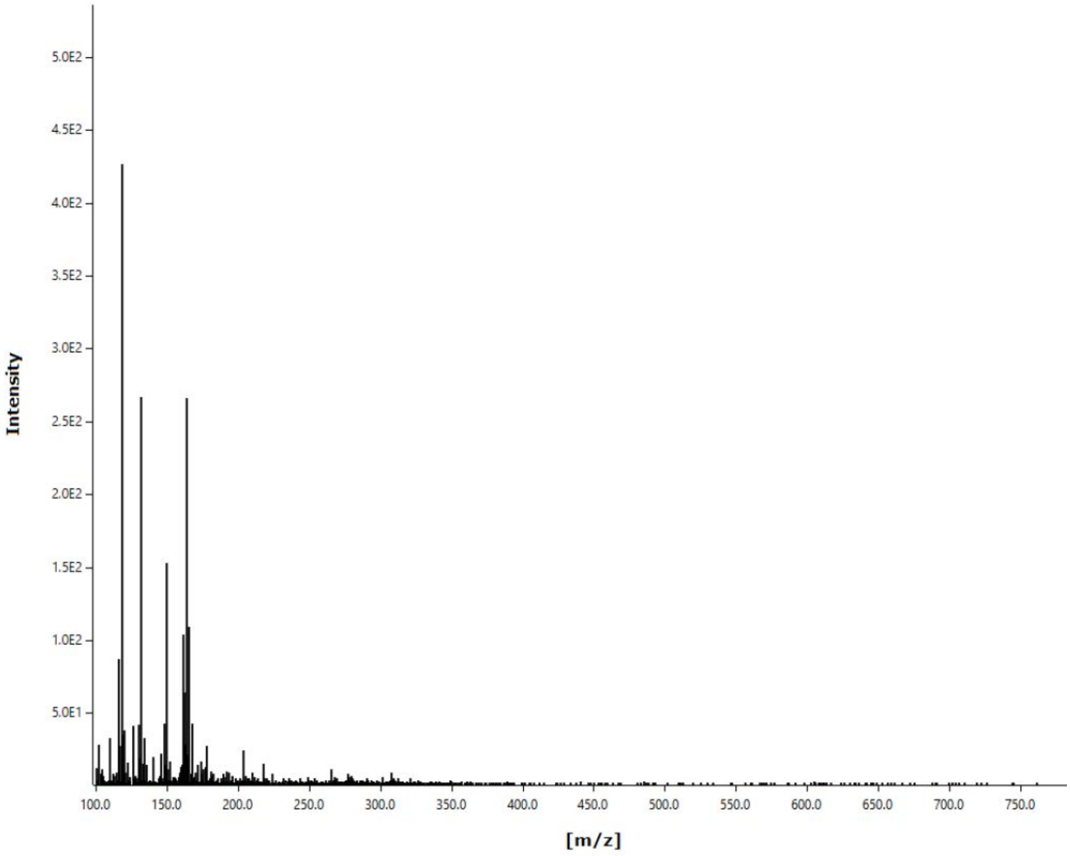

Figure S28: Mass spectrum (ESI, positive mode, methanol) of isolated cells, that were treated with **Ti4c** for 0.5 h.

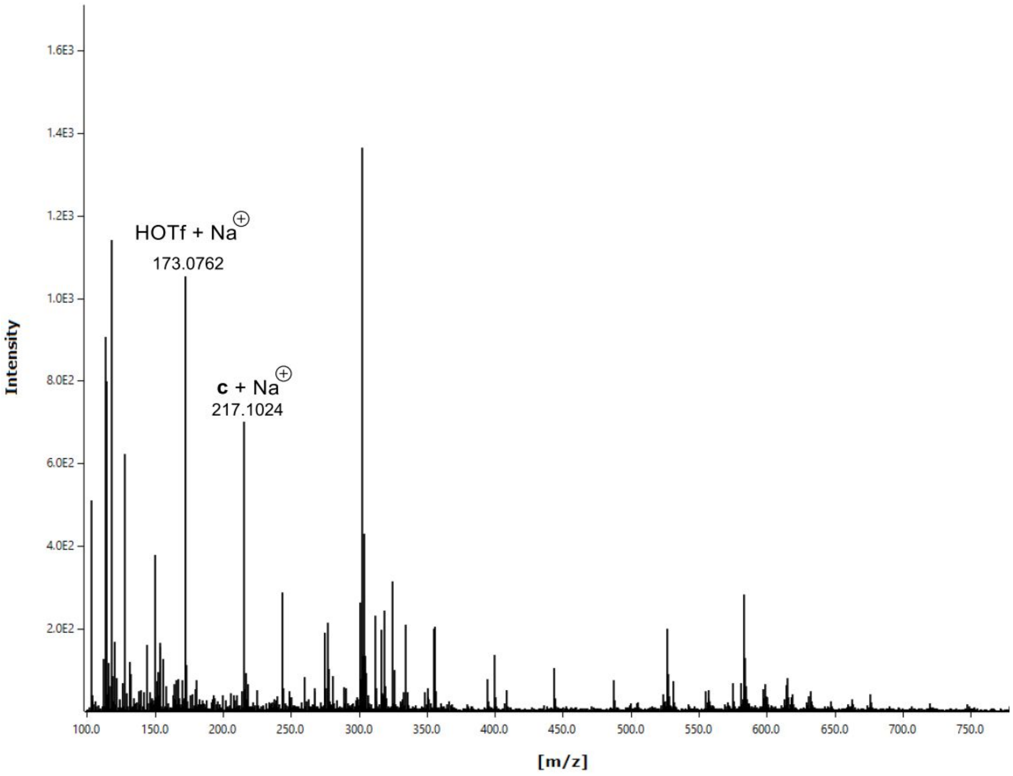

Figure S29: Mass spectrum (ESI, positive mode, methanol) of isolated cells, that were treated with **Ti4c** for 1 h.

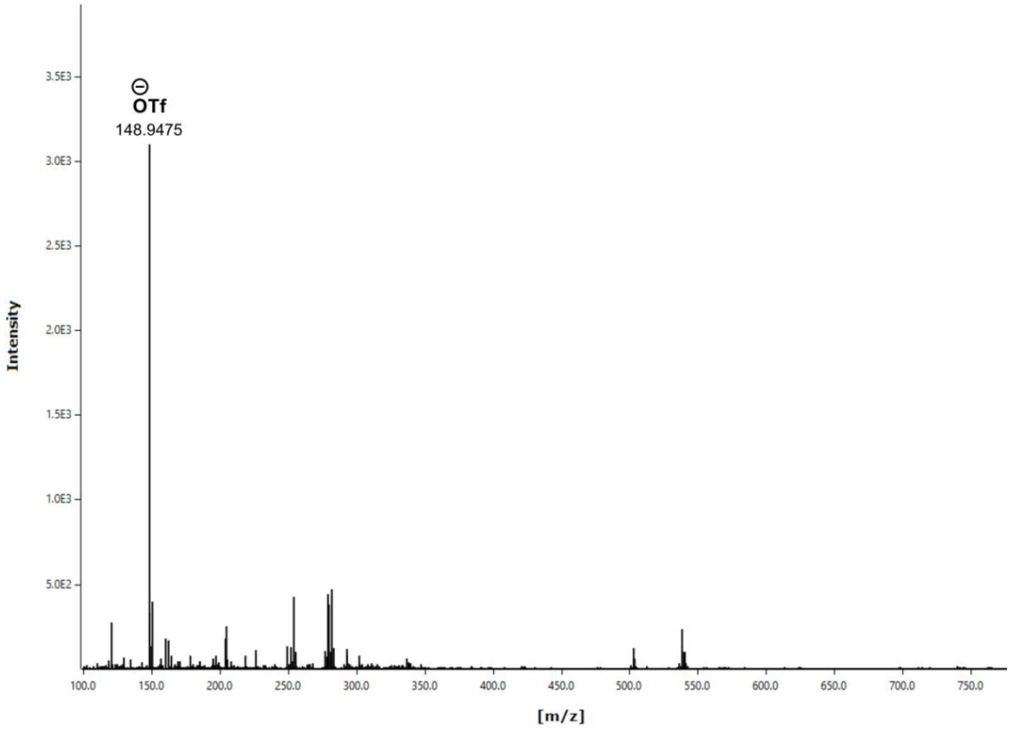

Figure S30: Mass spectrum (ESI, negative mode, methanol) of isolated cells, that were treated with **Ti4c** for 1 h.

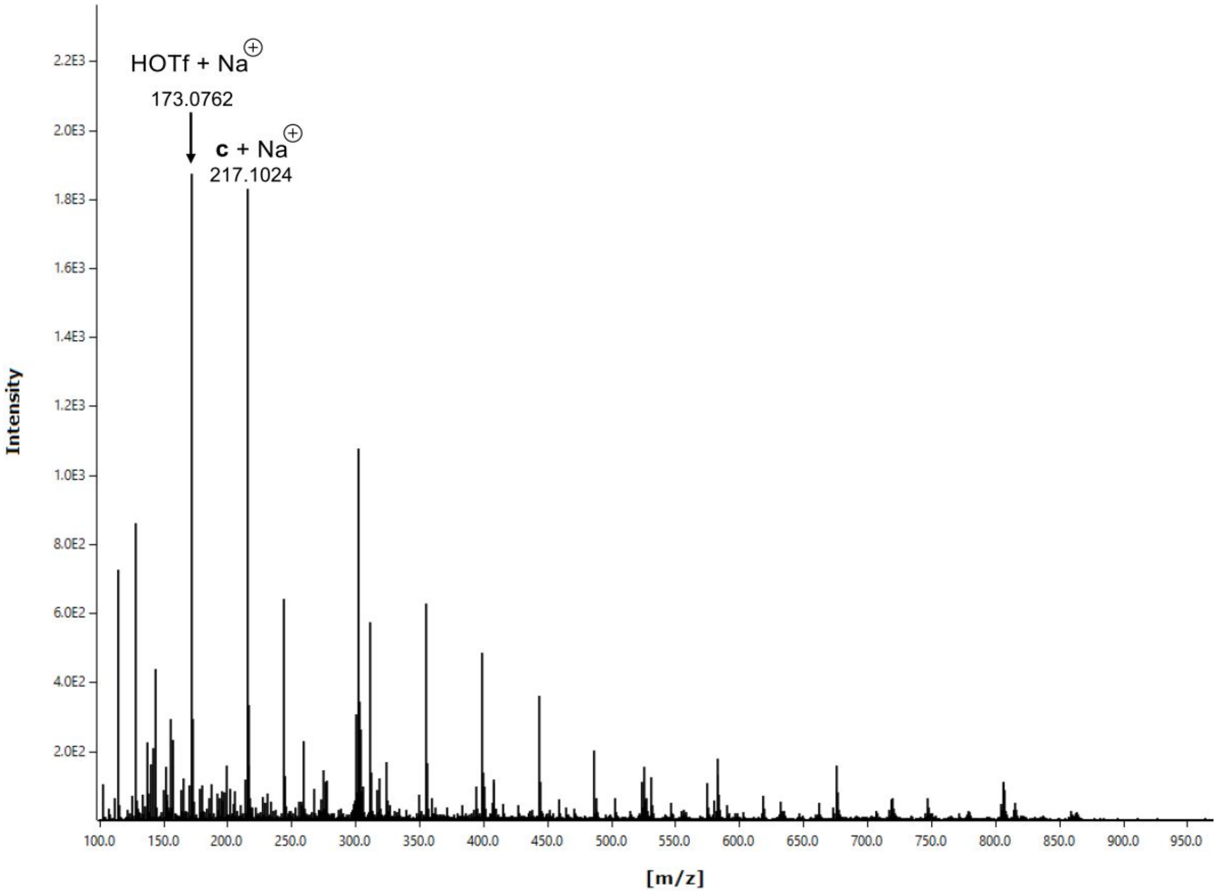

Figure S31: Mass spectrum (ESI, positive mode, methanol) of isolated cells, that were treated with **Ti4c** for 4 h.

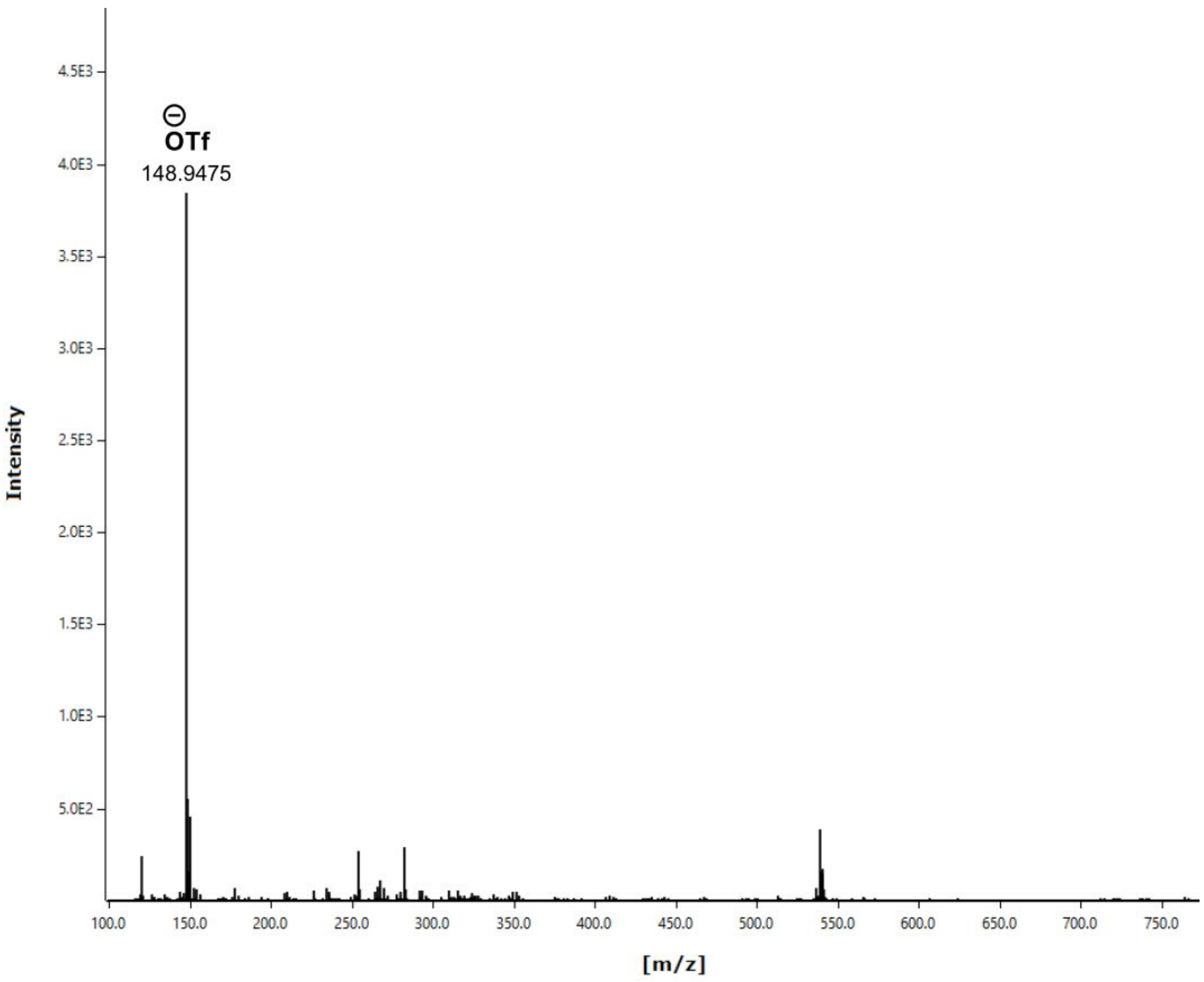

Figure S32: Mass spectrum (ESI, negative mode, methanol) of isolated cells, that were treated with **Ti4c** for 4 h.

NMR Spectra of complexes

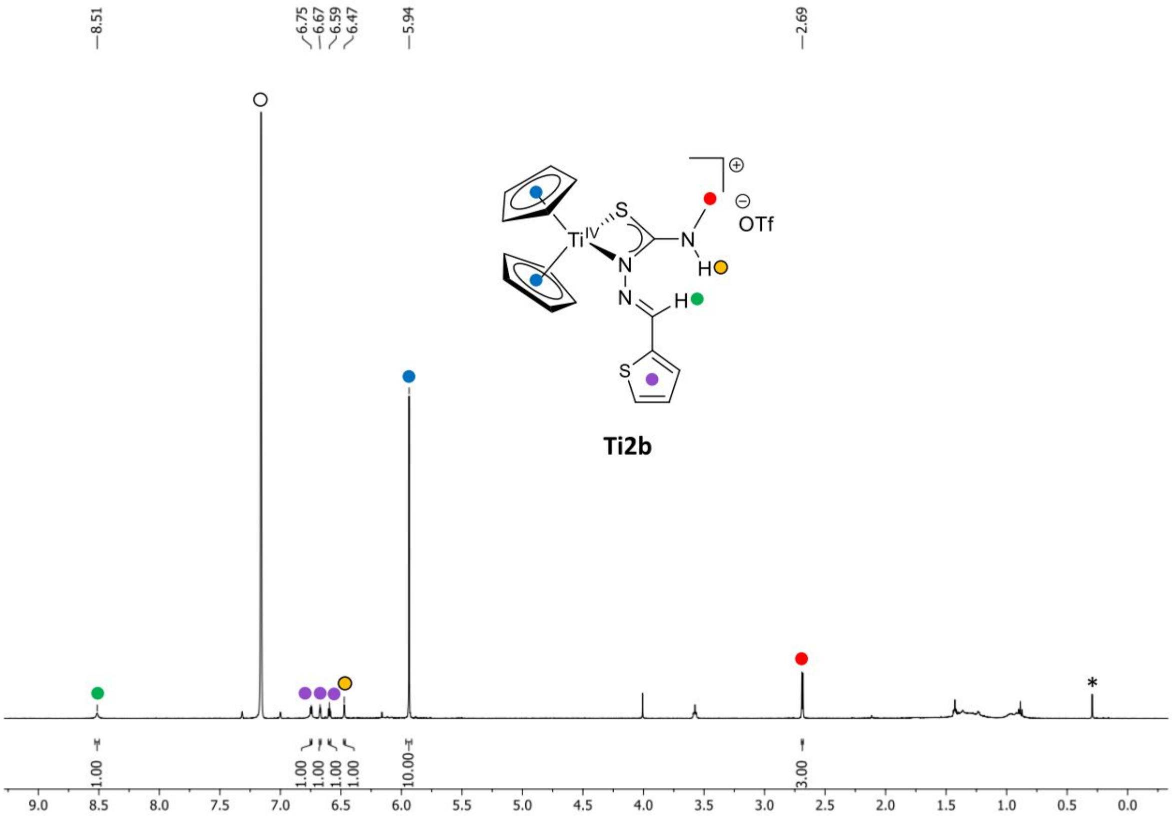

Figure S33: ^{1}H NMR spectrum (500 MHz, C_6D_6, 305 K) of **Ti2b**. Product signals given in colours (° = C_6H_5D, * = grease).

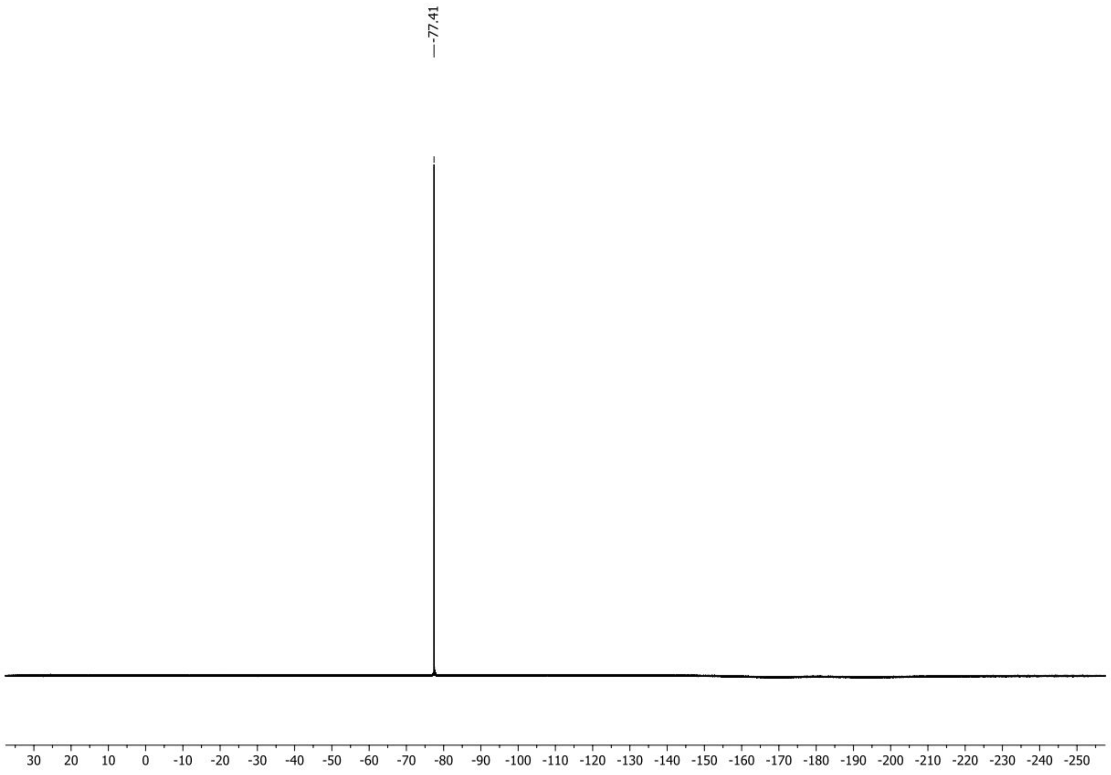

Figure S34: $^{19}F\{^{1}H\}$ NMR spectrum (470 MHz, C_6D_6, 305 K) of **Ti2b**.

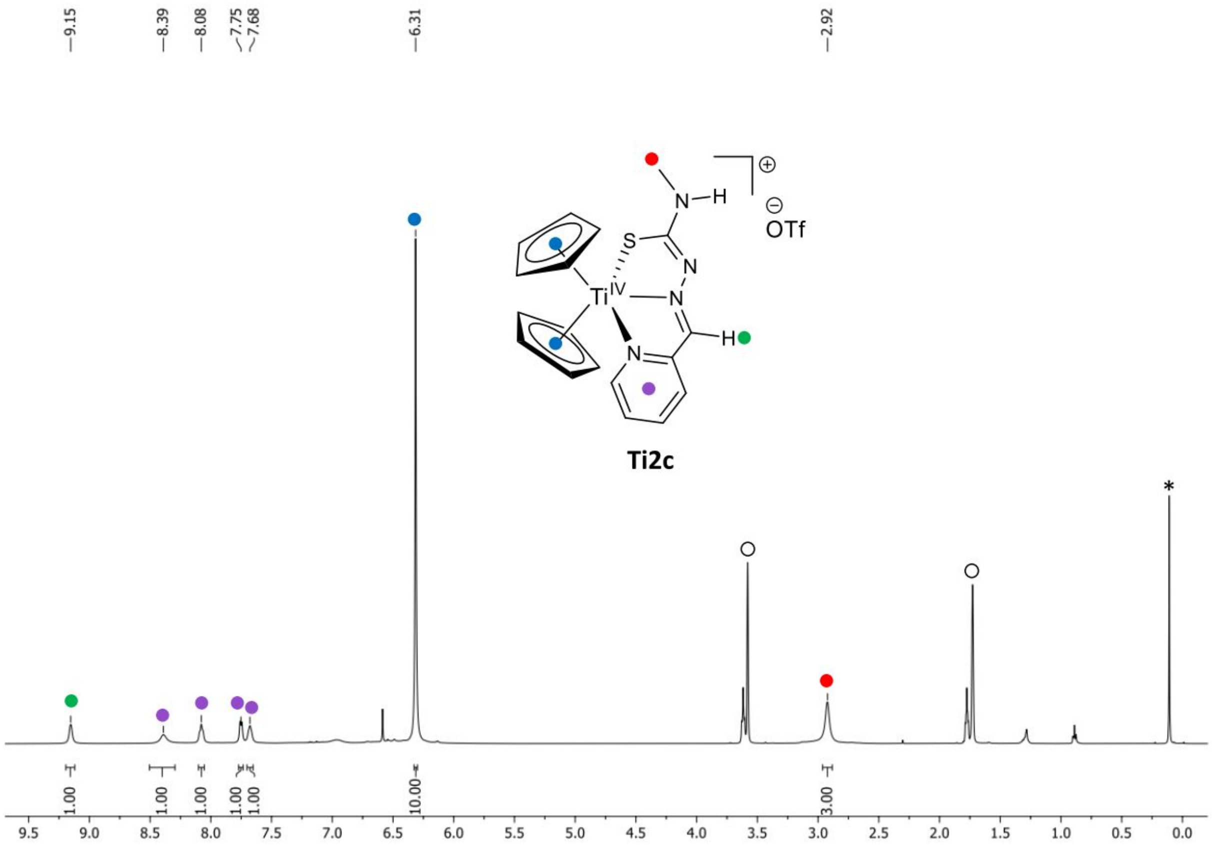

Figure S35: ^{1}H NMR spectrum (500 MHz, THF-*d*8, 298 K) of **Ti2c**. Product signals given in colours (^{o} = C4D_7HO, * = grease (contamination in THF-*d*8)).

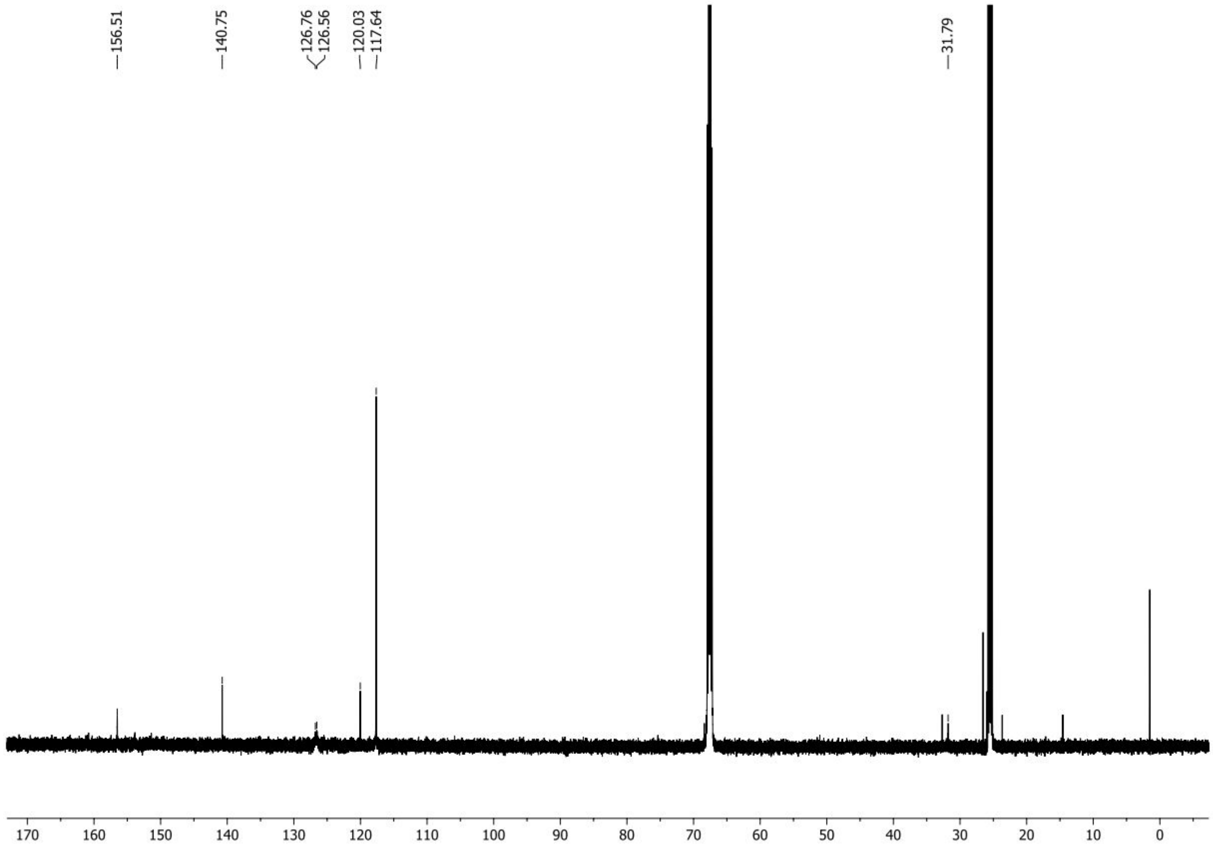

Figure S36: ^{13}C{^{1}H} NMR spectrum (125 MHz, THF-*d*8, 298 K) of **Ti2c**.

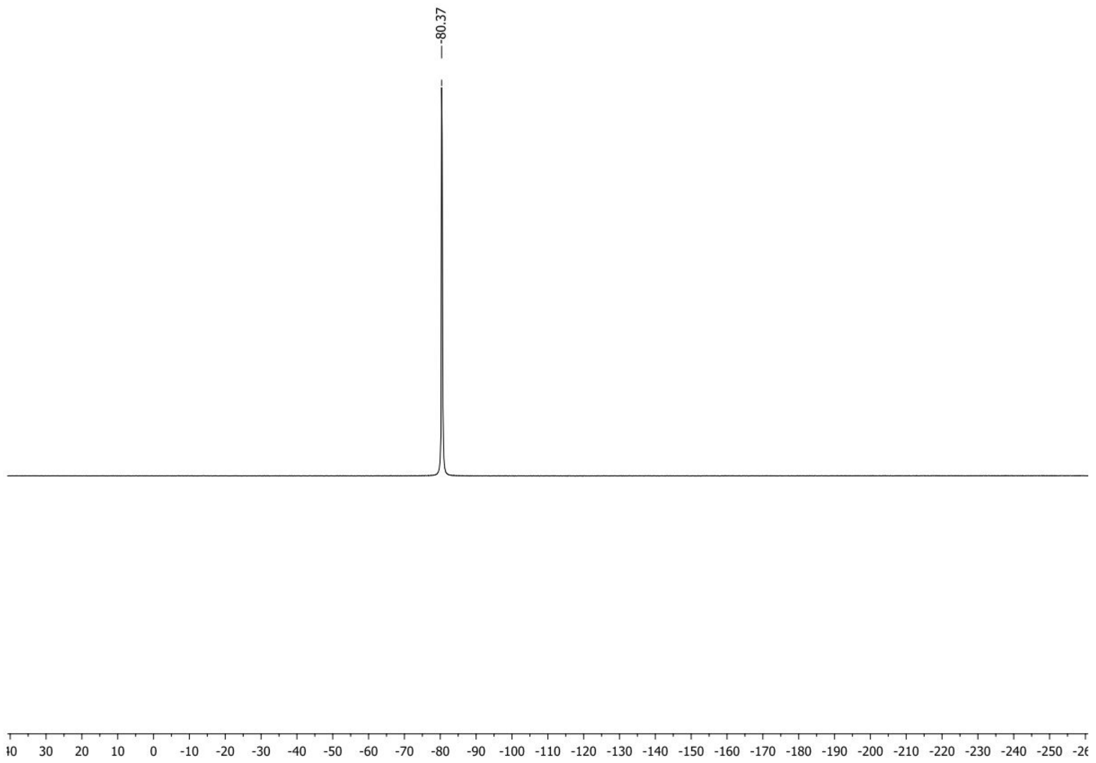

Figure S37: $^{19}F\{^{1}H\}$ NMR spectrum (470 MHz, THF-*d*8, 298 K) of **Ti2c**.

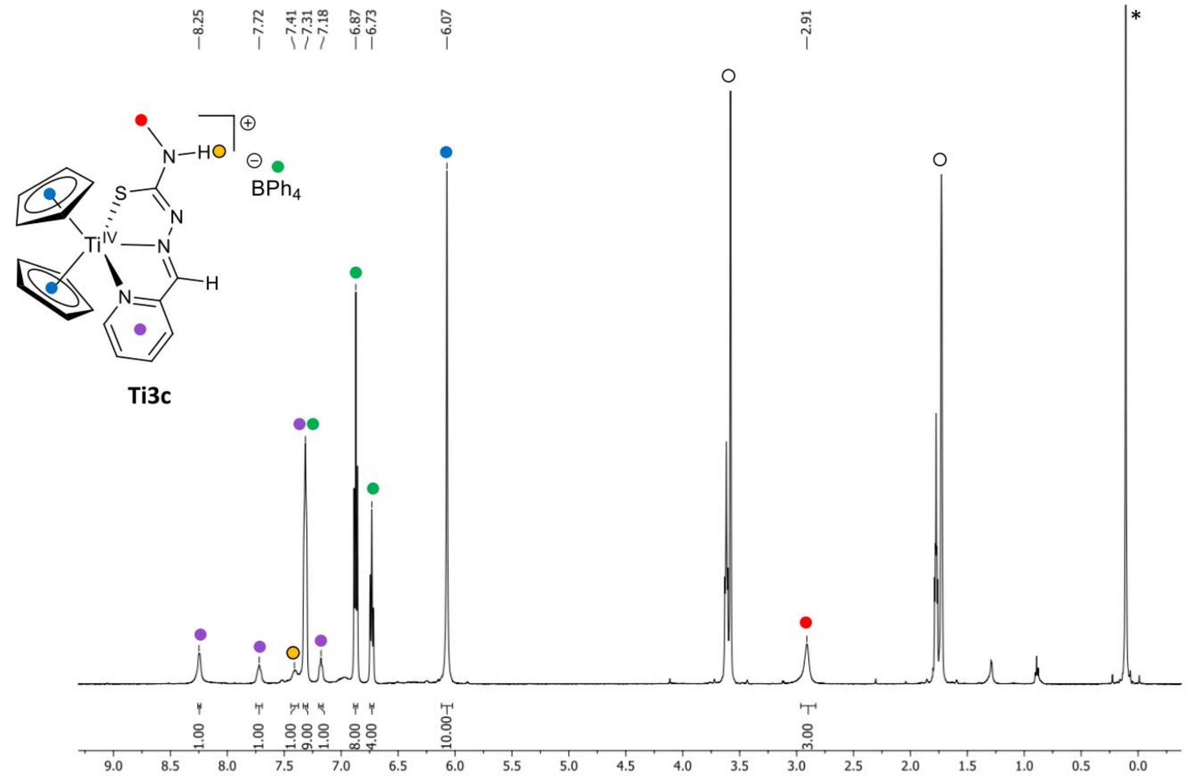

Figure S38: ^{1}H NMR spectrum (500 MHz, THF-*d*8, 298 K) of **Ti3c**. Product signals given in colours (° = $C4D_7HO$, * = grease (contamination in THF-*d*8)).

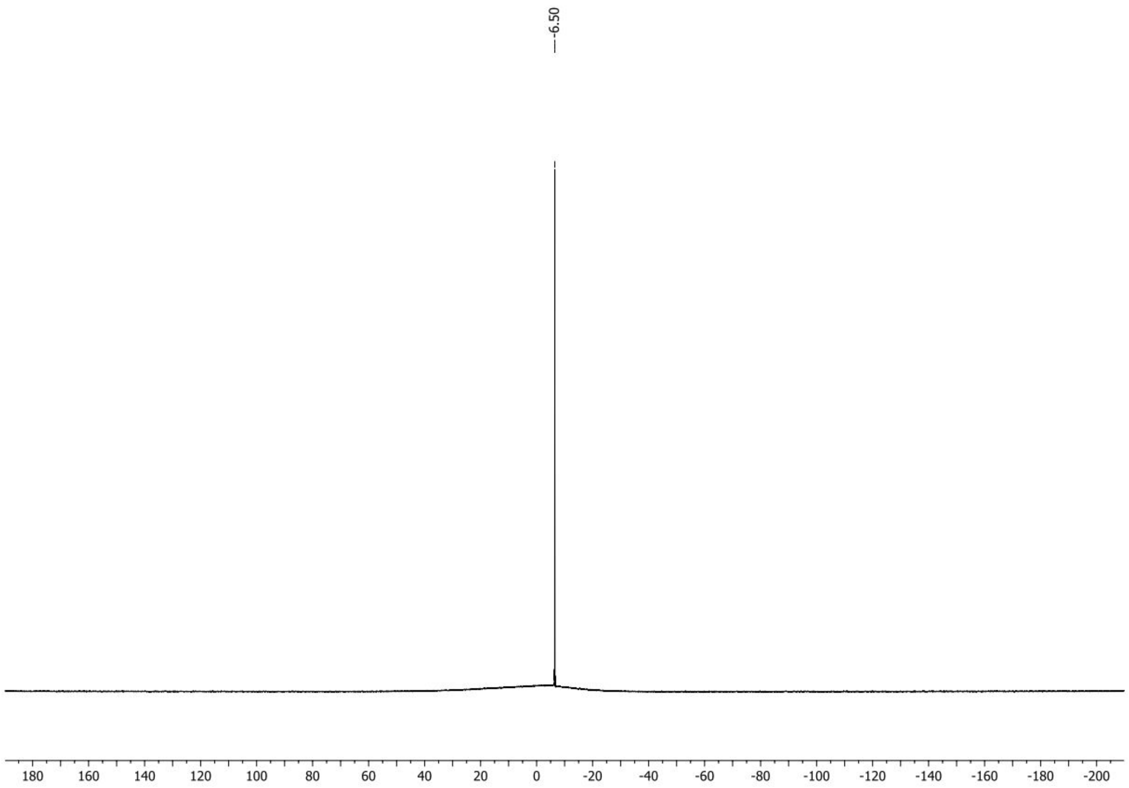

Figure S39: $^{11}B\{^{1}H\}$ NMR spectrum (160 MHz, THF-*d*8, 298 K) of **Ti3c**.

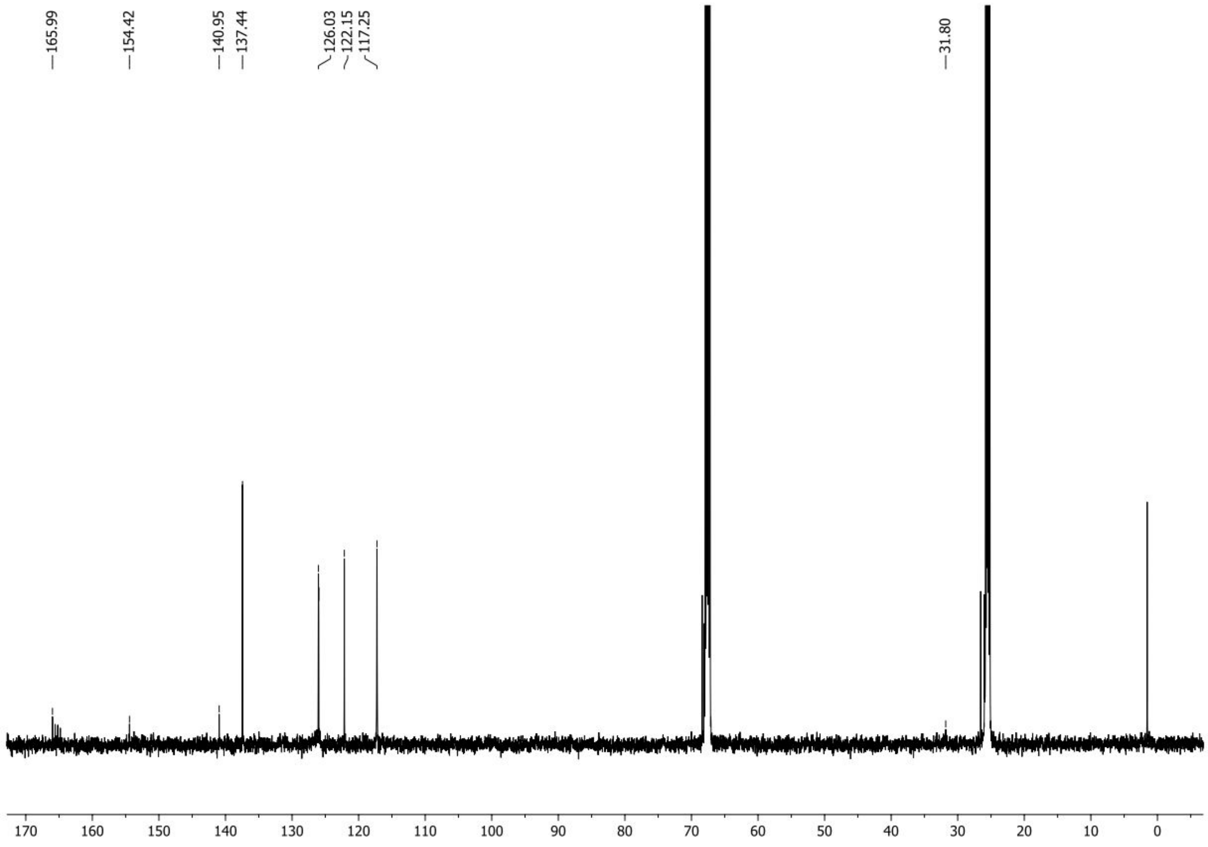

Figure S40: $^{13}C\{^{1}H\}$ NMR spectrum (125 MHz, THF-*d*8, 298 K) of **Ti3c**.

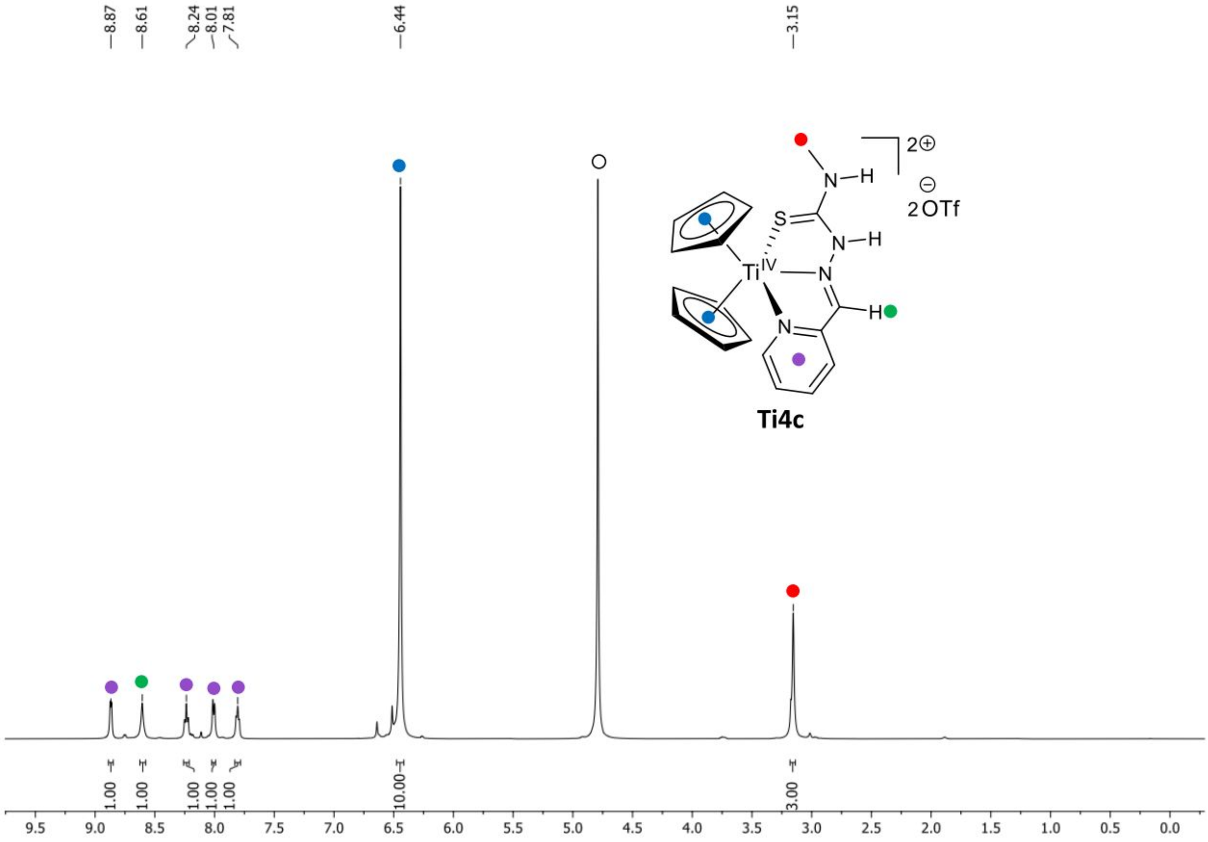

Figure S41: ^{1}H NMR spectrum (500 MHz, D_2O, 298 K) of **Ti4c**. Product signals given in colours (° = HDO).

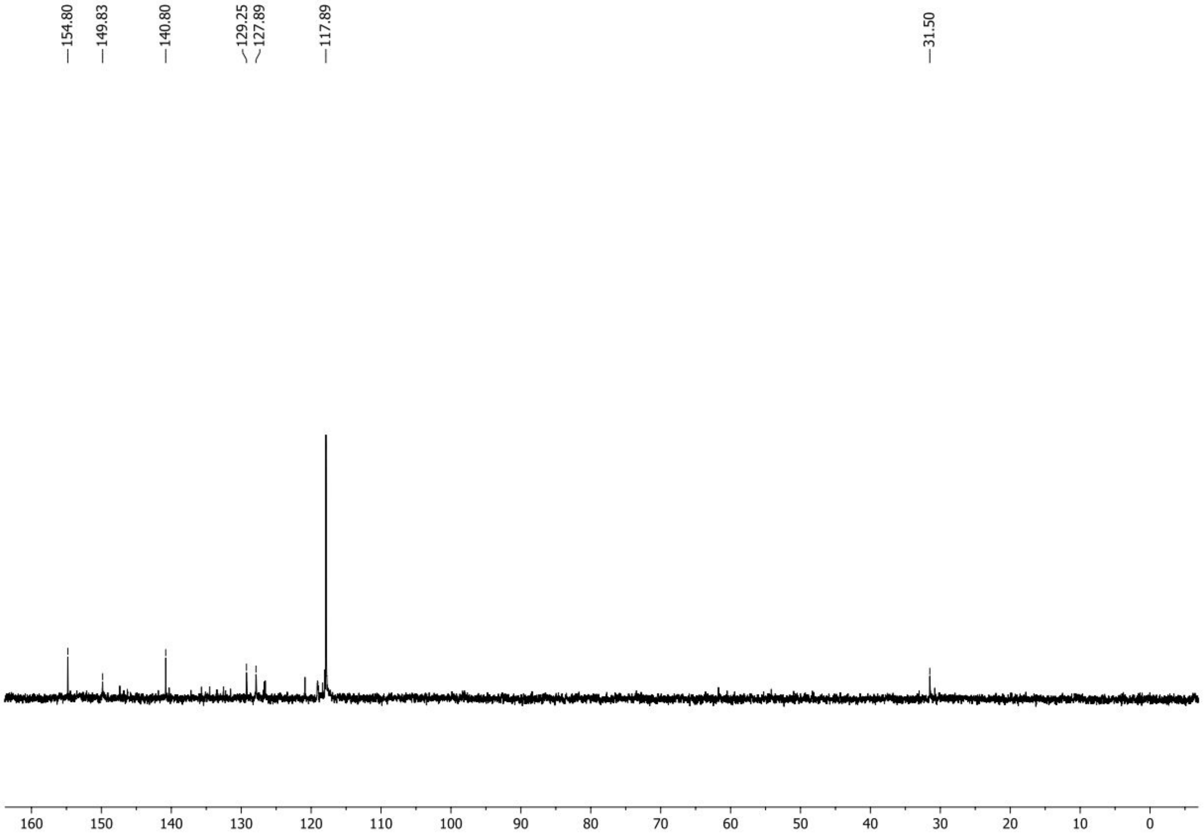

Figure S42: $^{13}C\{^{1}H\}$ NMR spectrum (125 MHz, D_2O, 298 K) of **Ti4c**.

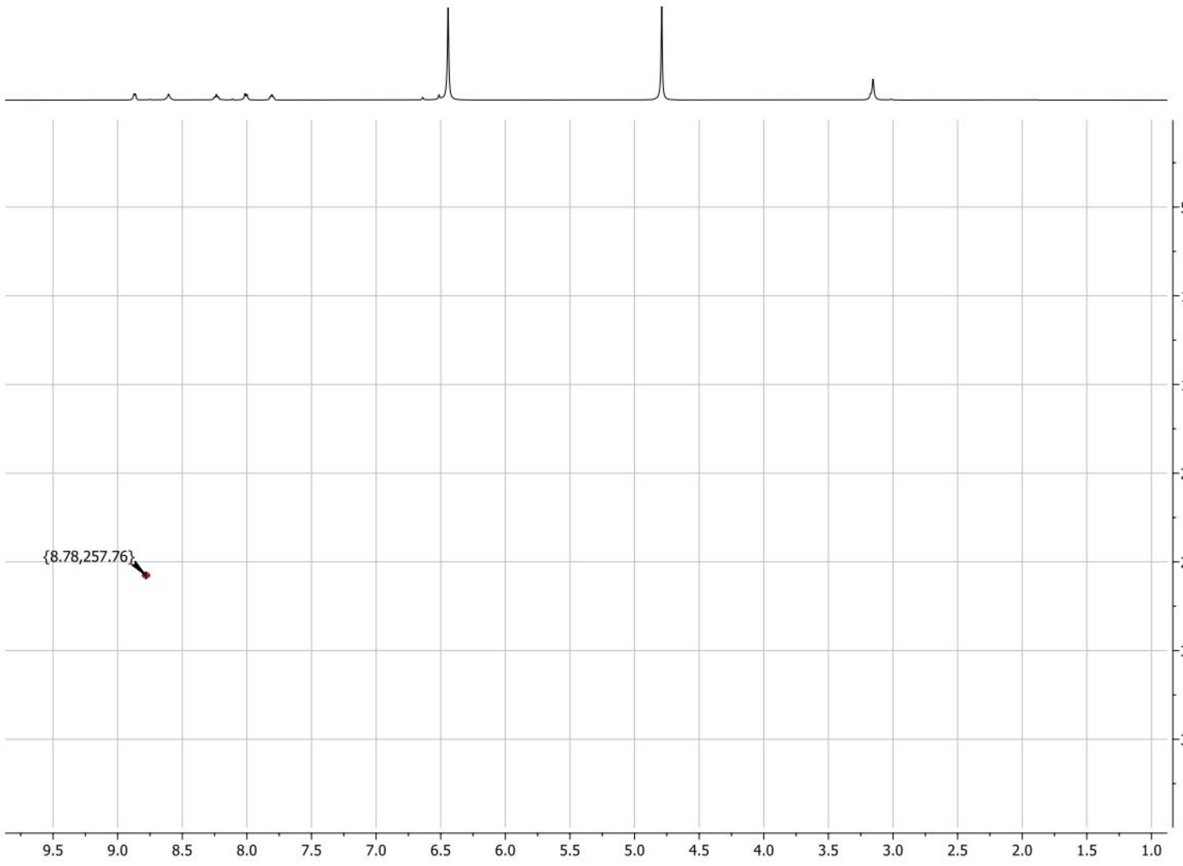

Figure S43: ^{1}H-^{15}N-HMBC NMR spectrum (51 MHz, D_2O, 298 K) of **Ti4c**.

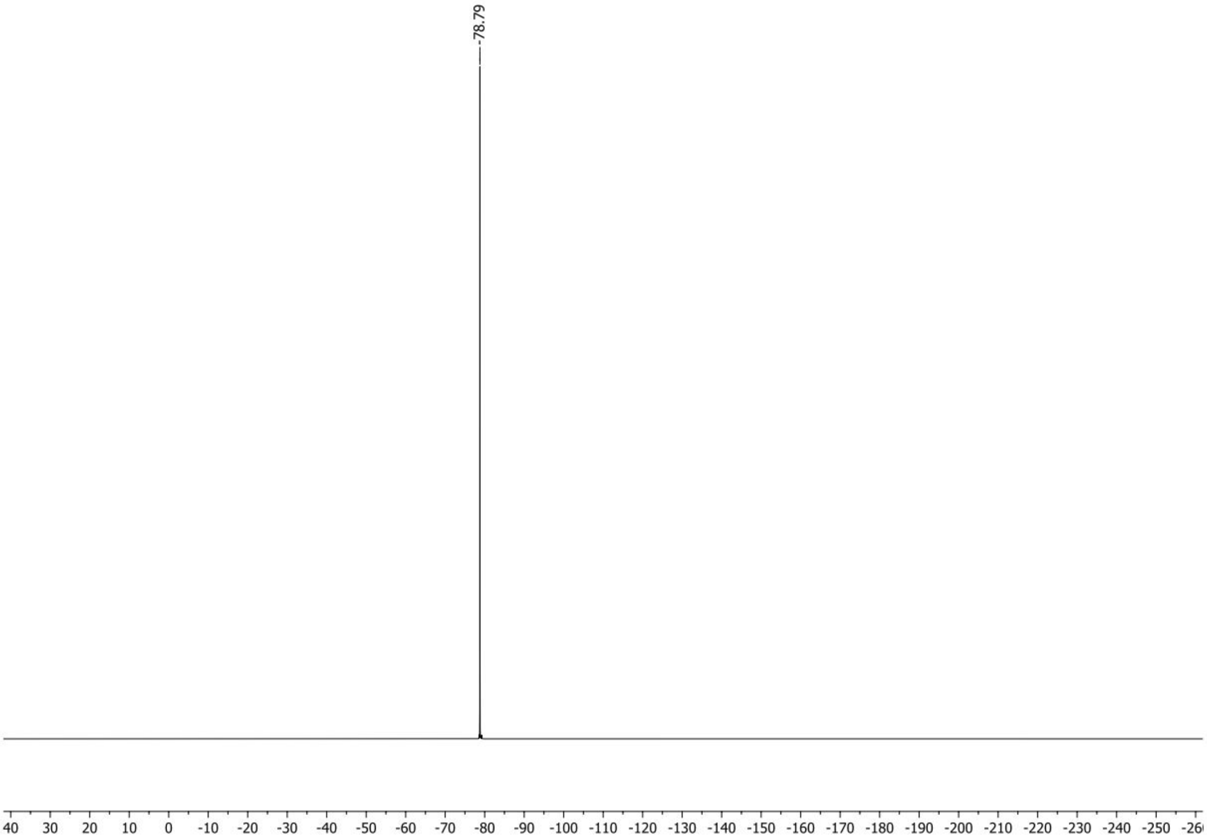

Figure S44: $^{19}F\{^{1}H\}$ NMR spectrum (470 MHz, D_2O, 298 K) of **Ti2c**.

Crystallographic data

Single crystal X-ray data were measured on a Bruker AXS D8 Venture diffractometer (multilayer optics, Mo-Kα and Cu-Kα radiation with λ = 0.71073 Å and 1.54178 Å respectively, Kappa 4-circle goniometer, Photon III C14 CPAD detector). All crystals were measured at a temperature of 100 K, except for **Ti2a** which was measured at 150 K, because below that temperature a slow reversible phase transition was observed broadening the reflections. Absorption corrections using equivalent reflections were performed with the program SADABS.[1] All structures were solved with the program SHELXS[2] and refined with SHELXL[3] using the OLEX2[4] GUI.

All non-H atoms were refined using anisotropic atomic displacement parameters (ADPs). The minor site of the disordered solvent molecule tetrahydrofuran in **Ti3c** were refined using isotropic ADPs. H atoms bonded to C were located in the difference Fourier maps and placed on idealized geometric positions with idealized ADPs using the riding model. H atoms bonded to N were refined freely.

The crystallographic data can be obtained free of charge from https://www.ccdc.cam.ac.uk/structures/ quoting the CCDC numbers 2420749-2420755.

Table S1: Crystallographic data of **Ti1a**, **Ti1b**, **Ti1c**, **Ti2a**.

	**Ti1a**	**Ti1b**	**Ti1c**	**Ti2a**
CCDC	2420750	2420753	2420754	2420749
Lab-ID	KESC260	KESC264	KESC266	KESC278
empirical formula	$C_{19}H_{20}N_3STi$	$C_{23}H_{24}N_3S_2Ti$	$C_{18}H_{19}N_4STi$	$C_{20}H_{20}F_3N_3O_3S_2Ti$
Fw	370.34	454.47	371.33	519.41
Colour	blue	pale purple yellow	green pink	green yellow
Habit	block	block	plate	plate
cryst. dimens. mm	0.10 x 0.07 x 0.04	0.08 x 0.08 x 0.05	0.14 x 0.05 x 0.15	0.12 x 0.11 x 0.02
cryst. system	monoclinic	monoclinic	monoclinic	monoclinic
space group	$P2_1/c$	$P2_1/n$	$P2_1/c$	$P2_1/n$
a, Å	16.4925(15)	8.6940(3)	8.8745(3)	10.2905(3)
b, Å	7.7326(7)	13.7355(5)	18.3662(5)	12.5949(3)
c, Å	13.8706(12)	18.0371(6)	10.5809(3)	17.3830(4)
α, deg	90	90	90	90
β, deg	92.633(3)	92.4931(12)	101.5778(11)	104.2686(10)
γ, deg	90	90	90	90
V, $Å^3$	1767.0(3)	2151.89(13)	1689.50(9)	2183.47(10)
Z	4	4	4	4
$D_{calc.}$, g cm^{-3}	1.392	1.403	1.460	1.580
μ, mm^{-1}	0.607	0.606	0.637	5.601
T, K	100(2)	100(2)	100(2)	150(2)
λ, Å	0.71073	0.71073	0.71073	1.54178
θ range, deg	2.472 – 32.032	1.864 – 36.316	2.218 – 34.969	4.383 – 74.494
reflections collected	76968	125227	103748	40915
Indep. Reflecions R(int)	6153 0.0460	10422 0.0361	7423 0.0513	4458 0.0372
Observed reflections (I > 2(I))	5297	9511	6522	4320
Absorption correction	semi-empirical	semi-empirical	semi-empirical	semi-empirical
max, min transm.	1.0000, 0.9210	1.0000, 0.9421	1.0000, 0.9330	1.0000, 0.7966
final R indices [$I>2\sigma(I)$]	R1 = 0.0441, wR2 = 0.1050	R1 = 0.0302, wR2 = 0.0742	R1 = 0.0340, wR2 = 0.0740	R1 = 0.0483, wR2 = 0.1164
R indices (all data)	R1 = 0.0531, wR2 = 0.1109	R1 = 0.0344, wR2 = 0.0763	R1 = 0.0419, wR2 = 0.0772	R1 = 0.0494, wR2 = 0.1170
GOF on F^2	1.091	1.071	1.088	1.247
largest diff peak / hole (e.Å- ³)	0.535 / -0.933	0.706 / -0.443	0.532 / -0.367	0.421 / -0.318

Table S2: Crystallographic data of **Ti2b**, **Ti3c**, **Ti4c**.

	**Ti2b**	**Ti3c**	**Ti4c**
CCDC	2420751	2420755	2420752
Lab-ID	KESC280B	KESC302	KESC326
empirical formula	$C_{18}H_{18}F_3N_3O_3S_3Ti$	$C_{50}H_{55}BN_4O_2STi$	$C_{20}H_{20}F_6N_4O_6S_3Ti$
Fw	525.43	834.75	670.48
Colour	green	red orange	yellow
Habit	plate	block	plate
cryst. dimens. mm	0.05 x 0.04 x 0.01	0.14 x 0.12 x 0.08	0.11 x 0.04 x 0.015
cryst. system	monoclinic	triclinic	orthorhombic
space group	$P2_1/c$	P-1	Pbca
a, Å	20.5090(5)	12.2190(4)	10.4813(7)
b, Å	12.9212(3)	13.5287(5)	15.8651(11)
c, Å	17.2109(4)	14.4906(5)	30.860(2)
α, deg	90	75.2756(12)	90
β, deg	112.1215(14)	85.0476(12)	90
γ, deg	90	67.3890(13)	90
V, $Å^3$	4225.16(18)	2138.49(13)	5131.6(6)
Z	8	2	8
$D_{calc.}$, g cm^{-3}	1.652	1.296	1.736
μ, mm^{-1}	6.696	0.295	5.944
T, K	100(2)	100(2)	100(2)
λ, Å	1.54178	0.71073	1.54178
θ range, deg	2.325 – 74.491	1.453 – 36.318	2.864 – 74.468
reflections collected	75481	182327	90762
Indep. Reflecions R(int)	8636<br>0.1027	20737<br>0.0336	5239<br>0.0795
Observed reflections (I > 2(I))	6867	18821	4691
Absorption correction	semi-empirical	semi-empirical	semi-empirical
max, min transm.	1.0000, 0.8860	1.0000, 0.9444	0.9985, 0.7619
final R indices [$I>2\sigma(I)$]	R1 = 0.0424, wR2 = 0.0934	R1 = 0.0329, wR2 = 0.0871	R1 = 0.0453, wR2 = 0.1229
R indices (all data)	R1 = 0.0594, wR2 = 0.1009	R1 = 0.0371, wR2 = 0.0897	R1 = 0.0499, wR2 = 0.1268
GOF on F^2	1.036	1.062	1.043
largest diff peak / hole (e.$Å^{-3}$)	0.939 / -0.393	0.604 / -0.353	0.894 / -0.602

Molecular / crystal structures

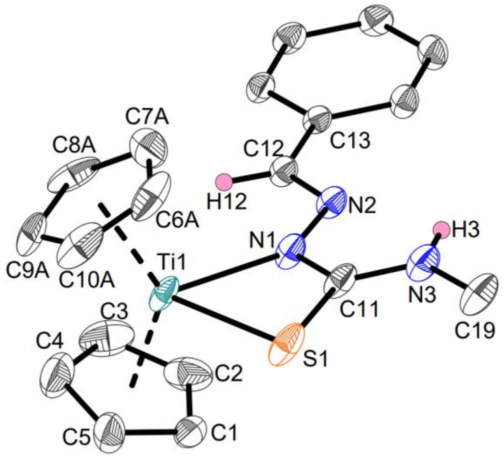

Figure S45: Molecular structure of complex **Ti1a**. Displacement ellipsoids are drawn at the 50% probability level. Redundant H atoms and solvent molecules have been omitted for clarity. Selected bond lengths (Å) and angles (deg): Ti1–N1 2.1563(13), Ti1–S1 2.6115(5), N1–N2 1.3807(16), N2–C12 1.2863(18), N1–C11 1.3351(19), N3–C11 1.331(2), S1–C11 1.7173(15), N1–Ti1–S1 63.86(3), N1–C11–S1 112.80(12), Ti1–N1–N2 140.27(9), C11–N1–N2 114.63(12), Ct1–Ti1–Ct2 131.9 (Ct1 = centroid of C1–C5; Ct2 = centroid of C6–C10).

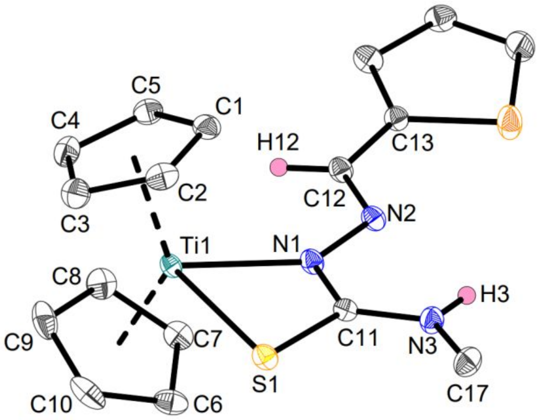

Figure S46: Molecular structure of complex **Ti1b**. Displacement ellipsoids are drawn at the 50% probability level. Redundant H atoms and solvent molecules have been omitted for clarity. Selected bond lengths (Å) and angles (deg): Ti1–N1 2.1656(7), Ti1–S1 2.5938(2), N1–N2 1.3809(9), N2–C12 1.2912(10), N1–C11 1.3375(10), N3–C11 1.3378(10), S1–C11 1.7211(8), N1–Ti1–S1 64.029(18), N1–C11–S1 112.30(6), Ti1–N1–N2 139.78(5), C11–N1–N2 114.91(6), Ct1–Ti1–Ct2 134.8 (Ct1 = centroid of C1–C5; Ct2 = centroid of C6–C10).

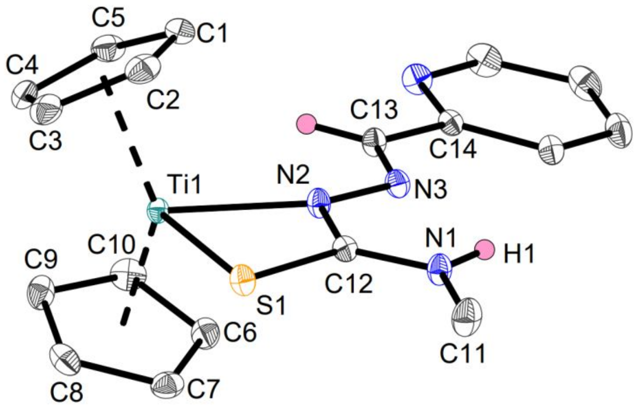

Figure S47: Molecular structure of complex **Ti1c**. Displacement ellipsoids are drawn at the 50% probability level. Redundant H atoms and solvent molecules have been omitted for clarity. Selected bond lengths (Å) and angles (deg): Ti1–N2 2.1532(9), Ti1–S1 2.6093(3), N2–N3 1.3725(12), N3–C13 1.2891(13), N2–C12 1.3434(13), N1–C12 1.3323(13), S1–C12 1.7218(10), N2–Ti1–S1 63.95(2), N2–C12–S1 112.11(7), Ti1–N2–N3 139.17(7), C12–N2–N3 114.19(8), Ct1–Ti1–Ct2 136.1 (Ct1 = centroid of C1–C5; Ct2 = centroid of C6–C10).

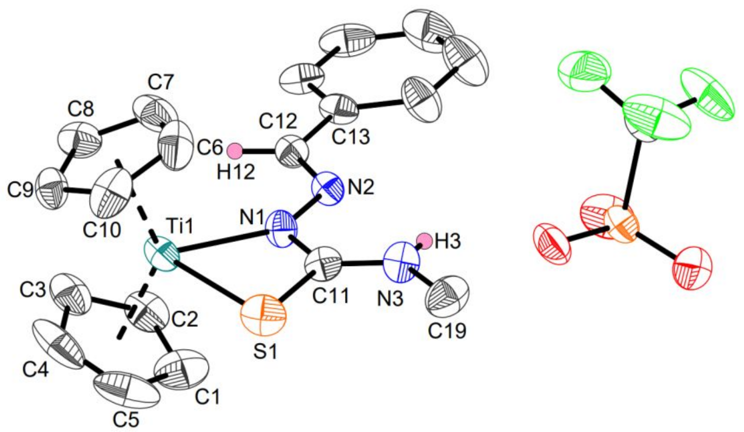

Figure S48: Crystal structure of complex **Ti2a**. Displacement ellipsoids are drawn at the 50% probability level. Redundant H atoms have been omitted for clarity. Selected bond lengths (Å) and angles (deg): Ti1–N1 2.117(2), Ti1–S1 2.4992(11), N1–N2 1.381(3), N2–C12 1.280(4), N1–C11 1.335(4), N3–C11 1.307(4), S1–C11 1.738(3), N1–Ti1–S1 67.06(7), N1–C11–S1 112.8(2), Ti1–N1–N2 143.99(19), C11–N1–N2 113.8(2), Ct1–Ti1–Ct2 134.5 (Ct1 = centroid of C1–C5; Ct2 = centroid of C6–C10).

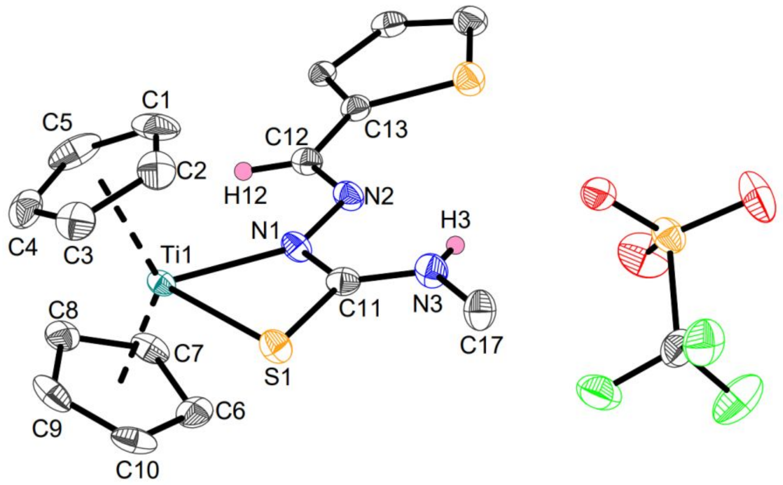

Figure S49: Crystal structure of complex **Ti2b**. Displacement ellipsoids are drawn at the 50% probability level. Redundant H atoms have been omitted for clarity. Selected bond lengths (Å) and angles (deg): Ti1–N1 2.114(2), Ti1–S1 2.5034(9), N1–N2 1.386(3), N2–C12 1.290(4), N1–C11 1.329(4), N3–C11 1.324(4), S1–C11 1.743(3), N1–Ti1–S1 66.85(7), N1–C11–S1 112.4(2), Ti1–N1–N2 143.24(19), C11–N1–N2 113.8(2), Ct1–Ti1–Ct2 133.4 (Ct1 = centroid of C1–C5; Ct2 = centroid of C6–C10).

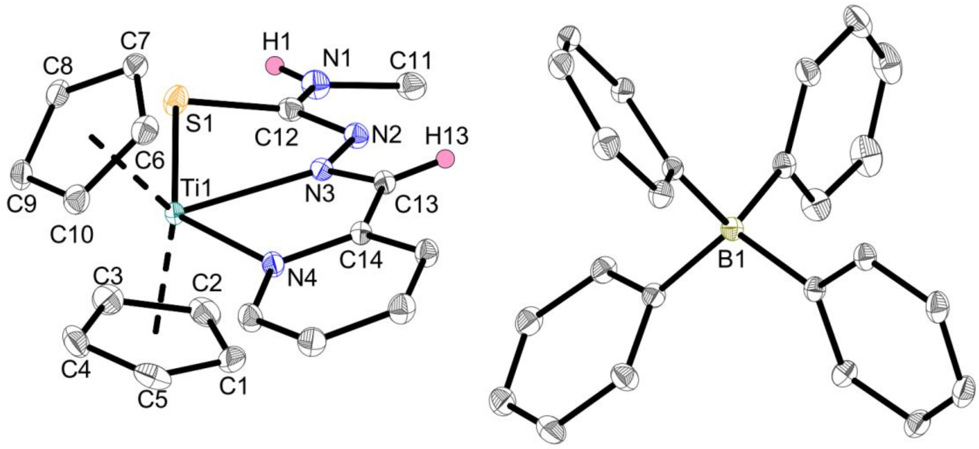

Figure S50: Crystal structure of complex **Ti3c**. Displacement ellipsoids are drawn at the 50% probability level. Redundant H atoms and solvent molecules have been omitted for clarity. Selected bond lengths (Å) and angles (deg): Ti1–N3 2.2641(6), Ti1–N4 2.3591(6), Ti1–S1 2.5766(2), N2–N3 1.3736(8), N2–C12 1.3223(9), N1–C11 1.4527(10), N3–C13 1.2967(8), N4–C14 1.3539(8), S1–C12 1.7289(7), N3–Ti1–S1 70.934(15), N3–Ti1–N4 69.64(2), N1–C12–S1 123.59(5), Ti1–N1–N2 126.98(4), C12–N2–N3 112.52(6), Ct1–Ti1–Ct2 132.1 (Ct1 = centroid of C1–C5; Ct2 = centroid of C6–C10).

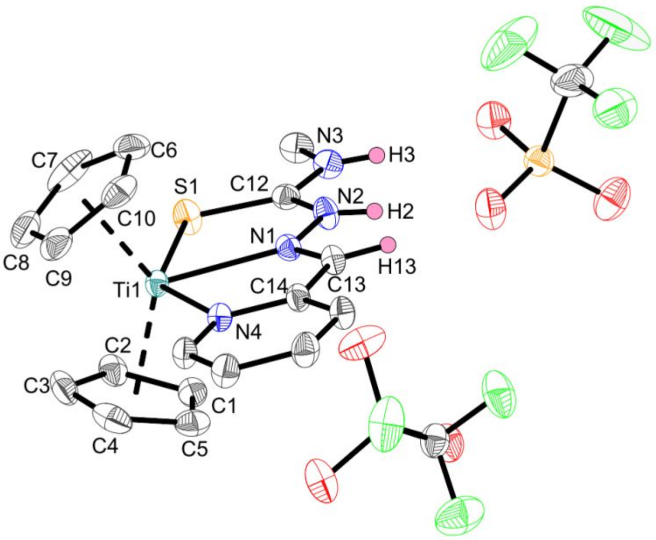

Figure S51: Crystal structure of complex **Ti4c**. Displacement ellipsoids are drawn at the 50% probability level. Redundant H atoms and solvent molecules have been omitted for clarity. Selected bond lengths (Å) and angles (deg): Ti1–N1 2.280(2), Ti1–N4 2.316(2), Ti1–S1 2.6598(8), N1–N2 1.366(3), N2–C12 1.348(4), N1–C13 1.290(4), N3–C12 1.320(4), N4–C14 1.357(3), S1–C12 1.685(3), N1–Ti1–S1 71.22(6), N1–Ti1–N4 69.60(8), N3–C12–S1 124.5(2), Ti1–N1–N2 125.53(17), C12–N2–N1 120.7(2), Ct1–Ti1–Ct2 131.4 (Ct1 = centroid of C1–C5; Ct2 = centroid of C6–C10).

IR spectra

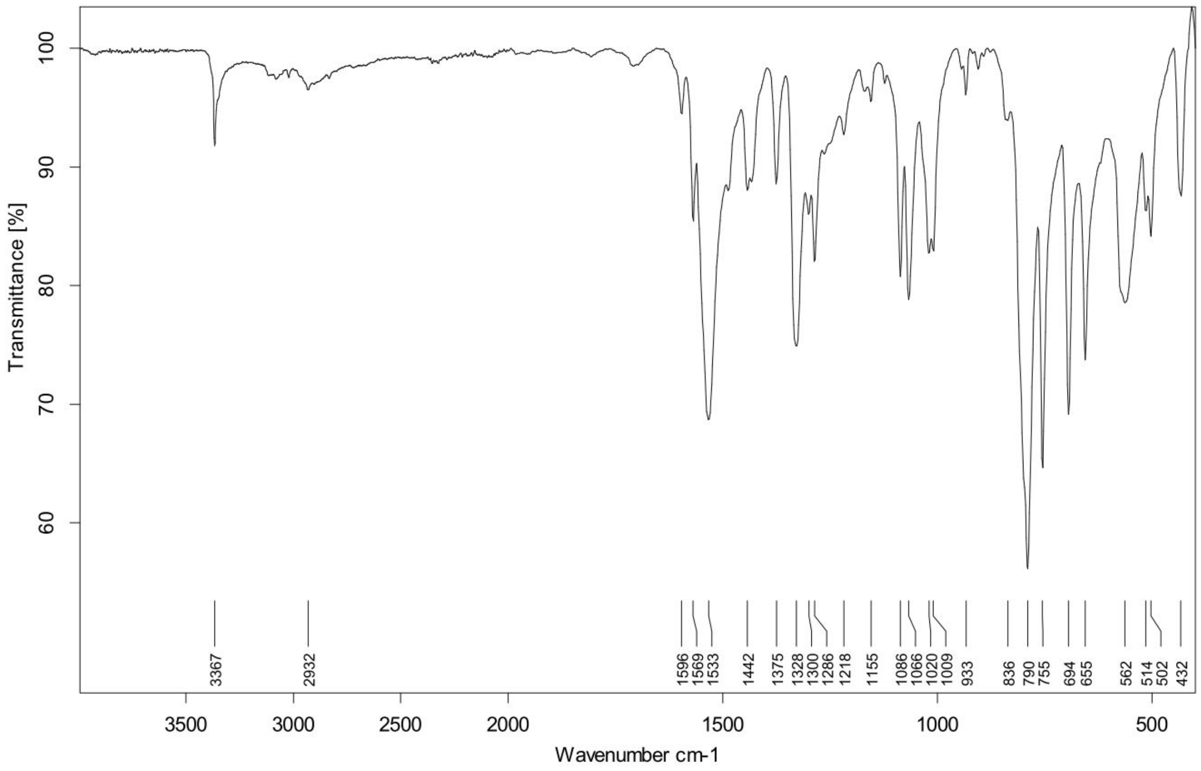

Figure S52: IR Spectrum of **Ti1a**.

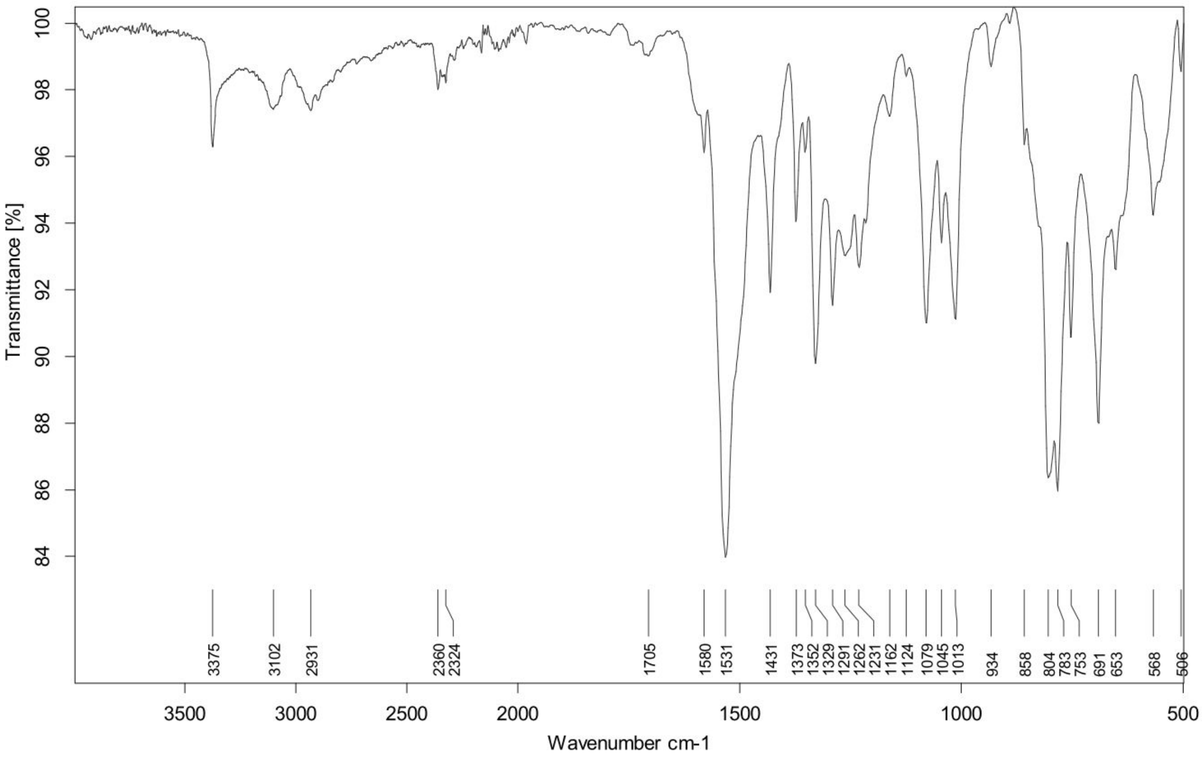

Figure S53: IR Spectrum of **Ti1b**.

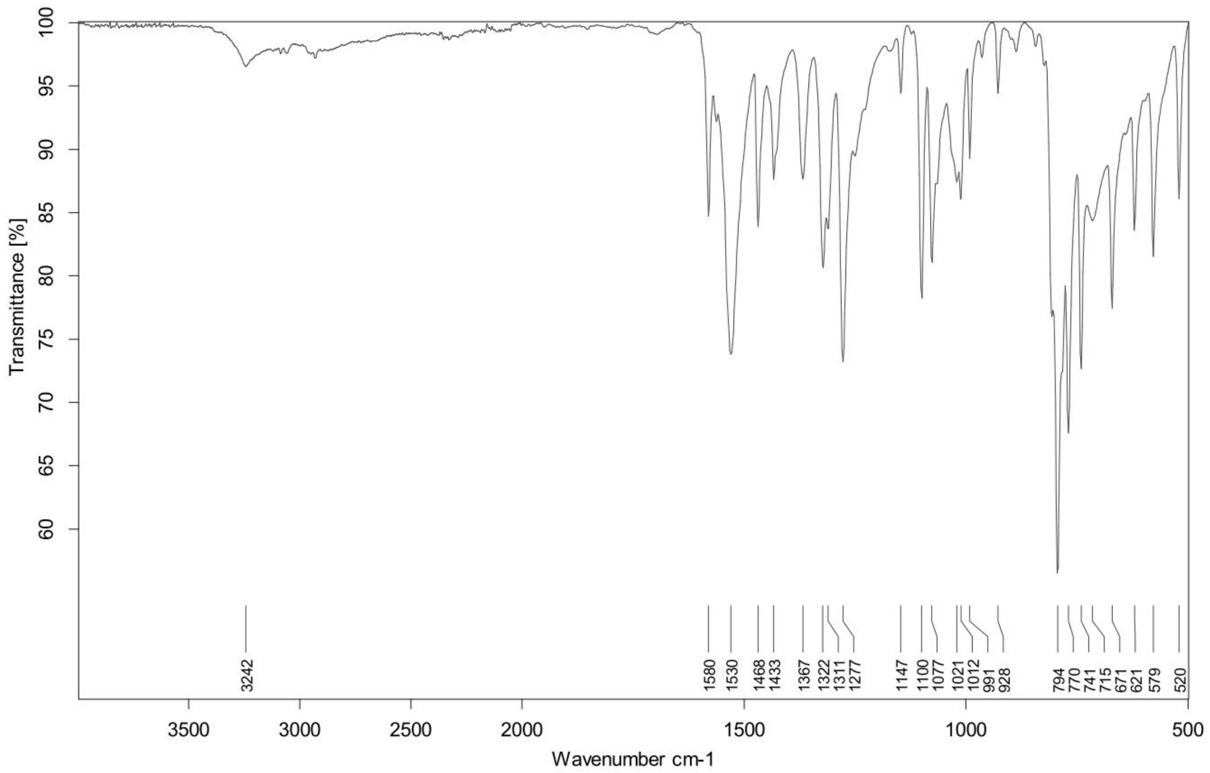

Figure S54: IR Spectrum of **Ti1c**.

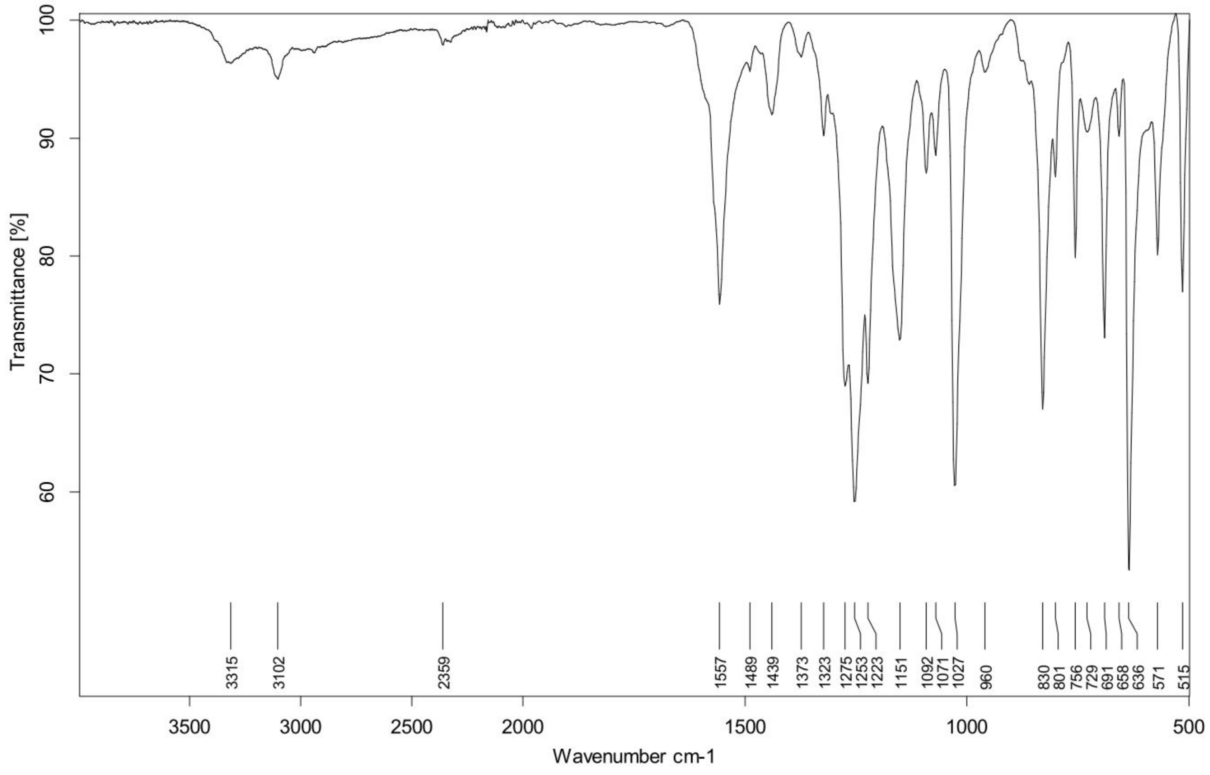

Figure S55: IR Spectrum of **Ti2a**.

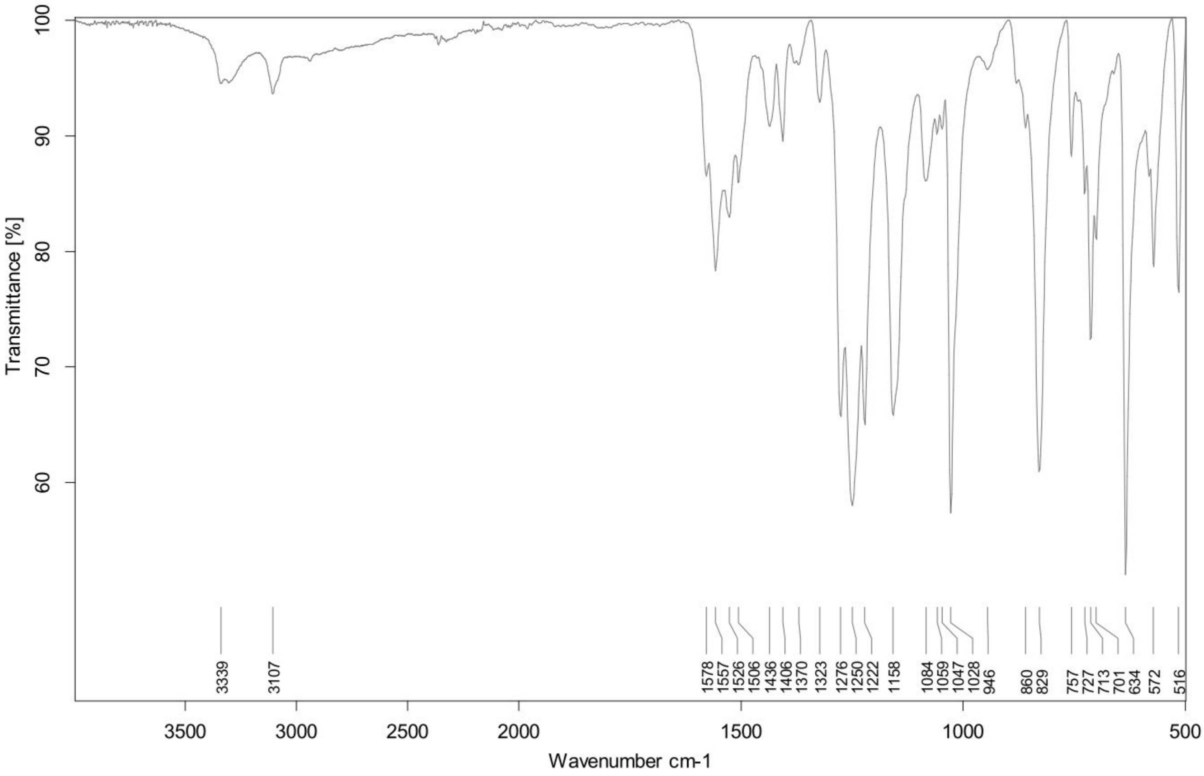

Figure S56: IR Spectrum of **Ti2b**.

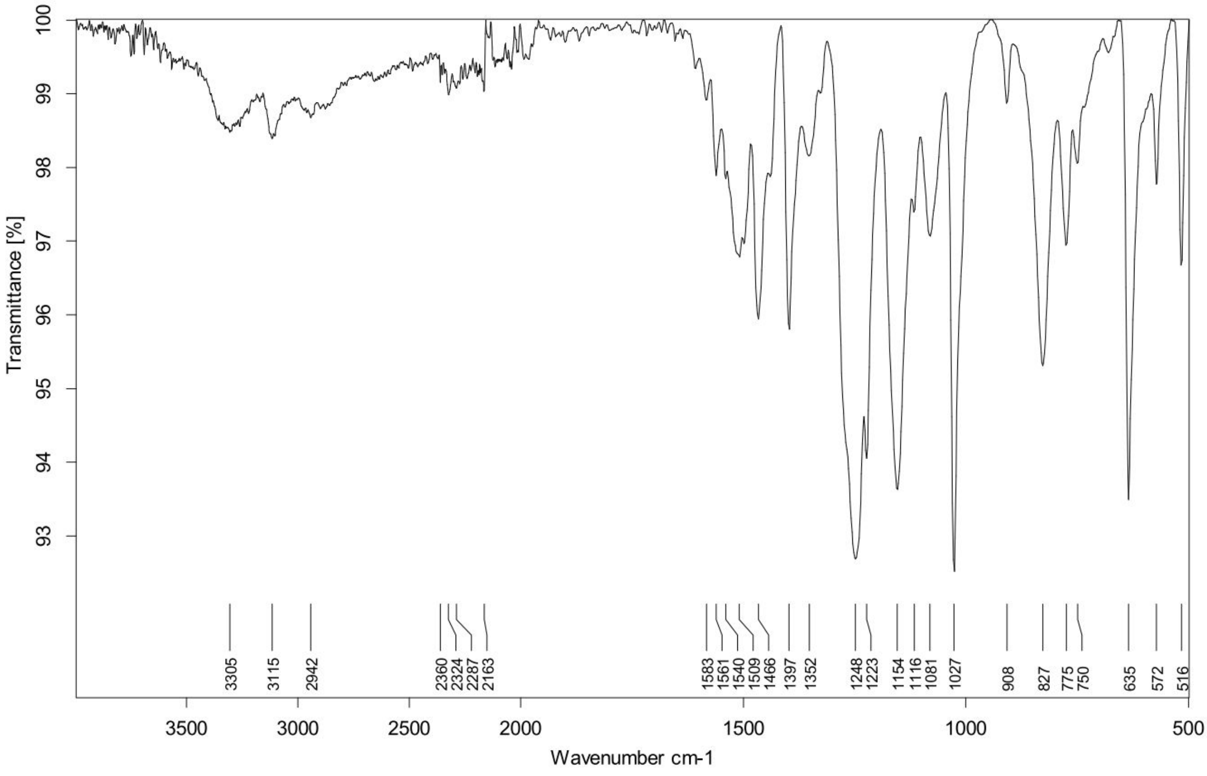

Figure S57: IR Spectrum of **Ti2c**.

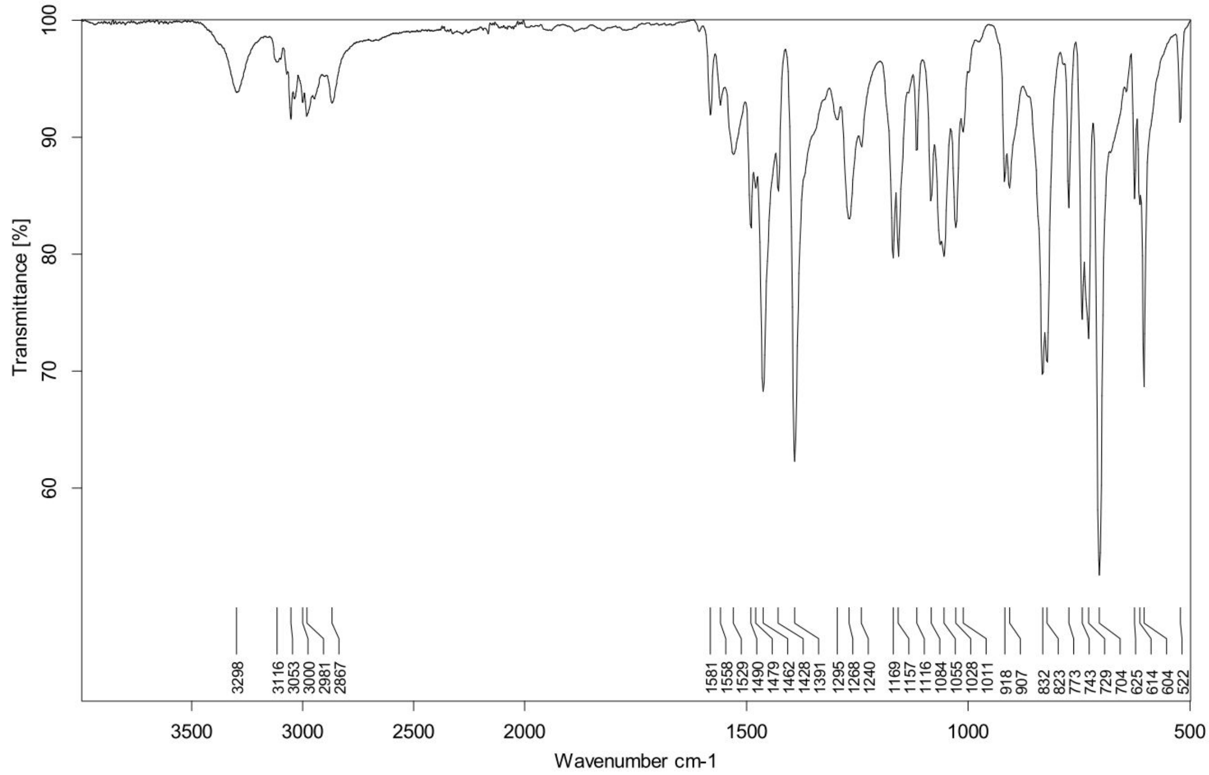

Figure S58: IR Spectrum of **Ti3c**.

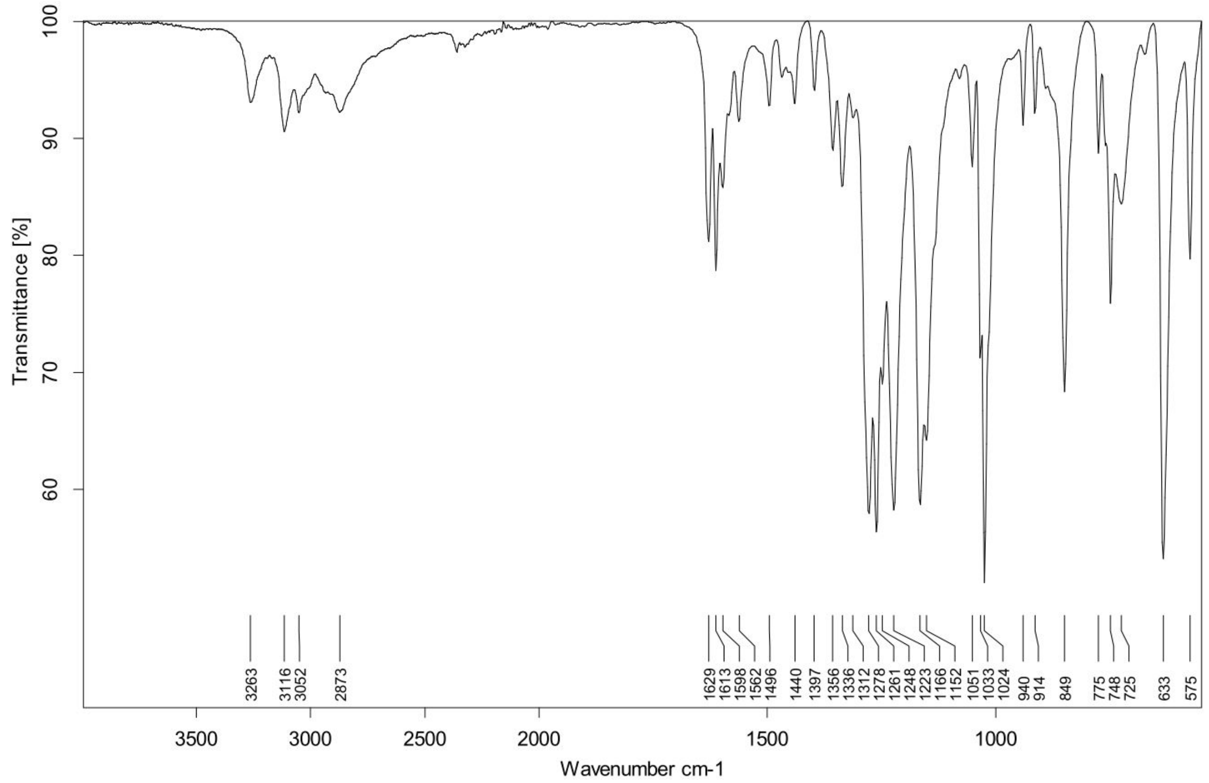

Figure S59: IR Spectrum of **Ti4c**.

Apoptosis studies

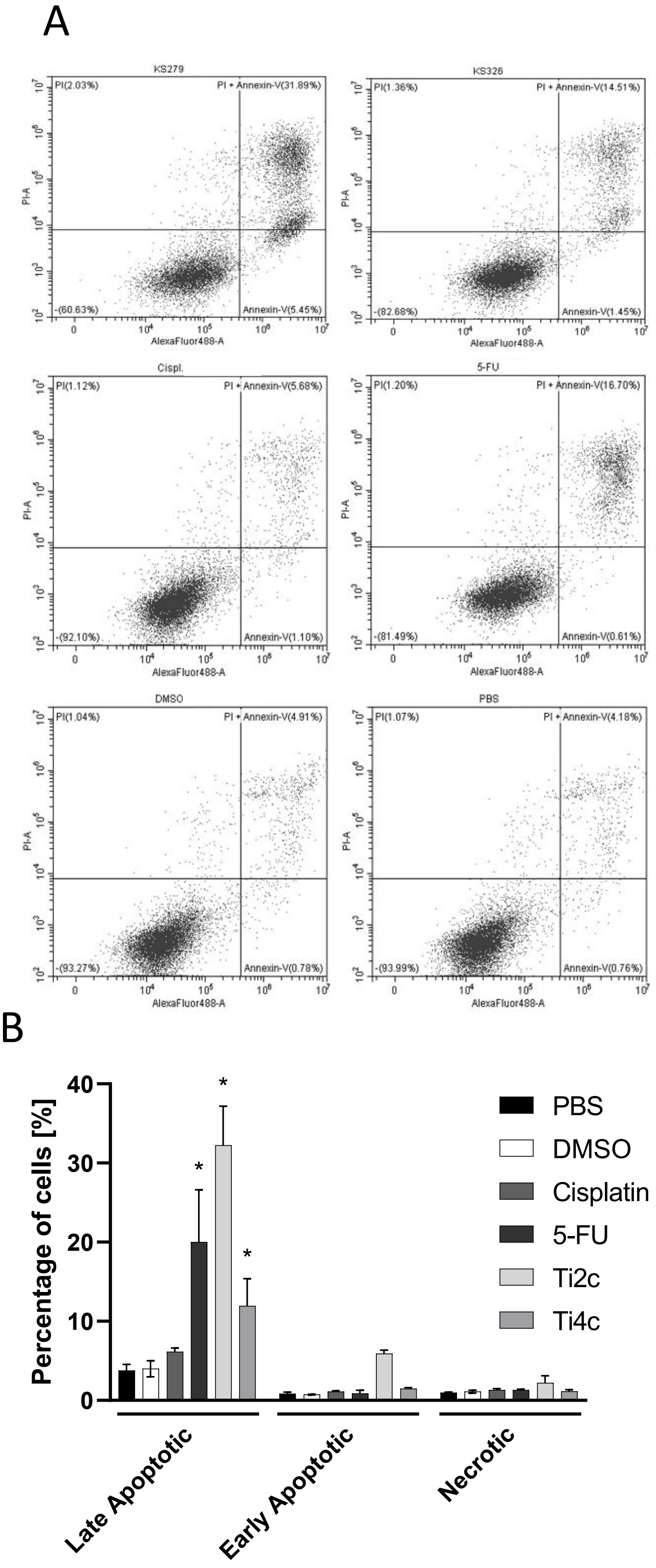

Figure S60. Enlarged plots of **Figure 7**: Apoptotic effect of the synthesized compounds quantified by Annexin V-PI staining and flow cytometry analysis. HCT116 cells were incubated with **Ti2c**, **Ti4c** or Cisplatin, 5-FU at 10 µM or the corresponding controls over 48 h. **A:** Examples of original flow cytometry measurements as labeled above in the respective scatter-plot. PI = Necrotic cells, PI+Annexin = Late Apoptotic cells, - = Live cells, Annexin-V = Early Apoptotic cells. **B:** Quantification from n = 3. Values are expressed as mean ± SD, *= p-value < 0.0001 compared to DPBS control. KS279 = **Ti2c**, KS329 = **Ti4c**.

References

[1] L. Krause, R. Herbst-Irmer, G. M. Sheldrick, D. Stalke, *J. Appl. Cryst.* **2015**, *48*, 3-10.

[2] G. Sheldrick; A short history of SHELX. *Acta Cryst. A* **2008**, *64*, 112-122.

[3] G. Sheldrick; Crystal structure refinement with SHELXL. *Acta Cryst. C* **2015**, *71*, 3-8.

[4] O. V. Dolomanov, L. J. Bourhis, R. J. Gildea, J. A. K. Howard, H. Puschmann, *J. Appl. Cryst.* **2009**, *42*, 339-341.
